# FBW7/GSK3β mediated degradation of IGF2BP2 inhibits IGF2BP2-SLC7A5 positive feedback loop and radioresistance in lung cancer

**DOI:** 10.1186/s13046-024-02959-3

**Published:** 2024-01-29

**Authors:** Zhiyuan Zhou, Bin Zhang, Yue Deng, Suke Deng, Jie Li, Wenwen Wei, Yijun Wang, Jiacheng Wang, Zishan Feng, Mengjie Che, Xiao Yang, Jingshu Meng, Yan Li, Yan Hu, Yajie Sun, Lu Wen, Fang Huang, Yuhan Sheng, Chao Wan, Kunyu Yang

**Affiliations:** 1 Cancer Center, Union Hospital, Tongji Medical College, Huazhong University of Science and Technology, Wuhan, 430022 China; 2 Hubei Key Laboratory of Precision Radiation Oncology, Wuhan, 430022 China; 3 Institute of Radiation Oncology, Union Hospital, Tongji Medical College, Huazhong University of Science and Technology, Wuhan, 430022 China

**Keywords:** IGF2BP2, SLC7A5, FBW7, N^6^-Methyladenosine, Radioresistance, Lung cancer

## Abstract

**Background:**

The development of radioresistance seriously hinders the efficacy of radiotherapy in lung cancer. However, the underlying mechanisms by which radioresistance occurs are still incompletely understood. The N^6^-Methyladenosine (m^6^A) modification of RNA is involved in cancer progression, but its role in lung cancer radioresistance remains elusive. This study aimed to identify m^6^A regulators involved in lung cancer radiosensitivity and further explore the underlying mechanisms to identify therapeutic targets to overcome lung cancer radioresistance.

**Methods:**

Bioinformatic mining was used to identify the m^6^A regulator IGF2BP2 involved in lung cancer radiosensitivity. Transcriptome sequencing was used to explore the downstream factors. Clonogenic survival assays, neutral comet assays, Rad51 foci formation assays, and Annexin V/propidium iodide assays were used to determine the significance of FBW7/IGF2BP2/SLC7A5 axis in lung cancer radioresistance. Chromatin immunoprecipitation (ChIP)-qPCR analyses, RNA immunoprecipitation (RIP) and methylated RNA immunoprecipitation (MeRIP)-qPCR analyses, RNA pull-down analyses, co-immunoprecipitation analyses, and ubiquitination assays were used to determine the feedback loop between IGF2BP2 and SLC7A5 and the regulatory effect of FBW7/GSK3β on IGF2BP2. Mice models and tissue microarrays were used to verify the effects in vivo.

**Results:**

We identified IGF2BP2, an m^6^A “reader”, that is overexpressed in lung cancer and facilitates radioresistance. We showed that inhibition of IGF2BP2 impairs radioresistance in lung cancer both in vitro and in vivo. Furthermore, we found that IGF2BP2 enhances the stability and translation of *SLC7A5* mRNA through m^6^A modification, resulting in enhanced SLC7A5-mediated transport of methionine to produce S-adenosylmethionine. This feeds back upon the *IGF2BP2* promoter region by further increasing the trimethyl modification at lysine 4 of histone H3 (H3K4me3) level to upregulate IGF2BP2 expression. We demonstrated that this positive feedback loop between IGF2BP2 and SLC7A5 promotes lung cancer radioresistance through the AKT/mTOR pathway. Moreover, we found that the ubiquitin ligase FBW7 functions with GSK3β kinase to recognize and degrade IGF2BP2.

**Conclusions:**

Collectively, our study revealed that the m^6^A “reader” IGF2BP2 promotes lung cancer radioresistance by forming a positive feedback loop with SLC7A5, suggesting that IGF2BP2 may be a potential therapeutic target to control radioresistance in lung cancer.

**Supplementary Information:**

The online version contains supplementary material available at 10.1186/s13046-024-02959-3.

## Background

Lung cancer has become the second most commonly diagnosed cancer and remains the leading cause of cancer-related mortality, with an estimated 2.2 million new cases and 1.8 million deaths worldwide in 2020 [1], and an estimated 238 thousand new cases and 127 thousand deaths in the United States in 2023 [2]. Non-small cell lung cancer (NSCLC) represents approximately 85% of lung cancer cases [3], and lung adenocarcinoma (LUAD) is the largest subtype of NSCLC [4]. Radiotherapy combined with chemotherapy is the standard therapy for unresectable locally advanced NSCLC [3]. Unfortunately, resistance to radiotherapy significantly limits its efficacy [5]. Thus, it is of great importance to study the mechanisms underlying lung cancer radioresistance.

N^6^-Methyladenosine (m^6^A) is the most common form of mRNA modification in eukaryotic cells [6]. With effects on mRNA stability, translation, splicing, phase separation, and nuclear export, m^6^A modification is a significant regulatory factor in diverse vital bioprocesses and diseases, including cancer [7]. The reversible and dynamic process of m^6^A modification is facilitated by three functional components: methyltransferases (“writers”), demethylases (“erasers”), and m^6^A-binding proteins (“readers”) [8]. While “writers” and “erasers” are key to controlling the level of m^6^A modification, “readers” are the major regulatory component that affects the fate of mRNAs [7]. Among m^6^A “readers”, IGF2BPs (including IGF2BP1/2/3) recognize m^6^A modifications in mRNAs and promote their stability and translation [9]. As IGF2BPs can bind and stabilize the m^6^A-modified mRNAs of oncoproteins, IGF2BPs have been characterized as oncogenes [9, 10] and are highly expressed in many types of cancer, including lung cancer [11]. Despite extensive studies into the effects of m^6^A “readers” in tumor proliferation [12, 13], progression [14–16], and metastasis [17–19], the role of m^6^A “readers” including IGF2BPs in lung cancer radioresistance remains to be elucidated.

SLC7A5 (solute carrier family 7 member 5 or LAT1) is a sodium-independent transporter mediating the transport of large neutral amino acids across the cell membrane [20]. SLC7A5 is highly expressed in various types of cancer, including NSCLC, and is correlated with poor prognosis in NSCLC patients [21]. SLC7A5 expression supports cancer progression, mostly by supplying essential amino acids to cancer cells, resulting in the activation of the mammalian target of rapamycin (mTOR) pathway to promote tumor proliferation [22]. However, little is known about the role of SLC7A5 in lung cancer radioresistance.

In the present study, we found that the expression of IGF2BP2 is upregulated in radioresistant lung cancer cells. We demonstrated that IGF2BP2 promotes lung cancer radioresistance by forming a positive feedback loop with SLC7A5 to activate the AKT/mTOR pathway. Mechanistically, IGF2BP2 enhances the stability of *SLC7A5* mRNA to promote SLC7A5 expression, which in turn transports methionine (Met) into cells. Met is required for the formation of S-adenosylmethionine (SAM), which is utilized by a histone lysine methyltransferase SETD1A to catalyze the trimethyl modification at lysine 4 of histone H3 (H3K4me3) at the promoter of the *IGF2BP2* gene, thereby enhancing *IGF2BP2* transcription. We also identified a novel IGF2BP2 ubiquitin ligase, FBW7, which could recognize and degrade IGF2BP2, after phosphorylation by GSK3β. Taken together, our study delineated a previously unknown mechanism of a positive feedback loop between IGF2BP2 and SLC7A5, and highlighted the role of the FBW7/GSK3β/IGF2BP2/SLC7A5 axis in radioresistance in lung cancer.

## Methods

### Cell lines and cell culture

Human lung adenocarcinoma cells including NCI-H1299 (H1299) and A549 were purchased from Procell (Wuhan, China). Radioresistant H1299-RR cells were constructed in our laboratory as previously reported [23]. All cell lines were authenticated by short tandem repeat (STR) profiling and tested for mycoplasma contamination. H1299 and H1299-RR cells were cultured in RPMI-1640 medium (Gibco, USA) supplemented with 10% fetal bovine serum (FBS) (#164,210–50, Procell, China) and 1% penicillin/streptomycin (P/S) (#BL505A, Biosharp, China). A549 cells were cultured in DMEM/F12 medium (Gibco, USA) supplemented with 10% FBS and 1% P/S. HEK293T cells were cultured in DMEM medium (Gibco, USA) supplemented with 10% FBS and 1% P/S. All cells were placed in the incubator at 37 ℃ in 5% CO_2_.

### Antibodies and reagents

Anti-IGF2BP2 (#11,601–1-AP, Proteintech, 1:2000), anti-FBW7 (#28,424–1-AP, Proteintech, 1:2000), anti-SLC7A5 (#28,670–1-AP, Proteintech, 1:2000), anti-GSK3β (#22,104–1-AP, Proteintech, 1:1000), anti-H3K4me3 (#A22225, ABclonal, 1:10,000), anti-SETD1A (#A18231, ABclonal, 1:500), anti-mTOR (#66,888–1-Ig, Proteintech, 1:5000), anti-pmTOR-S2448 (#67,778–1-Ig, Proteintech, 1:2000), anti-AKT (#60,203–2-Ig, Proteintech, 1:5000), anti-pAKT-S473 (#66,444–1-Ig, Proteintech, 1:5000), anti-Myc-tag (#60,003–2-Ig, Proteintech, 1:2000), anti-Flag-tag (#66,008–4-Ig, Proteintech, 1:5000), anti-HA-tag (#66,006–2-Ig, Proteintech, 1:10,000), and anti-GAPDH (#60,004–1-Ig, Proteintech, 1:50,000) were used for Western blotting analyses. Anti-SETD1A (#61,702, Cell Signaling Technology, 1:100), anti-H3K4me3 (#9751, Cell Signaling Technology, 1:50), and anti-rabbit IgG (#3900S, Cell Signaling Technology, 1:1000) were used for ChIP assay. Anti-IGF2BP2 (#ab128175, Abcam), anti-m^6^A (#ab151230, Abcam), anti-rabbit IgG (#3900S, Cell Signaling Technology), and anti-mouse IgG (#61656S, Cell Signaling Technology) were used for RIP and MeRIP assays. Anti-IGF2BP2 (#11,601–1-AP, Proteintech, 1:100), anti-FBW7 (#28,424–1-AP, Proteintech, 1:100), and anti-SLC7A5 (#28,670–1-AP, Proteintech, 1:100) were used for immunohistochemistry staining of LUAD tissue microarray. Anti-Rad51 (#8875, Cell Signaling Technology) was used for Rad51 foci formation assay. Actinomycin D (#S8964), 3-deazaadenosine (#S6835), MG132 (#S2619), Cycloheximide (#S7418), TWS119 (#S1590) were purchased from Selleck (Shanghai, China).

### Western blotting analysis and co-immunoprecipitation (co-IP)

Cells were lysed for 20–30 min using RIPA lysis buffer (#G2002, Servicebio, China) on ice with 1% protease inhibitors (#G2008, Servicebio, China) and 1% phosphatase inhibitors (#G2007, Servicebio, China). The lysates were centrifuged at 12,000 rpm at 4 ℃ for 20 min. The supernatants were collected to measure protein concentration using BCA protein analysis kit (#G2026, Servicebio, China). The supernatants were boiled at 100 ℃ for 10 min after adding 5 × loading buffer. The same amount of protein was resolved by sodium dodecyl-sulfate polyacrylamide gel electrophoresis (SDS-PAGE), and then transferred to polyvinylidene fluoride (PVDF) membranes. After blocking by 5% non-fat milk at room temperature for 1 h, the membranes were incubated in the corresponding primary antibodies at 4 ℃ overnight. Then the membranes were washed with 1 × TBST, incubated with secondary antibodies at room temperature for 1 h, and washed with 1 × TBST. The signals were obtained using ECL reagents (#G2020, Servicebio, China) in dark room. For co-immunoprecipitation (co-IP), cells were lysed with Western/IP lysis buffer (#P0013, Beyotime, China) adding 1% protease inhibitors and 1% phosphatase inhibitors on ice for 20–30 min. After centrifugation, the supernatant was collected and then incubated with Protein A/G agarose beads (#G1718, Santa Cruz, USA) and corresponding primary antibodies or IgG at 4 ℃ overnight. The beads were washed 5 times using NETN buffer, and then added with 50 μL 1 × loading buffer, boiled at 100 ℃ for 10 min. The samples were used for Western blotting analyses.

### Plasmids and siRNA transfection and shRNA infection

Myc-IGF2BP2, Flag-SLC7A5, Flag-SETD1A, Flag-FBW7, Flag-GSK3β, HA-Ub were individually cloned into pcDNA3.1 vector by BT Lab (Wuhan, China). Short hairpin RNAs (shRNAs) were purchased from GeneChem (Shanghai, China), siRNAs were purchased from RiboBio (Guangzhou, China). For transfection, lipofectamine 2000 (Thermo Fisher Scientific, USA) and corresponding plasmids or siRNAs were used. For infection, shRNAs together with pMD2.G and psPAX2 were employed to produce lentiviral particles in HEK293T cells. The virus-containing cell culture medium was collected and used to infect LUAD cells in the presence of 10 µg/mL polybrene. Puromycin (2 µg/mL) selection was performed to screen the successfully infected cells for 2 weeks. The sequences of siRNAs and shRNAs are listed in Supplementary Table S1.

### Quantitative real-time PCR (RT-qPCR) assay

RNA was extracted using TRIzol reagent (#R401-01, Vazyme, China). RT-qPCR was performed using a reverse transcription kit (#R323-01, Vazyme, China) and RT-qPCR kit (#Q111-02, Vazyme, China) according to the manufacturer’s instructions. Relative gene expression levels were determined using the 2^−ΔCt^ method after normalizing to GAPDH or β-Actin levels. The sequences of primers are listed in Supplementary Table S2.

### Chromatin immunoprecipitation (ChIP) and ChIP-qPCR assay

The ChIP Assay Kit (#P2078, Beyotime, China) was used according to the manufacturer’s instructions. DNA pulled down by corresponding antibodies was amplified by PCR and was quantified by RT-qPCR using primers located inside or outside the gene promoter region. Data were normalized to the corresponding DNA input control. The sequences of primers are listed in Supplementary Table S3.

### RNA immunoprecipitation (RIP) assay and methylated RNA immunoprecipitation (MeRIP)

RNA Immunoprecipitation Kit (#Bes5101, BersinBio, China) was used according to the manufacturer’s recommendation. Bound RNAs were extracted and subjected to RT-qPCR for quantitative analysis. Relative enrichment was normalized to the input. The sequences of primers for RT-qPCR are listed in Supplementary Table S2.

### mRNA stability assay

Cells were seeded to grow to approximately 50% confluence. Then, Actinomycin D (ActD) (5μg/mL) was used to treat cells. Cells were then harvested at 0 h, 3 h, 6 h, and 9 h. Total RNA was extracted and then analyzed with RT-qPCR. The mRNA value of each group was calculated and normalized to GAPDH at each time point. The degradation rate of mRNA was calculated based on previously published protocol [24].

### RNA pull-down assay

Biotin-labeled *SLC7A5* (full length, 3’-UTR-WT, and 3’-UTR-MUT) probes were purchased from RiboBio (Guangzhou, China). RNA pull-down assay was performed using the RNA Pull-down Kit (#Bes5102, BersinBio, China) by mixing biotinylated RNAs with protein lysates and streptavidin beads. After incubation and washes, the beads were boiled at 100 ℃ and then used for Western blotting analysis.

### Luciferase reporter assay

Wild-type *SLC7A5* 3’UTR and m^6^A sites mutated *SLC7A5* 3’UTR were subcloned into the downstream of pmirGLO firefly luciferase vector by BT Lab (Wuhan, China). Then these plasmids were transfected into different groups of cells. The relative luciferase activity was detected using Dual Luciferase Reporter Gene Assay Kit (#RG027, Beyotime, China). The results were shown as relative firefly luciferase activity normalized to renilla luciferase activity.

### Transcriptome sequencing (RNA-seq)

Total RNA was extracted using TRIzol reagent (#R401-01, Vazyme, China). The extracted RNA was then used for transcriptome sequencing by NOVOGENE (Beijing, China) based on the Illumina platform. The RNA-seq data is provided in Supplementary Table S4.

### Clonogenic survival assay

Different groups of cells were seeded in six-well plates and then irradiated with indicated doses. After 2 weeks, cell colonies were fixed with 4% formaldehyde and stained with crystal violet. Colonies with more than 50 cells were photographed and counted. Survival curves were fitted, and sensitization enhancement ratio (SER) was calculated according to the single-hit multitarget model.

### Annexin V/propidium iodide (PI) assay

Cells treated in different manners were irradiated at 6 Gy. After 48 h, cells were collected using the Annexin V-FITC Apoptosis Detection Kit (#C1062L, Beyotime, China) following the manufacturer’s instructions.

### Neutral comet assay

The reagent kit for neutral comet assay (#4250–050-K, Trevigen, USA) was used according to the manufacturer’s recommendation. Briefly, different groups of cells were irradiated at 6 Gy for 4 h, then collected and mixed with 37 ℃ low-melting point agarose. The mixture was immediately layered onto pretreated slides. Slides were immersed in lysis buffer for 1 h and then placed in 1 × neutral electrophoresis buffer at 21 V for 45 min. After immersing in DNA precipitation solution for 30 min, the slides were stained with SYBR for 10 min and observed by a fluorescent microscope. The olive tail moment of at least 100 cells each group was analyzed using CometScore 2.0.

### Immunofluorescence staining

Cells were treated in different manners and then irradiated at 6 Gy. After 4 h of irradiation, cells were fixed with paraformaldehyde and then blocked with 5% bovine serum albumin. Cells were then incubated with anti-Rad51 and secondary antibody. After washing with PBS for three times, Rad51 foci was observed using a laser confocal microscope. Cells with more than 10 foci were considered positive.

### Mice xenograft model

Animal procedures were approved by the Ethics Committee of Tongji Medical College, Huazhong University of Science and Technology. BALB/c nude mice (4–5 weeks old) were purchased from Moubaili Biotechnology Company (Wuhan, China). Mice were randomly divided into different groups (at least *n* = 5 each group). H1299-RR cells or A549 cells (5 × 10^6^) treated in different manners were harvested and inoculated subcutaneously into the left dorsal flank of mice. On the 7th day, when tumor volumes reached approximately 80–100 mm^3^, mice in irradiation groups were irradiated with 10 Gy. Tumor volumes were calculated as follows: 0.5 × (L × W^2^), where L = length, W = width.

### Immunohistochemistry (IHC) staining and scoring analyses

LUAD tissue microarray (R076Lu01) was purchased from Zhongke Guanghua Intelligent Biological Technology Company (Xi’an, China). The microarray was stained with anti-IGF2BP2, anti-FBW7, and anti-SLC7A5 antibodies using the Immunohistochemistry (IHC) Staining Kit (Bios Biological Technology Company, China). IHC scores were calculated as follows: the staining intensity × the percentage of positive staining cells. The staining intensity was defined as: (0) no staining at 100 × magnification; (1) weak staining at 100 × magnification and little staining at 40 × magnification; (2) moderate staining at 40 × magnification; (3) strong staining at 40 × magnification.

### Methionine (Met) and S-adenosylmethionine (SAM) assay

Cellular Met was assayed by Human Met ELISA kit according to the manufacturer’s instructions (COIBO BIO, China). Briefly, cells were collected and diluted with PBS (pH 7.2–7.4), then repeated with freeze–thaw cycles to damage cells and release intracellular components. The suspension was centrifuged at 2000–3000 rpm for 20 min to collect supernatant. Samples were transferred to the ELISA plate, and then treated with adding reagent, incubating, washing, coloring, and terminating. Within 15 min, the absorbance was measured on EnSpire multimode plate reader (PerkinElmer) at 450 nm.

Cellular SAM was assayed by Bridge-IT® S-adenosylmethionine fluorescence assay kit according to the manufacturer’s instructions (Mediomics, USA). Briefly, cells were lysed in ice-cold 0.2% perchloric acid and 0.08% β-mercaptoethanol, and then combined with assay reagents, incubated at room temperature for 30 min. The mixture was transferred to a black-bottom 96-well plate, and assayed on EnSpire multimode plate reader (PerkinElmer) at 485 nm excitation and 665 nm emission settings.

### Statistical analysis

The replicants for each experiment were indicated in the figure legend. Data were presented as means ± standard deviation (SD). All statistical analyses were performed using GraphPad Prism 9 software. Unpaired t test was used to compare data between two groups. One-way ANOVA followed by Turkey’s multiple comparison test was used to compare data between multiple groups. *P* values < 0.05 were considered statistically significant. In all cases, the significance of differences was indicated as follows: ns, not significant, *P* > 0.05; *, *P* < 0.05; **, *P* < 0.01; ***, *P* < 0.001.

## Results

### IGF2BP2 is overexpressed in radioresistant lung cancer cells and promotes radioresistance in lung cancer

**Fig. 1:**
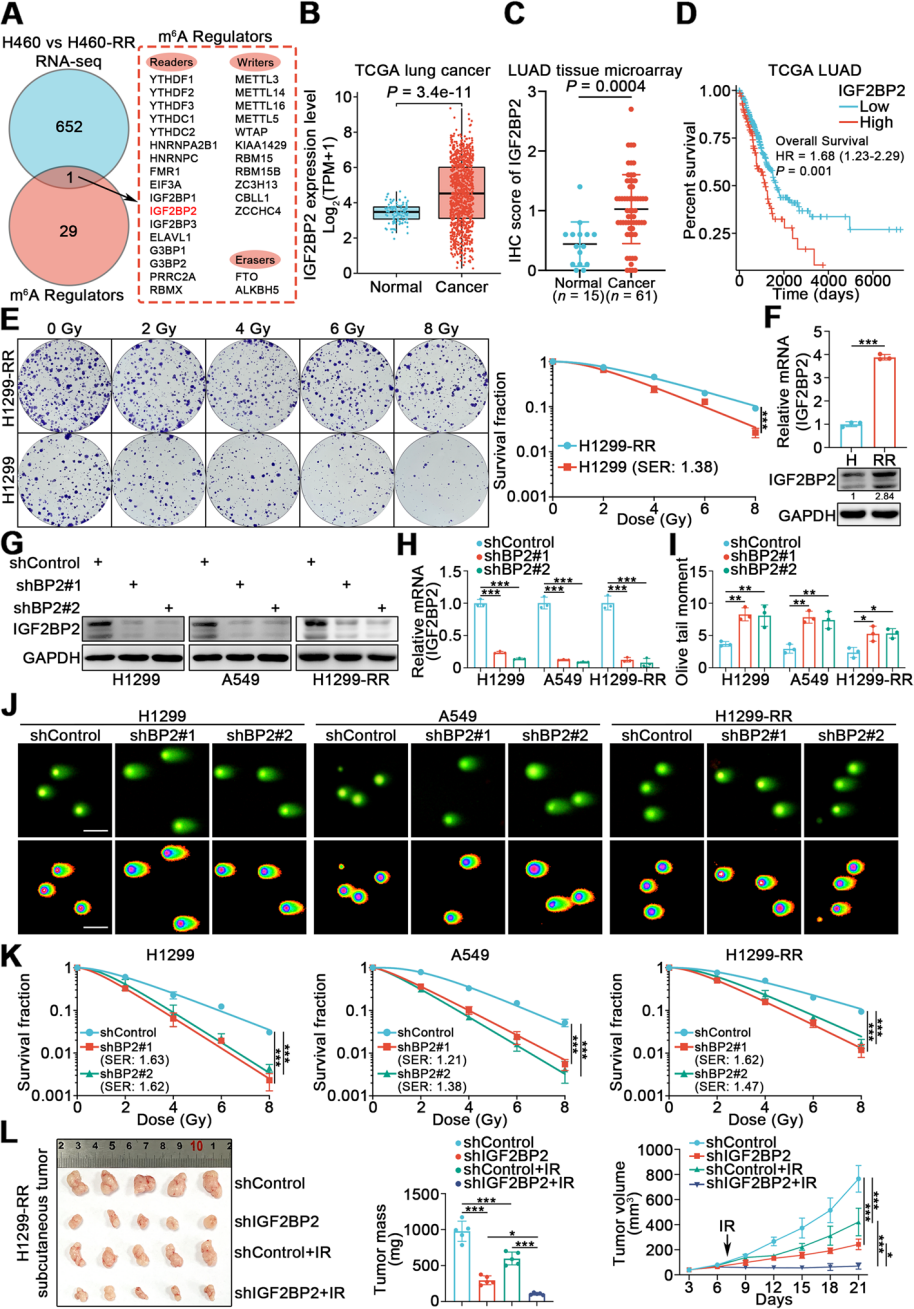
IGF2BP2 is overexpressed in radioresistant lung cancer cells and promotes radioresistance in lung cancer. **A** Venn diagrams showing numbers of differentially expressed genes between H460 and H460-RR cells and genes belonging to m^6^A regulators. **B** Differential expression analysis of IGF2BP2 between tumor and normal tissues in TCGA lung cancer database. **C** Scatter plot showing the IHC score of IGF2BP2 protein in LUAD tissue microarray. Normal *n* = 15, cancer *n* = 61, unpaired t test. Data are presented as Mean ± SD. **D** The overall survival of patients with LUAD with different IGF2BP2 levels based on TCGA LUAD database. **E** H1299 and H1299-RR cells were irradiated at various doses for clonogenic survival assay. SER, sensitization enhancement ratio. *n* = 3, unpaired t test. Data are presented as Mean ± SD. **F** RT-qPCR and Western blotting analyses were performed in H1299 (H) and H1299-RR (RR) cells. *n* = 3, unpaired t test. Data are presented as Mean ± SD. **G-K** H1299, A549, and H1299-RR cells infected with indicated lentivirus vectors, after puromycin selection, were harvested for Western blotting analysis (**G**), RT-qPCR analysis (**H**), neutral comet assay (**I **and** J**), and clonogenic survival assay (**K**). *n* = 3, one-way ANOVA. Data are presented as Mean ± SD. Scale bar, 50 μm. **L** H1299-RR cells were transfected with indicated constructs. After puromycin selection, cells were injected subcutaneously into nude mice. The mice were treated with or without IR (10 Gy). Tumor volumes were measured every 3 days. Tumors were harvested, photographed, and weighted at day 21. *n* = 5, one-way ANOVA. Data are presented as Mean ± SD. *, *P* < 0.05; **, *P* < 0.01; ***, *P* < 0.001. Abbreviations: BP2, IGF2BP2

To identify m^6^A regulators responsible for radiosensitivity in lung cancer, we performed bioinformatics data mining. We first assembled a gene list (*n* = 30) of m^6^A regulators [7], then identified an RNA-seq dataset containing differentially expressed genes (DEGs) between H460 cells and radioresistant H460-RR cells [25]. A total of 653 DEGs were identified with thresholds of |log_2_ fold change|> 2.0 and FDR < 0.05. Combining these two gene sets, we screened one candidate gene, IGF2BP2, belonging to m^6^A “readers” (Fig. 1A). Based on TCGA lung cancer database (Fig. 1B) and LUAD tissue microarray (Fig. 1C and Supplementary Fig. 1A), IGF2BP2 expression was found to be elevated in lung cancer. Moreover, aberrant IGF2BP2 expression was associated with poor overall survival (OS) in lung cancer patients (Fig. 1D). Although it was shown that some tumor samples express lower level of IGF2BP2 than normal samples (Fig. 1B), and survival curves separate moderately (Fig. 1D), the general tendency presented in TCGA lung cancer database analyses aligned with the broadly acknowledged conclusion that IGF2BP2 acts as an oncogene. We then measured the expression of IGF2BP2 in radioresistant H1299 (H1299-RR) cells and their parental H1299 cells. The H1299-RR cells were constructed in our laboratory as previously reported [23], and radioresistance was confirmed by clonogenic survival assay, Rad51 foci formation assay, and neutral comet assay. Compared with the parental line, H1299-RR cells exhibited higher colony formation ability after irradiation (IR) (Fig. 1E), greater Rad51 foci formation indicating increased DNA repair capacity (Supplementary Fig. 1B), and shorter olive tail moment indicating decreased IR-induced DNA damage (Supplementary Fig. 1C). By RT-qPCR and Western blotting analysis, we demonstrated that IGF2BP2 expression was higher in H1299-RR cells compared to H1299 cells (Fig. 1F).

Given the elevated expression of IGF2BP2 in radioresistant lung cancer cells, we further explored the specific role of IGF2BP2 in lung cancer radiosensitivity. We silenced IGF2BP2 expression in H1299, A549, and H1299-RR cells (Fig. 1G, H). Neutral comet assay, Annexin V/propidium iodide (PI) assay, and Rad51 foci formation assay showed that IGF2BP2 knockdown increased IR-induced DNA damage and apoptosis rate, while impairing DNA damage repair (Fig. 1I, J and Supplementary Fig. 1D-F). Consistently, the clonogenic survival assay showed that IGF2BP2 silencing sensitized lung cancer cells to IR (Fig. 1K). To further confirm these findings in vivo, we established a subcutaneous xenograft tumor model in nude mice. We found that the inhibition of lung cancer tumor growth by IR was significantly enhanced when IGF2BP2 was silenced (Fig. 1L). In contrast, the overexpression of IGF2BP2 impaired the ability of IR to inhibit tumor growth (Supplementary Fig. 1G, H). Moreover, the IGF2BP2 protein level before and post-IR was compared in both H1299 and H1299-RR cells. We found that IR did not affect IGF2BP2 protein level, indicating that the overexpression of IGF2BP2 in radioresistant lung cancer cells may not result from IR (Supplementary Fig. 1I). Therefore, these results demonstrate that the aberrant expression of IGF2BP2 promotes radioresistance in lung cancer.

### IGF2BP2 enhances *SLC7A5* mRNA stability through an m^6^A-dependent mechanism in lung cancer cells

**Fig. 2:**
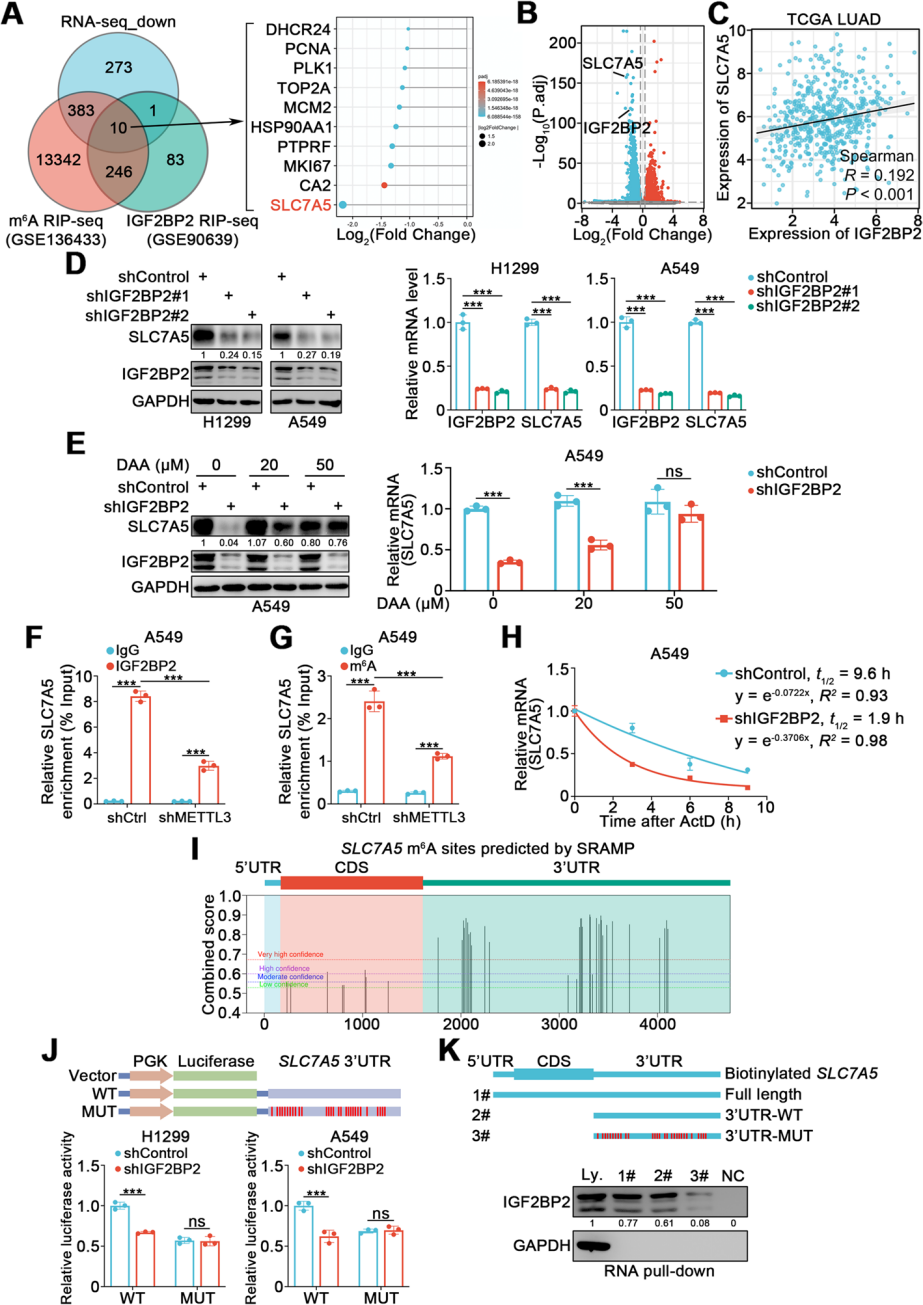
IGF2BP2 enhances *SLC7A5* mRNA stability through an m^6^A-dependent mechanism in lung cancer cells. **A** Left panel, Venn diagrams showing numbers of down-regulated genes in RNA-seq and target genes in GSE136433 and GSE90639 datasets. Right panel, the bar chart showing the overlapped 10 genes ranking according to Log_2_(Fold Change) value. **B** Volcano plot showing the differentially expressed genes from RNA-seq. **C** The expression of IGF2BP2 was positively correlated with SLC7A5 in TCGA LUAD database. **D** H1299 and A549 cells were infected with indicated lentivirus. After puromycin selection, cells were harvested for Western blotting and RT-qPCR analyses. *n* = 3, one-way ANOVA. Data are presented as Mean ± SD. **E** A549 cells were infected with indicated lentivirus. After puromycin selection, cells were treated with different concentrations of DAA (0, 20, and 50 μM), and were harvested for Western blotting and RT-qPCR analyses. *n* = 3, one-way ANOVA. Data are presented as Mean ± SD. **F **and** G** RIP-qPCR using IGF2BP2 antibody and MeRIP-qPCR analyses in A549 cells infected with shControl or shMETTL3. *n* = 3, one-way ANOVA. Data are presented as Mean ± SD. **H** mRNA stability assay in A549 cells infected with shControl and shIGF2BP2. *n* = 3. Data are presented as Mean ± SD. **I** The predicted m^6^A sites in *SLC7A5* mRNA by SRAMP tool. CDS, coding sequence. **J** Luciferase reporter assay using luciferase reporters expressing wild-type (WT) or mutant (MUT) *SLC7A5* 3’UTRs in H1299 and A549 cells infected with shControl or shIGF2BP2. *n* = 3, unpaired t test. Data are presented as Mean ± SD. **K** Western blotting analysis of IGF2BP2 after RNA pull-down assay with cell lysate (Ly.), full length biotinylated *SLC7A5* (1#), the *SLC7A5* 3’UTR region without or with m^6^A sites mutation (2#, 3#), and beads only (NC) in A549 cells. ns, not significant, *P* > 0.05; **, *P* < 0.01; ***, *P* < 0.001

To further elucidate the downstream mechanism underlying the regulation of radiosensitivity by IGF2BP2, we performed RNA-seq to explore the transcriptome profiles of H1299 cells transfected with a small interfering RNA (siRNA) against IGF2BP2. Since IGF2BP2 has been identified as an m^6^A “reader” that promotes the stability of its target mRNAs [9], we combined our RNA-seq dataset (down-regulated genes) with an IGF2BP2 RIP-seq dataset (GSE90639) and an m^6^A RIP-seq dataset (GSE136433). A total of 10 DEGs were screened, among which SLC7A5 was the most downregulated following IGF2BP2 silencing (Fig. 2A, B). Consistently, in the TCGA LUAD dataset, there is a moderate tendency that the expression of IGF2BP2 was positively correlated with that of SLC7A5 (Fig. 2C). Furthermore, in both H1299 and A549 cells, we found that knockdown of IGF2BP2 resulted in decreased SLC7A5 protein and mRNA levels (Fig. 2D); conversely, IGF2BP2 overexpression increased SLC7A5 protein and mRNA levels (Supplementary Fig. 2A). More importantly, the regulatory effect of SLC7A5 by IGF2BP2 could be dose-dependently inhibited by the global methylation inhibitor 3-deazaadenosine (DAA), indicating that the effect of IGF2BP2 on SLC7A5 expression was dependent on methylation status (Fig. 2E and Supplementary Fig. 2B). We then aimed to verify whether IGF2BP2 acted as an m^6^A “reader” that bound to and stabilized *SLC7A5* mRNA in an m^6^A-dependent manner. RIP-qPCR using anti-IGF2BP2 antibody showed a direct interaction between IGF2BP2 and *SLC7A5* mRNA (Fig. 2F). MeRIP-qPCR assay indicated that the m^6^A level of *SLC7A5* mRNA was decreased after the m^6^A “writer” METTL3 knockdown (Fig. 2G). Importantly, the interaction between IGF2BP2 and *SLC7A5* mRNA was impaired after METTL3 knockdown, indicating that this interaction was dependent on the m^6^A level (Fig. 2F). Moreover, mRNA stability assay revealed that the half-life of *SLC7A5* mRNA was shortened upon IGF2BP2 knockdown (Fig. 2H).

To predict the specific m^6^A sites in *SLC7A5* mRNA, the SRAMP tool (https://www.cuilab.cn/sramp) [26] was used. A total of 29 m^6^A sites were predicted to have very high confidence, and all were located in the 3’UTR region (Fig. 2I). Next, we validated these putative m^6^A sites through luciferase reporter assays and RNA pull-down assays. Luciferase activity was significantly attenuated upon IGF2BP2 knockdown in wild-type (WT) reporter cells, but not in cells with a mutant reporter (MUT) that had mutated m^6^A sites (Fig. 2J). RNA pull-down assays verified that IGF2BP2 predominantly bound to the 3’UTR region of *SLC7A5* mRNA, and that the binding was significantly impaired when m^6^A sites were mutated (Fig. 2K). These results demonstrate that IGF2BP2 binds to the 3’UTR region of *SLC7A5* mRNA and enhances its stability in an m^6^A-dependent manner, to increase SLC7A5 expression in lung cancer cells.

### SLC7A5 transports Met into cells and increases H3K4me3 enrichment to promote IGF2BP2 expression in lung cancer cells

**Fig. 3:**
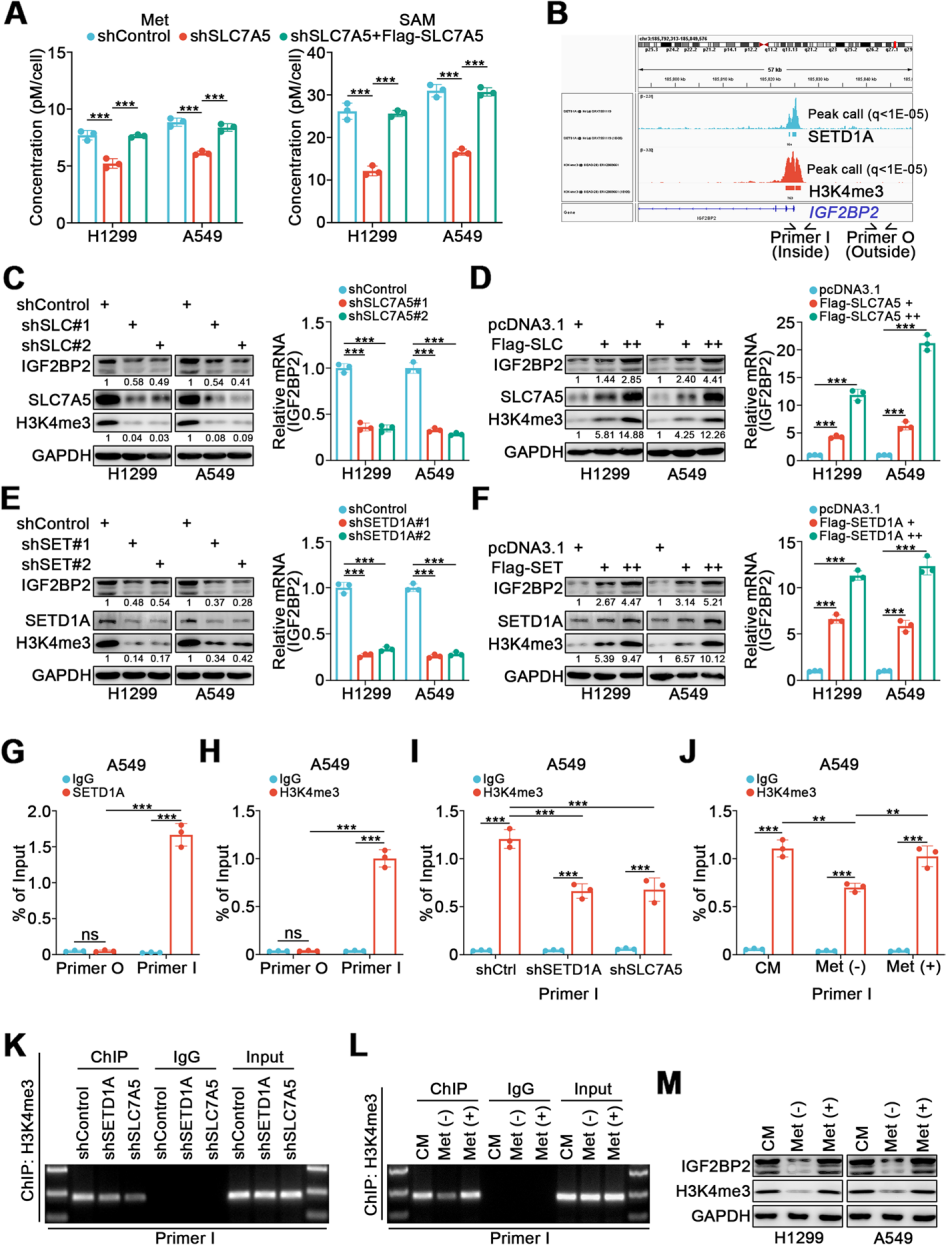
SLC7A5 transports Met into cells and increases H3K4me3 enrichment to promote IGF2BP2 expression in lung cancer cells. **A** H1299 and A549 cells were infected with indicated lentivirus. After puromycin selection, cells were collected for methionine (Met) and S-adenosylmethionine (SAM) assays. *n* = 3, one-way ANOVA. Data are presented as Mean ± SD. **B** The ChIP-seq of SETD1A and H3K4me3 on the promoter region of *IGF2BP2*. **C-F** H1299 and A549 cells were infected with indicated lentivirus. After puromycin selection, cells were harvested for Western blotting and RT-qPCR analyses. *n* = 3, one-way ANOVA. Data are presented as Mean ± SD. **G **and** H** ChIP-qPCR analyses using SETD1A (**G**) or H3K4me3 (**H**) antibodies in A549 cells. *n* = 3, one-way ANOVA. Data are presented as Mean ± SD. **I-L** A549 cells were infected with shControl, shSETD1A, shSLC7A5, or treated with complete medium (CM), Met deprivation [Met (-)], or Met restoration [Met ( +)]. Cells were harvested for ChIP-qPCR analyses using H3K4me3 antibody (**I **and** J**). And the images for agarose electrophoresis of the products from the corresponding ChIP assay (**K **and** L**). *n* = 3, one-way ANOVA. Data are presented as Mean ± SD. **M** H1299 and A549 cells were treated with complete medium (CM), Met deprivation [Met (-)], or Met restoration [Met ( +)]. Cells were harvested for Western blotting analysis. ns, not significant, *P* > 0.05; **, *P* < 0.01; ***, *P* < 0.001. Abbreviations: SLC, SLC7A5; SET, SETD1A

SLC7A5 is a transporter mediating the import of large neutral amino acids into cells, including Met [20]. Methionine adenosyl transferase catalyzes the conversion of Met into SAM, a universal methyl donor which can participate in epigenetic regulation. Thus, we speculated that SLC7A5 may influence epigenetics by controlling Met influx. We first observed that SLC7A5 silencing resulted in a significant reduction of both Met and SAM concentrations, whereas Met and SAM levels were restored by SLC7A5 overexpression (Fig. 3A). ChIP-Atlas analysis identified a SETD1A binding peak and a H3K4me3 binding peak overlapping at the promoter region of *IGF2BP2* (Fig. 3B). SETD1A is a component of a histone methyltransferase complex that produces mono-, di-, and trimethylated histone H3 at lysine 4, and H3K4me3 is known to mark the transcription start sites in active genes; hence, we speculated that SLC7A5 may increase IGF2BP2 expression through enhancing the H3K4me3 level at the *IGF2BP2* promoter region in conjunction with SETD1A. As expected, the protein and mRNA levels of IGF2BP2 were decreased by either SLC7A5 or SETD1A knockdown, and increased by SLC7A5 or SETD1A overexpression. The same trends were observed in H3K4me3 levels (Fig. 3C-F). ChIP-qPCR assays confirmed the presence of SETD1A and H3K4me3 in the *IGF2BP2* promoter region (Fig. 3G, H). In addition, knockdown of SETD1A or SLC7A5 significantly reduced H3K4me3 enrichment in the *IGF2BP2* promoter region (Fig. 3I, K). Moreover, H3K4me3 enrichment in the *IGF2BP2* promoter region significantly decreased after Met deprivation, but was rescued by Met supplementation (Fig. 3J, L). The protein level of IGF2BP2 exhibited the same trends as H3K4me3 in response to changes in Met availability (Fig. 3M). These results demonstrate that SLC7A5 mediates the influx of Met, which is converted to SAM to produce H3K4me3 at the *IGF2BP2* promoter region, thus promoting IGF2BP2 expression in lung cancer cells.

### The IGF2BP2-SLC7A5 positive feedback loop promotes radioresistance in lung cancer through the AKT/mTOR pathway

**Fig. 4:**
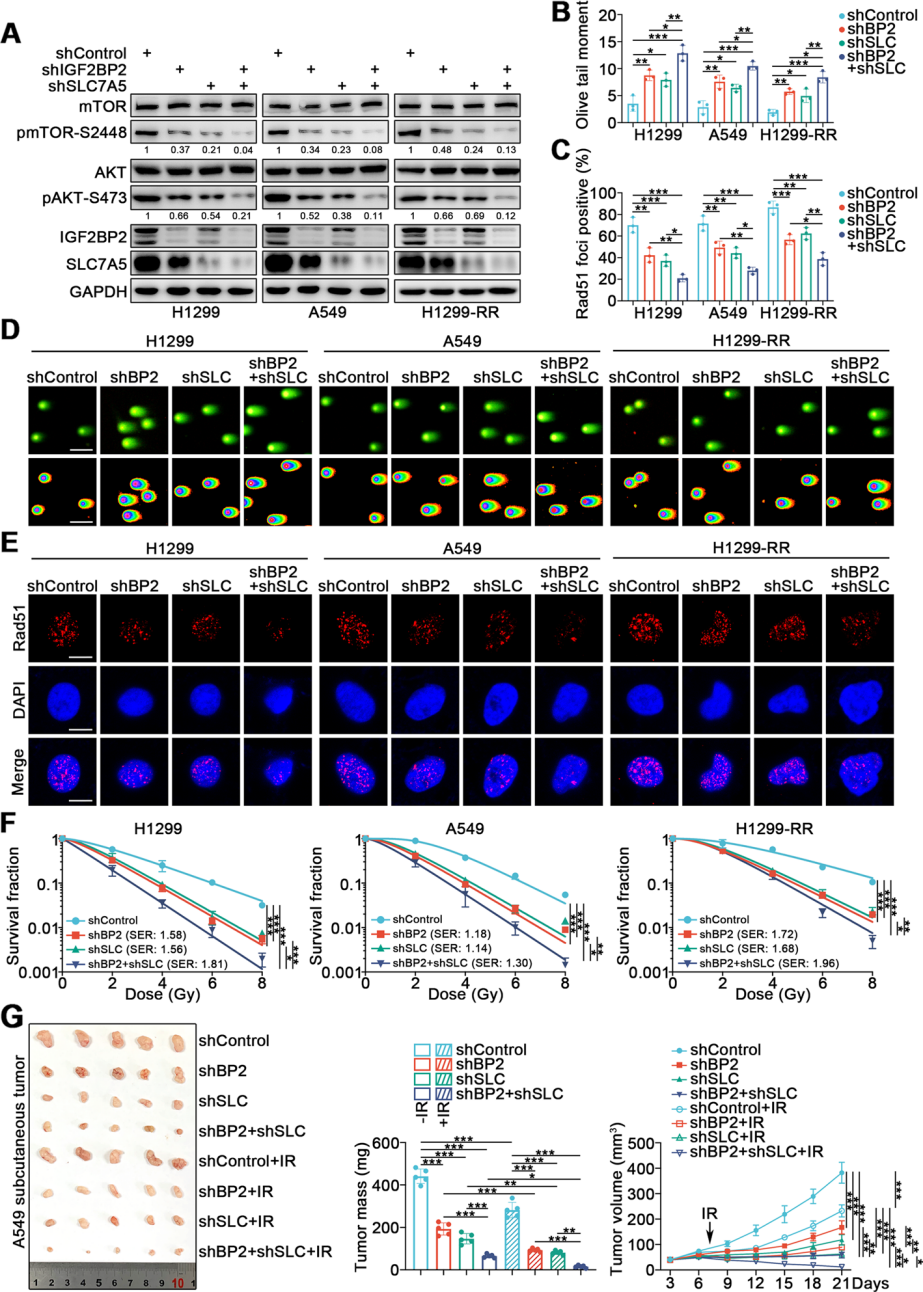
The IGF2BP2-SLC7A5 positive feedback loop promotes radioresistance in lung cancer through the AKT/mTOR pathway. **A** H1299, A549, and H1299-RR cells were infected with indicated lentivirus. After puromycin selection, cells were harvested for Western blotting analysis. **B-E** H1299, A549, and H1299-RR cells were infected with indicated lentivirus. After puromycin selection, cells were irradiated at 6 Gy. After 4 h, cells were harvested for neutral comet assay (**B **and** D**) and Rad51 foci formation assay (**C **and** E**). *n* = 3, one-way ANOVA. Data are presented as Mean ± SD. Scale bars, 50 μm in **D** 10 μm in **E**. **F** H1299, A549, and H1299-RR cells were infected with indicated lentivirus. After puromycin selection, cells were irradiated at various doses for clonogenic survival assay. *n* = 3, one-way ANOVA. Data are presented as Mean ± SD. **G** A549 cells were transfected with indicated constructs. After puromycin selection, cells were injected subcutaneously into nude mice. The mice were treated with or without IR (10 Gy). Tumor volumes were measured every 3 days. Tumors were harvested, photographed, and weighted at day 21. *n* = 5, one-way ANOVA. Data are presented as Mean ± SD. *, *P* < 0.05; **, *P* < 0.01; ***, *P* < 0.001. Abbreviations: BP2, IGF2BP2; SLC, SLC7A5

The above results depicted a positive feedback loop between IGF2BP2 and SLC7A5, which could be validated by LUAD tissue microarray as the expression levels of IGF2BP2 and SLC7A5 were positively correlated (Supplementary Fig. 3A-C). We next assessed whether this loop participated in lung cancer radioresistance. Since SLC7A5 has been widely reported to activate the AKT/mTOR pathway [22], and the AKT/mTOR pathway has been found to regulate reactive oxygen species and protect cells from DNA damage [27], we speculated that the IGF2BP2-SLC7A5 loop may confer radioresistance on lung cancer through the AKT/mTOR pathway. As expected, IGF2BP2 silencing resulted in SLC7A5 downregulation and reduction of mTOR and AKT phosphorylation levels. Similarly, SLC7A5 silencing resulted in the downregulation of IGF2BP2 and decreased mTOR and AKT phosphorylation. Simultaneous silencing of IGF2BP2 and SLC7A5 resulted in the greatest reduction in IGF2BP2 and SLC7A5 expression, and mTOR and AKT phosphorylation (Fig. 4A). Accordingly, neutral comet assay, Annexin V/PI assay, Rad51 foci formation assay, and clonogenic survival assay showed that either IGF2BP2 or SLC7A5 knockdown was sufficient to induce an increase in IR-induced DNA damage and apoptosis rate, concurrent with impaired DNA damage repair and survival. Silencing of both IGF2BP2 and SLC7A5 showed additive effects on these phenotypes (Fig. 4B-F and Supplementary Fig. 3D). In an in vivo subcutaneous tumor model, knockdown of IGF2BP2 or SLC7A5 inhibited tumor growth and enhanced the antitumor effects of IR, while co-knockdown of IGF2BP2 and SLC7A5 had the strongest anti-tumor effects (Fig. 4G). These results demonstrate that the positive feedback loop between IGF2BP2 and SLC7A5 conferred lung cancer radioresistance by activating the AKT/mTOR pathway.

### FBW7 negatively regulates the stability of IGF2BP2 protein by ubiquitinating IGF2BP2 in lung cancer cells

**Fig. 5:**
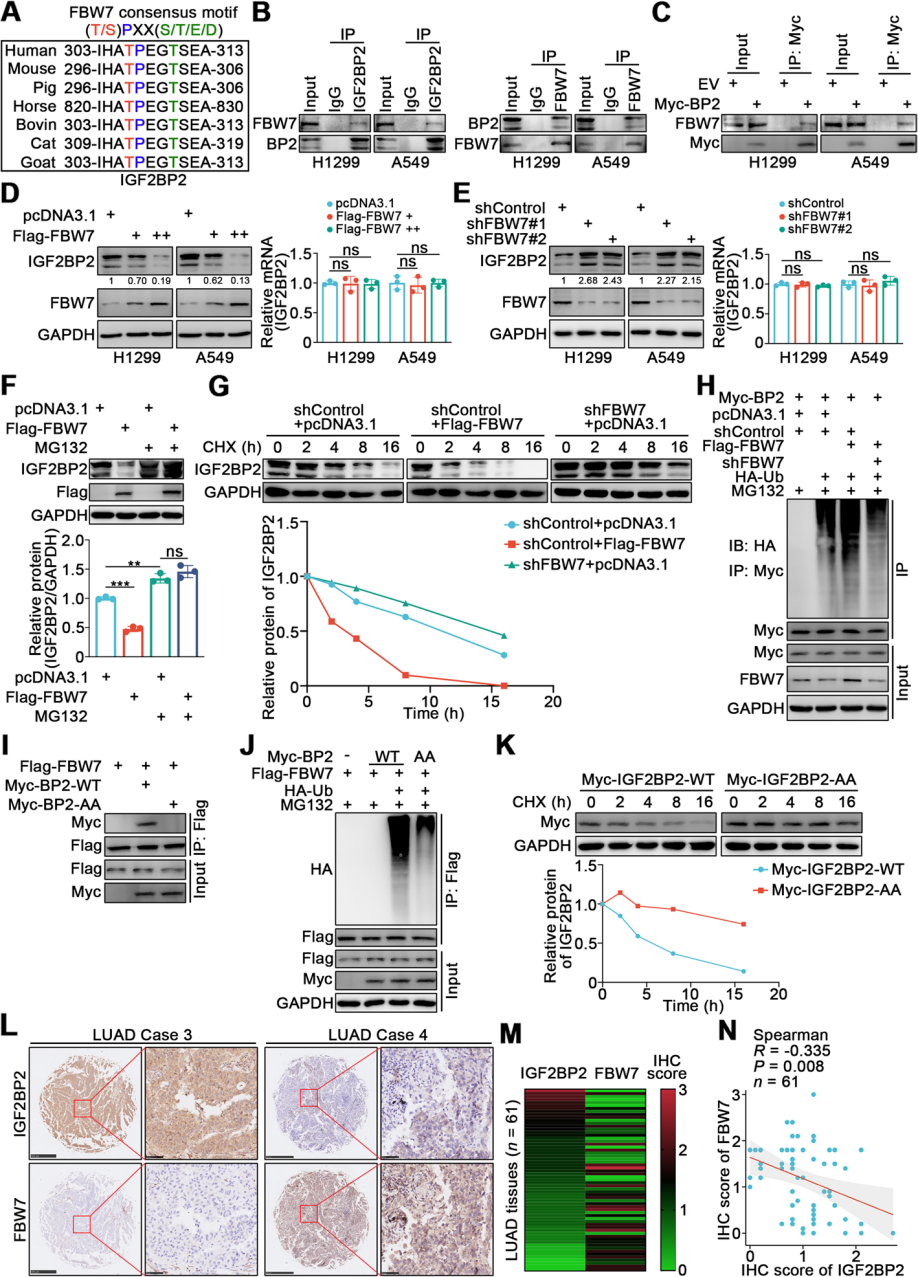
FBW7 negatively regulates the stability of IGF2BP2 protein by ubiquitinating IGF2BP2 in lung cancer cells. **A** A schematic diagram depicting FBW7 consensus motif in IGF2BP2 conserved region. **B** H1299 and A549 cells were harvested and immunoprecipitated with IgG, IGF2BP2, or FBW7 antibodies. **C** H1299 and A549 cells were transfected with EV or Myc-IGF2BP2 plasmids for 72 h. Cells were collected and immunoprecipitated with Myc antibody. **D **and** E** H1299 and A549 cells were infected with indicated lentivirus. After puromycin selection, cells were harvested for Western blotting and RT-qPCR analyses. *n* = 3, one-way ANOVA. Data are presented as Mean ± SD. **F** A549 cells were infected with indicated lentivirus. After puromycin selection, the corresponding groups were treated with MG132 for another 12 h. Cells were harvested for Western blotting analysis. *n* = 3, one-way ANOVA. Data are presented as Mean ± SD. **G,** A549 cells were infected with indicated lentivirus. After puromycin selection, cells were treated with Cycloheximide (CHX) and then collected at different time points for Western blotting analysis to detect IGF2BP2 levels. **H** A549 cells were transfected with indicated plasmids (Myc-IGF2BP2, Flag-FBW7, shFBW7, HA-Ub) for 48 h. Then cells were treated with MG132 for another 12 h. Cells were harvested for Western blotting analysis. **I** A549 cells were transfected with Flag-FBW7, plasmid expressing wild-type IGF2BP2 (Myc-IGF2BP2-WT), and plasmid expressing IGF2BP2 with Thr306 and Thr310 residues mutated to alanine (Myc-IGF2BP2-AA) for 72h. Cells were collected and immunoprecipitated with Flag antibody for Western blotting analysis. **J** A549 cells were transfected with indicated plasmids (Myc-IGF2BP2-WT, Myc-IGF2BP2-AA, Flag-FBW7, HA-Ub) for 48 h. Then cells were treated with MG132 for another 12 h. Cells were harvested for Western blotting analysis. **K** A549 cells were transfected with Myc-IGF2BP2-WT or Myc-IGF2BP2-AA for 72 h. Then cells were treated with Cycloheximide (CHX) and then collected at different time points for Western blotting analysis to detect IGF2BP2 levels. **L-N** The LUAD tissue microarray (*n* = 61) was stained with IGF2BP2 and FBW7, respectively. The representative images of IHC for IGF2BP2 and SLC7A5 protein were shown in panel (**L**). Scale bars, 500 μm for low power field, 50 μm for high power field. The heatmap showing IHC score of IGF2BP2 and SLC7A5 protein was shown in panel (**M**). The correlation analysis of IHC score among IGF2BP2 and SLC7A5 was shown in panel (**N**). ns, not significant, *P* > 0.05; **, *P* < 0.01; ***, *P* < 0.001. Abbreviations: BP2, IGF2BP2

We next analyzed the regulation of IGF2BP2 in lung cancer. We found a unique overlap between the conserved region of the IGF2BP2 gene and the consensus motif recognized by FBW7 (Fig. 5A). Since FBW7 is an E3 ubiquitin ligase, we speculated that IGF2BP2 may be regulated by FBW7 through the ubiquitin–proteasome pathway. Indeed, a co-IP assay demonstrated that IGF2BP2 interacted with FBW7 (Fig. 5B, C). Overexpression or knockdown of FBW7 resulted in decreased or increased IGF2BP2 protein levels, respectively, while there were no significant changes at the mRNA level (Fig. 5D, E). Moreover, the FBW7-induced changes in IGF2BP2 protein levels were inhibited by a proteasome inhibitor, MG132 (Fig. 5F). Furthermore, the half-life of IGF2BP2 protein was shortened after FBW7 overexpression, while FBW7 knockdown exhibited the opposite effect (Fig. 5G). We also found that FBW7 overexpression increased the polyubiquitination of IGF2BP2, whereas FBW7 knockdown decreased the polyubiquitination of IGF2BP2 (Fig. 5H). To further investigate whether the conserved TPXXT sites in IGF2BP2 were associated with binding and ubiquitination, we constructed an IGF2BP2 mutant with threonine-to-alanine mutations at Thr306 and Thr310; this mutant was called IGF2BP2-AA. Unlike IGF2BP2-WT, IGF2BP2-AA failed to bind with FBW7 (Fig. 5I), and its polyubiquitination by FBW7 was not obvious (Fig. 5J). Meanwhile, the half-life of IGF2BP2-AA was remarkably longer than that of IGF2BP2-WT (Fig. 5K). Moreover, as expected, the expression levels of FBW7 and IGF2BP2 showed a significant negative correlation in LUAD tissue microarray (Fig. 5L-N). Hence, these results demonstrate that FBW7 negatively regulates the stability of IGF2BP2 protein by ubiquitinating IGF2BP2 in lung cancer cells.

### GSK3β promotes the binding and ubiquitination of IGF2BP2 by FBW7 in lung cancer cells

**Fig. 6:**
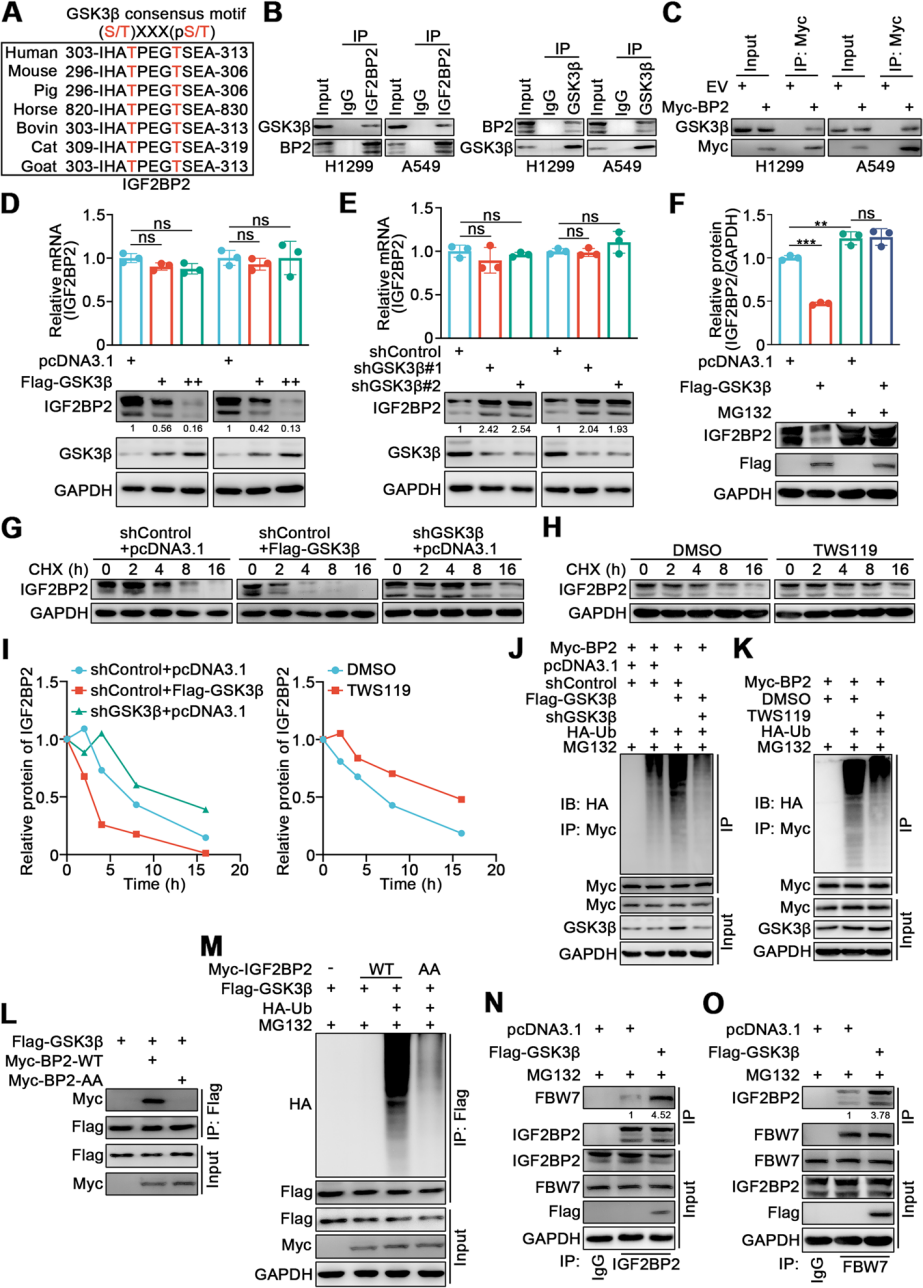
GSK3β promotes the binding and ubiquitination of IGF2BP2 by FBW7 in lung cancer cells. **A** A schematic diagram depicting GSK3β consensus motif in IGF2BP2 conserved region. **B** H1299 and A549 cells were harvested and immunoprecipitated with IgG, IGF2BP2, or GSK3β antibodies. **C** H1299 and A549 cells were transfected with EV or Myc-IGF2BP2 plasmids for 72 h. Cells were collected and immunoprecipitated with Myc antibody. **D** and** E** H1299 and A549 cells were transfected with indicated plasmids. After 48 h and 72 h, cells were harvested for RT-qPCR and Western blotting analyses. *n* = 3, one-way ANOVA. Data are presented as Mean ± SD. **F** A549 cells were transfected with Flag-GSK3β. After 48 h, the corresponding groups were treated with MG132 for another 12 h. Cells were harvested for Western blotting analysis. *n* = 3, one-way ANOVA. Data are presented as Mean ± SD. **G-I** A549 cells were transfected with indicated plasmids for 72 h or treated with GSK3β specific inhibitor TWS119 (10 μM) for 1 h. Cells were treated with Cycloheximide (CHX) and then collected at different time points for Western blotting analysis to detect IGF2BP2 levels. **J** and** K** A549 cells were transfected with indicated plasmids (Myc-IGF2BP2, Flag-GSK3β, shGSK3β, HA-Ub) for 48 h or treated with TWS119 (10 μM) for 1 h. Then cells were treated with MG132 for another 12 h. Cells were harvested for Western blotting analysis. **L** A549 cells were transfected with Flag-GSK3β, plasmid expressing wild-type IGF2BP2 (Myc-IGF2BP2-WT), and plasmid expressing IGF2BP2 with Thr306 and Thr310 residues mutated to alanine (Myc-IGF2BP2-AA) for 72h. Cells were collected and immunoprecipitated with Flag antibody for Western blotting analysis. **M** A549 cells were transfected with indicated plasmids (Myc-IGF2BP2-WT, Myc-IGF2BP2-AA, Flag-GSK3β, HA-Ub) for 48 h. Then cells were treated with MG132 for another 12 h. Cells were harvested for Western blotting analysis. **N **and** O** A549 cells were transfected with Flag-GSK3β for 48 h. Then cells were treated with MG132 for another 12 h. Cells were collected and immunoprecipitated with IgG, IGF2BP2, or FBW7 antibodies for Western blotting analyses. ns, not significant, *P* > 0.05; **, *P* < 0.01; ***, *P* < 0.001. Abbreviations: BP2, IGF2BP2

Since it has been reported that FBW7 binds to its substrates after they have been phosphorylated in the consensus motif, and substrates are mostly phosphorylated by GSK3β [28], we next investigated whether GSK3β participated in the FBW7-mediated binding and ubiquitination of IGF2BP2. We found that the consensus motif for GSK3β overlapped with the IGF2BP2 consensus motif (Fig. 6A), and co-IP assay confirmed an interaction between IGF2BP2 and GSK3β (Fig. 6B, C). IGF2BP2 protein levels were decreased after GSK3β overexpression and increased after GSK3β knockdown, while the mRNA level remained unchanged (Fig. 6D, E). Furthermore, the GSK3β-induced decrease in IGF2BP2 protein levels could be rescued by MG132 (Fig. 6F). Consistent with a proteasome-dependent effect, the half-life of IGF2BP2 protein was decreased by GSK3β overexpression and increased following GSK3β knockdown or the inhibition of GSK3β by a GSK3β specific inhibitor TWS119 (Fig. 6G-I), and the polyubiquitination of IGF2BP2 was increased when GSK3β was overexpressed, and decreased when GSK3β was silenced or inhibited by TWS119 (Fig. 6J, K). We also studied the role of TPXXT sites in IGF2BP2 using the IGF2BP2-AA mutant. We demonstrated that IGF2BP2-AA failed to bind with GSK3β (Fig. 6L), and its polyubiquitination was not as evident as that of IGF2BP2-WT (Fig. 6M). Moreover, overexpression of GSK3β enhanced the interaction between endogenous IGF2BP2 and FBW7 (Fig. 6N, O). Therefore, these results demonstrate that GSK3β promotes the binding and ubiquitination of IGF2BP2 by FBW7 in lung cancer cells.

### The FBW7/GSK3β/IGF2BP2/SLC7A5 axis modulates radiosensitivity in lung cancer

**Fig. 7:**
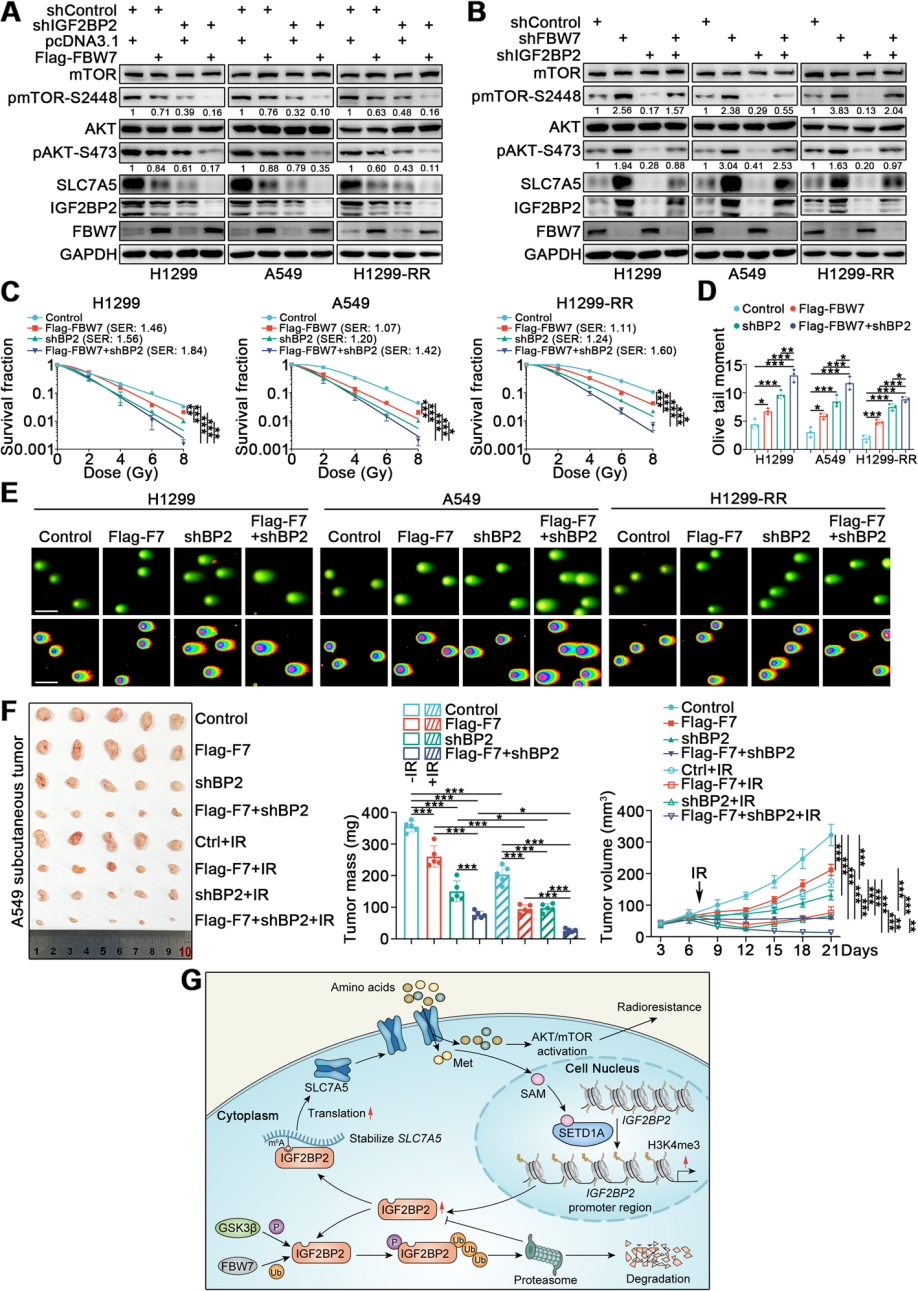
The FBW7/GSK3β/IGF2BP2/SLC7A5 axis modulates radiosensitivity in lung cancer. **A** and **B** H1299, A549, and H1299-RR cells were infected with indicated lentivirus. After puromycin selection, cells were harvested for Western blotting analysis. **C** H1299, A549, and H1299-RR cells were infected with indicated lentivirus. After puromycin selection, cells were irradiated at various doses for clonogenic survival assay. *n* = 3, one-way ANOVA. Data are presented as Mean ± SD. **D **and** E** H1299, A549, and H1299-RR cells were infected with indicated lentivirus. After puromycin selection, cells were irradiated at 6 Gy. After 4 h, cells were harvested for neutral comet assay. *n* = 3, one-way ANOVA. Data are presented as Mean ± SD. Scale bar, 50 μm. **F** A549 cells were transfected with indicated constructs. After puromycin selection, cells were injected subcutaneously into nude mice. The mice were treated with or without IR (10 Gy). Tumor volumes were measured every 3 days. Tumors were harvested, photographed, and weighted at day 21. *n* = 5, one-way ANOVA. Data are presented as Mean ± SD. **G** The schematic diagram depicting that FBW7 together with GSK3β promote IGF2BP2 degradation, thus inhibit the IGF2BP2-SLC7A5 feedback loop and its effect on promoting lung cancer radioresistance. *, *P* < 0.05; **, *P* < 0.01; ***, *P* < 0.001. Abbreviations: BP2, IGF2BP2; F7, FBW7

The above data provide evidence that FBW7/GSK3β regulate the protein levels of IGF2BP2, and the IGF2BP2-SLC7A5 feedback loop participates in lung cancer radioresistance. Hence, we surmised that the FBW7/GSK3β/IGF2BP2/SLC7A5 axis may modulate lung cancer radiosensitivity. We observed that FBW7 overexpression resulted in decreased protein levels of IGF2BP2 and SLC7A5, and decreased phosphorylation of mTOR and AKT, which was a similar effect to that of IGF2BP2 knockdown. In addition, overexpression of FBW7 after IGF2BP2 silencing further decreased IGF2BP2 protein levels, SLC7A5 protein levels, and mTOR and AKT phosphorylation (Fig. 7A). Conversely, FBW7 silencing increased the protein levels of IGF2BP2, SLC7A5, and phosphorylated mTOR and AKT, which was the opposite effect of IGF2BP2 knockdown, and FBW7 silencing after IGF2BP2 knockdown resulted in a slight increase in IGF2BP2, SLC7A5, and phosphorylated mTOR and AKT levels (Fig. 7B). Notably, the clonogenic survival assay, Rad51 foci formation assay, neutral comet assay, and Annexin V/PI assay demonstrated that FBW7 overexpression and IGF2BP2 silencing resulted in impaired survival and DNA damage repair, with increased IR-induced DNA damage and apoptosis rate; these effects were enhanced by FBW7 overexpression plus IGF2BP2 knockdown (Fig. 7C-E and Supplementary Fig. 4A, B). Consistently, the in vivo subcutaneous tumor model demonstrated that FBW7 overexpression and IGF2BP2 knockdown could inhibit tumor growth and enhance the antitumor effect of IR, with the best effects obtained by combining FBW7 overexpression and IGF2BP2 knockdown (Fig. 7F). Taken together, our results demonstrate that FBW7 functions alongside GSK3β to promote IGF2BP2 degradation, thus inhibiting the IGF2BP2-SLC7A5 feedback loop and impairing lung cancer radioresistance (Fig. 7G).

## Discussion

Radiotherapy is the main treatment for early-stage inoperable NSCLC patients, and radiochemotherapy is the standard therapy for unresectable locally advanced NSCLC [3, 29]. Despite advances in treatment, the efficacy still needs to be improved. Recurrence and metastasis are common among early-stage NSCLC patients [29, 30], and the OS at 5 years in unresectable locally advanced NSCLC patients remains low at 10–15% [31]. The poor efficacy is largely due to the development of resistance to IR. Radioresistance can be caused by various mechanisms, including DNA damage repair [32, 33], tumor microenvironment remodeling [34], cell senescence [35, 36], immune response [34, 37], and metabolic reprogramming [38]. The molecular regulatory networks underlying these mechanisms are complicated, and there remains a significant need for further exploration. In the present study, we identified a novel m^6^A-dependent mechanism induced by an m^6^A “reader” IGF2BP2 that drives lung cancer radioresistance.

IGF2BP2 protects its target mRNAs from degradation in the P-body, the location of mRNA fate decisions [39], while facilitates the translation of its target mRNAs [9]. IGF2BP2 has been considered oncogenic, because the upregulation of IGF2BP2 results in the abnormal accumulation of various oncoproteins. For instance, in breast cancer stem-like cells, AURKA strengthens the binding of IGF2BP2 to stabilize m^6^A-modified *DROSHA*, resulting in the maintenance of breast cancer stemness [40]. Although different mechanisms of IGF2BP2 function have been elucidated, the specific role of IGF2BP2 in lung cancer radioresistance remains understudied.

In this study, we demonstrated that IGF2BP2 promotes LUAD radioresistance both in vitro and in vivo. We identified *SLC7A5* as an mRNA target of IGF2BP2. Specifically, we confirmed that IGF2BP2 binds to the m^6^A sites of *SLC7A5* mRNA to enhance its stability and translation, in an m^6^A-dependent manner. SLC7A5 is a transporter mediating the import of large neutral amino acids into cells [41]. SLC7A5 functions as part of a heterodimeric complex on the cell membrane, covalently bound to SLC3A2 (also known as CD98hc or 4F2hc) [42]. SLC7A5 is highly expressed in many cancer types and has been shown to be oncogenic, largely due to its ability to import leucine, which promotes cell growth by activating the AKT/mTOR pathway [22]. On the other hand, the SLC3A2 subunit can also act via the mTOR pathway to promote ATF4 expression; ATF4 is essential for the maintenance of intracellular glutathione (GSH) pool to protect cells from oxidative damage and enhance DNA damage repair [27]. Accordingly, SLC3A2 has been found to regulate radiosensitivity in head and neck squamous cell carcinoma [43]. In our study, we demonstrated that the IGF2BP2-mediated upregulation of SLC7A5 promotes LUAD radioresistance through AKT/mTOR pathway activation.

We also found that the increased Met influx caused by upregulated SLC7A5 plays a role in histone methylation. Mechanistically, the universal methyl donor SAM, which is produced from Met, is utilized by a component of the histone methyltransferase (HMT) complex SETD1A to increase the H3K4me3 level at the *IGF2BP2* promoter region. This results in *IGF2BP2* transcriptional activation. Therefore, we demonstrated that the regulation between IGF2BP2 and SLC7A5 is reciprocal, forming a positive feedback loop to promote LUAD radioresistance. In fact, the reciprocal relationship between amino acid transporters and epigenetic alterations has attracted significant recent attention. For instance, Dann et al.provided evidence for a co-regulation model of SLC7A5 and EZH2, in which SLC7A5 influences the EZH2-mediated methylation of lysine 27 of histone H3 (H3K27), while EZH2 in turn regulates *SLC7A5* transcription [44]. Our findings shed new light on this field.

The current study also highlights the regulatory factor of IGF2BP2. We found that FBW7, an E3 ubiquitin ligase, functions with a kinase GSK3β to participate in the degradation of IGF2BP2. Intriguingly, FBW7 is deleted in approximately 30% of human cancers, mutated in approximately 6% of human cancers, and its expression could be reduced by mutated p53 [28]. Thus, it is feasible that there exists a correlation between the aberrant overexpression of IGF2BP2 and the relatively low activity of FBW7 in lung cancer.

This study had certain limitations. In order to identify why IGF2BP2 overexpressed in lung cancer, we focused on FBW7 mediating IGF2BP2 ubiquitinated degradation. Due to lack of proteomics data, our study did not show whether other E3 ubiquitin ligases were involved in it. Another limitation of this study was that we have not yet identified small-molecule inhibitors targeting IGF2BP2 in lung cancer therapy. Although there are currently no commercial inhibitors of IGF2BP2, a few reports recently identified some small-molecule IGF2BP2 inhibitors in colorectal cancer [45] and T-cell acute lymphoblastic leukemia [46]. These works are required to be further completed.

## Conclusions

Overall, our study explicitly identified a positive feedback loop involving the m^6^A “reader” IGF2BP2 and the amino acid transporter SLC7A5 in lung cancer radioresistance. Specifically, the FBW7/GSK3β/IGF2BP2/SLC7A5 axis was characterized and shown to play a vital role in lung cancer radiosensitivity. Our findings suggest that IGF2BP2 may be a potential therapeutic target to overcome lung cancer radioresistance, which may inform future advances in lung cancer therapy.

### Supplementary Information

**Additional file 1:** **Supplementary Figure 1.** IGF2BP2 is overexpressed in radioresistant lung cancer cells and promotes radioresistance in lung cancer, related to Figure 1. **Supplementary Figure 2.** IGF2BP2 enhances *SLC7A5* mRNA stability through an m^6^A-dependent mechanism in lung cancer cells, related to Figure 2. **Supplementary Figure 3.** The IGF2BP2-SLC7A5 positive feedback loop promotes radioresistance in lung cancer, related to Figure 4. **Supplementary Figure 4.** The FBW7/GSK3β/IGF2BP2/SLC7A5 axis modulates radiosensitivity in lung cancer, related to Figure 7. **Table S1.** Sequences for shRNAs and siRNAs. **Table S2.** Sequences for primers used for RT-qPCR. **Table S3.** Sequences for primers used for ChIP-qPCR. **Table S4.** RNA-seq data of siIGF2BP2 vs. siControl.

## Data Availability

The RNA-seq data is provided in Supplementary Table S4.

## Abbreviations

FBW7: F-box and WD repeat domain-containing 7
GSK3β: Glycogen synthase kinase-3 beta
IGF2BP2: Insulin Like Growth Factor 2 mRNA Binding Protein 2
SLC7A5: Solute Carrier Family 7 Member 5
m^6^A: N^6^-Methyladenosine
ChIP: Chromatin immunoprecipitation
RIP: RNA immunoprecipitation
MeRIP: Methylated RNA immunoprecipitation
H3K4me3: Trimethyl modification at lysine 4 of histone H3
NSCLC: Non-small cell lung cancer
LUAD: Lung adenocarcinoma
mTOR: Mammalian target of rapamycin
Met: Methionine
SAM: S-adenosylmethionin
co-IP: Co-immunoprecipitation
siRNA: Small interfering RNA
shRNA: Short hairpin RNA
RNA-seq: Transcriptome sequencing
SER: Sensitization enhancement ratio
IHC: Immunohistochemistry
DEG: Differentially expressed gene
RR: Radioresistant
IR: Irradiation
