# Supplementary material for: FBW7/GSK3β mediated degradation of IGF2BP2 inhibits IGF2BP2-SLC7A5 positive feedback loop and radioresistance in lung cancer
Source: J Exp Clin Cancer Res. 2024 Jan 29;43:34. doi: 10.1186/s13046-024-02959-3 (PMC10823633; doi:10.1186/s13046-024-02959-3)
Supplement: Supplementary file 1 — Additional file 1: Supplementary Figure 1. IGF2BP2 is overexpressed in radioresistant lung cancer cells and promotes radioresistance in lung cancer, related to Figure 1. Supplementary Figure 2. IGF2BP2 enhances SLC7A5 mRNA stability through an m6A-dependent mechanism in lung cancer cells, related to Figure 2. Supplementary Figure 3. The IGF2BP2-SLC7A5 positive feedback loop promotes radioresistance in lung cancer, related to Figure 4. Supplementary Figure 4. The FBW7/GSK3β/IGF2BP2/SLC7A5 axis modulates radiosensitivity in lung cancer, related to Figure 7. Table S1. Sequences for shRNAs and siRNAs. Table S2. Sequences for primers used for RT-qPCR. Table S3. Sequences for primers used for ChIP-qPCR. Table S4. RNA-seq data of siIGF2BP2 vs. siControl. [file 13046_2024_2959_MOESM1_ESM.docx]

**Supplementary Information for:**

**FBW7/GSK3β mediated degradation of IGF2BP2 inhibits IGF2BP2-SLC7A5 positive feedback loop and radioresistance in lung cancer**

Zhiyuan Zhou, Bin Zhang, Yue Deng, Suke Deng, Jie Li, Wenwen Wei, Yijun Wang, Jiacheng Wang, Zishan Feng, Mengjie Che, Xiao Yang, Jingshu Meng, Yan Li, Yan Hu, Yajie Sun, Lu Wen, Fang Huang, Yuhan Sheng, Chao Wan, Kunyu Yang

**Including:**

Supplementary Figures 1-4

Supplementary Tables S1-S4


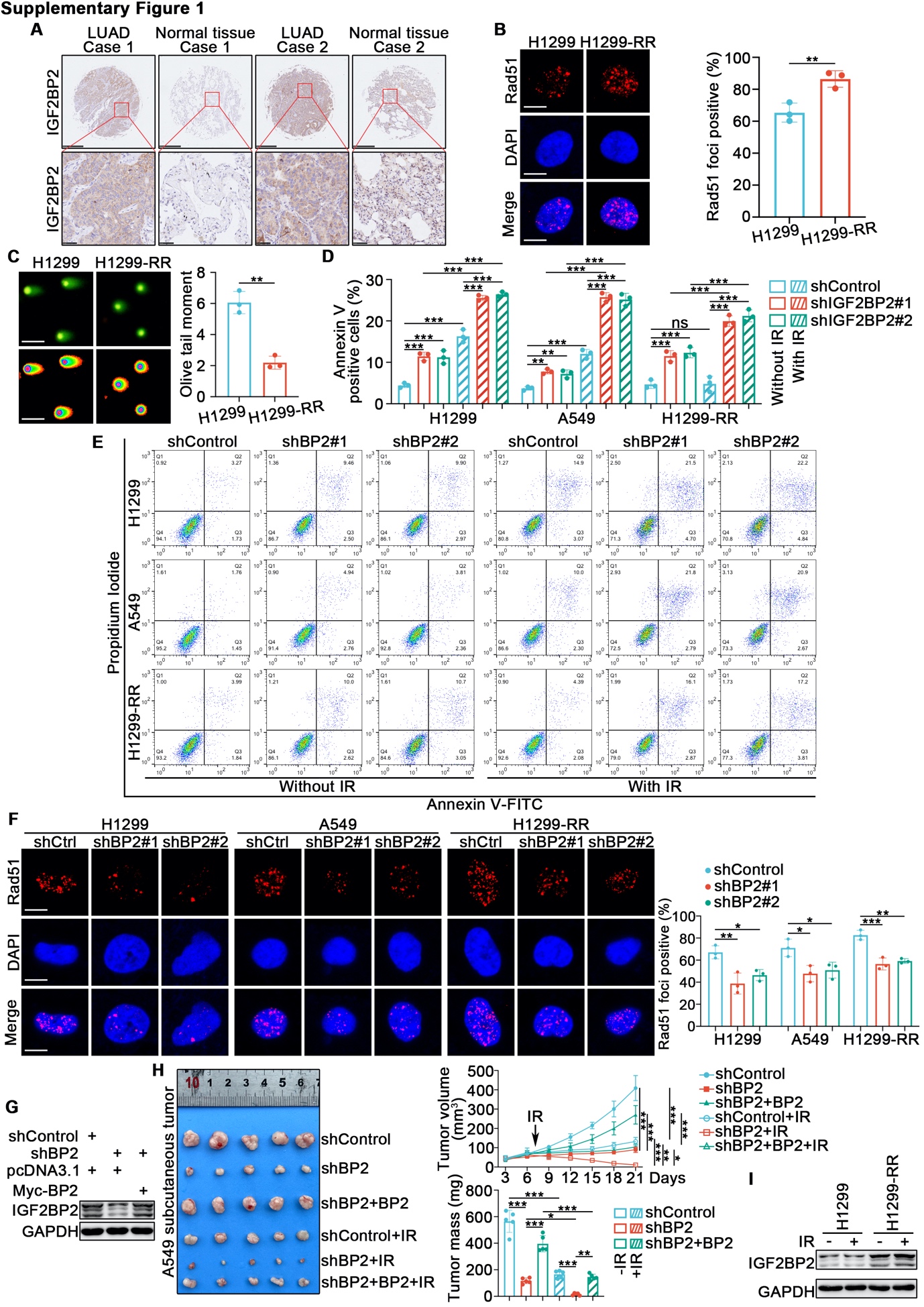


**Supplementary Figure 1. IGF2BP2 is overexpressed in radioresistant lung cancer cells and promotes radioresistance in lung cancer, related to Figure 1.**

**A,** The representative images of IHC for IGF2BP2 protein on LUAD tissue microarray. Normal *n* = 15, cancer *n* = 61. Scale bars, 500 μm for low power field, 50 μm for high power field.

**B and C,** H1299 and H1299-RR cells were irradiated at 6 Gy. After 4 h, cells were collected for Rad51 foci formation assay (**B**), and neutral comet assay (**C**). *n* = 3, unpaired t test. Data are presented as Mean ± SD. Scale bars, 10 μm in **B**, 50 μm in **C**.

**D and E,** H1299, A549, and H1299-RR cells infected with indicated lentivirus vectors, after puromycin selection, were treated with or without IR (6 Gy). After 48 h, cells were collected for Annexin V/PI assay. *n* = 3, one-way ANOVA. Data are presented as Mean ± SD.

**F,** H1299, A549, and H1299-RR cells infected with indicated lentivirus vectors, after puromycin selection, were irradiated at 6 Gy. After 4 h, cells were collected for Rad51 foci formation assay. *n* = 3, one-way ANOVA. Data are presented as Mean ± SD. Scale bar, 10 μm.

**G,** A549 cells were transfected with indicated constructs. After puromycin selection, cells were harvested for Western blotting analysis.

**H,** A549 cells were transfected with indicated constructs. After puromycin selection, cells were injected subcutaneously into nude mice. The mice were treated with or without IR (10 Gy). Tumor volumes were measured every 3 days. Tumors were harvested, photographed, and weighted at day 21. *n* = 5, one-way ANOVA. Data are presented as Mean ± SD.

**I,** Western blotting analysis was performed in H1299 and H1299-RR cells with or without IR (10 Gy).

ns, not significant, *P* > 0.05; *, *P* < 0.05; **, *P* < 0.01; ***, *P* < 0.001.

Abbreviations: BP2, IGF2BP2.


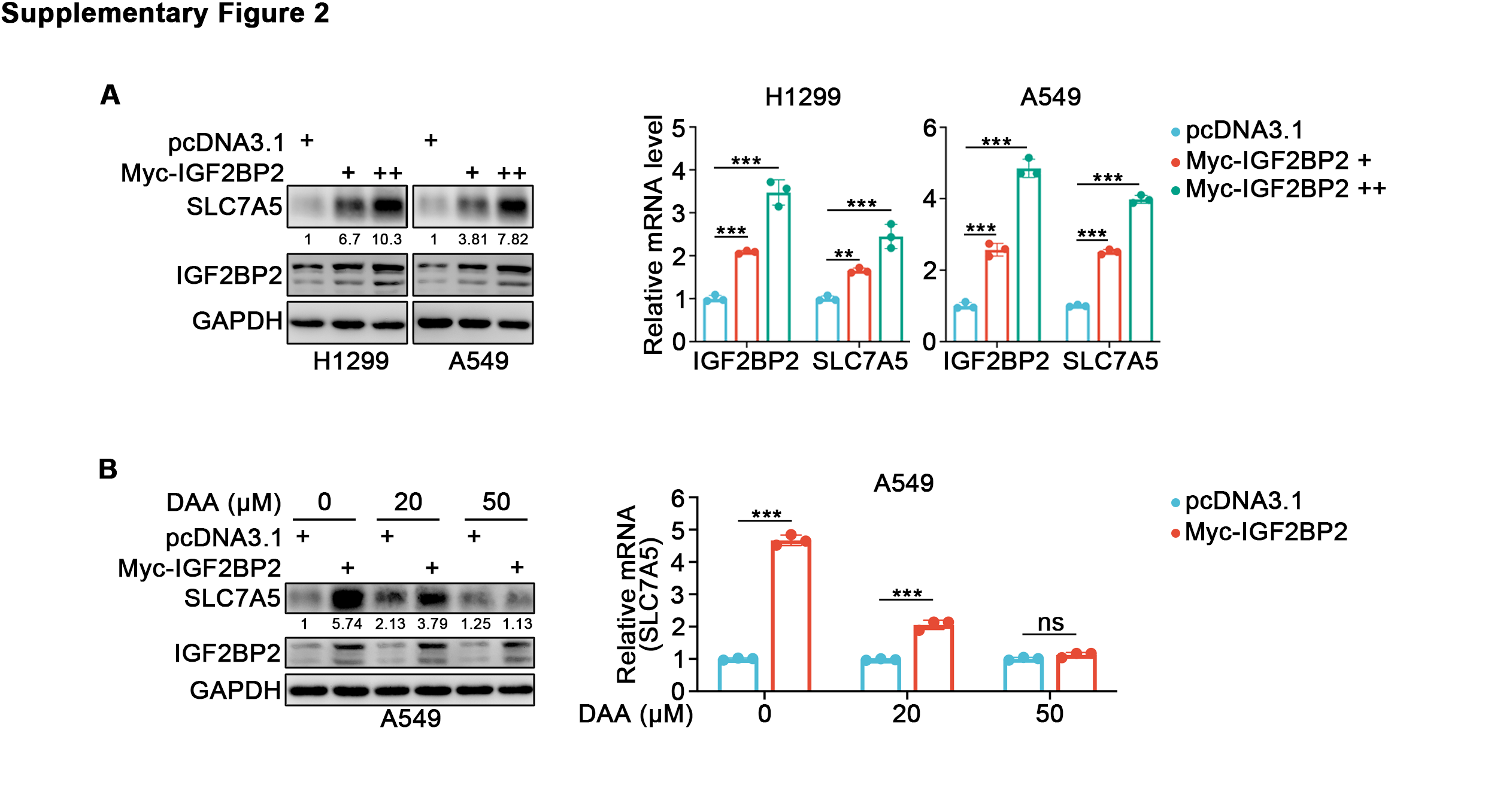


**Supplementary Figure 2. IGF2BP2 enhances *SLC7A5* mRNA stability through an m^6^A-dependent mechanism in lung cancer cells, related to Figure 2.**

**A,** H1299 and A549 cells were infected with indicated lentivirus. After puromycin selection, cells were harvested for Western blotting and RT-qPCR analyses. *n* = 3, one-way ANOVA. Data are presented as Mean ± SD.

**B,** A549 cells were infected with indicated lentivirus. After puromycin selection, cells were treated with different concentrations of DAA (0, 20, and 50 μM), and were harvested for Western blotting and RT-qPCR analyses. *n* = 3, one-way ANOVA. Data are presented as Mean ± SD.

ns, not significant, *P* > 0.05; **, *P* < 0.01; ***, *P* < 0.001.


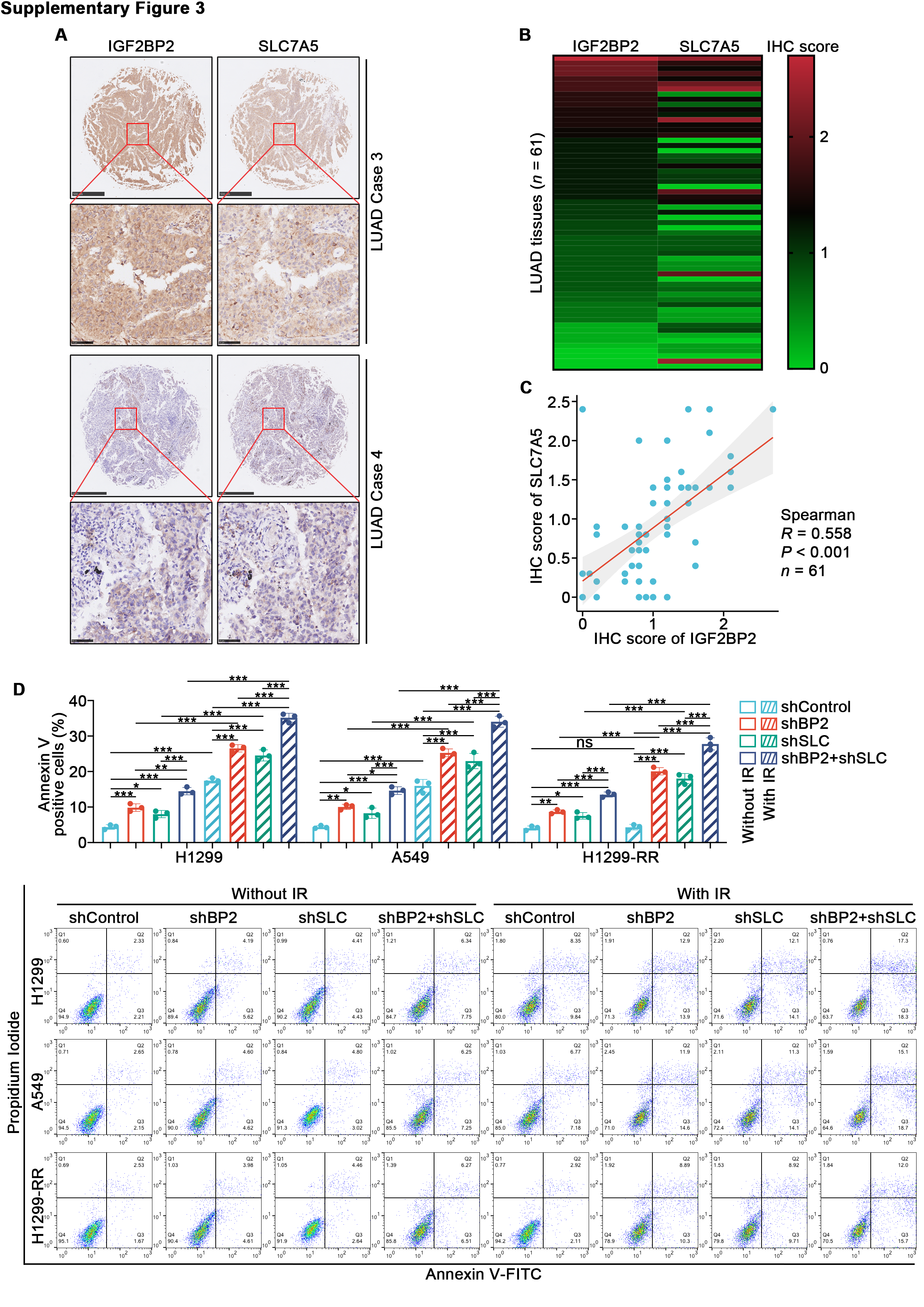


**Supplementary Figure 3. The IGF2BP2-SLC7A5 positive feedback loop promotes radioresistance in lung cancer, related to Figure 4.**

**A,** The representative images of IHC for IGF2BP2 and SLC7A5 protein on LUAD tissue microarray (*n* = 61). Scale bars, 500 μm for low power field, 50 μm for high power field.

**B,** The heatmap showing IHC score of IGF2BP2 and SLC7A5 protein in LUAD tissue microarray (*n* = 61).

**C,** The correlation analysis of IHC score among IGF2BP2 and SLC7A5 in LUAD tissue microarray (*n* = 61).

**D,** H1299, A549, and H1299-RR cells infected with indicated lentivirus vectors, after puromycin selection, were treated with or without IR (6 Gy). After 48 h, cells were collected for Annexin V/PI assay. *n* = 3, one-way ANOVA. Data are presented as Mean ± SD.

ns, not significant, *P* > 0.05; *, *P* < 0.05; **, *P* < 0.01; ***, *P* < 0.001.

Abbreviations: BP2, IGF2BP2; SLC, SLC7A5.


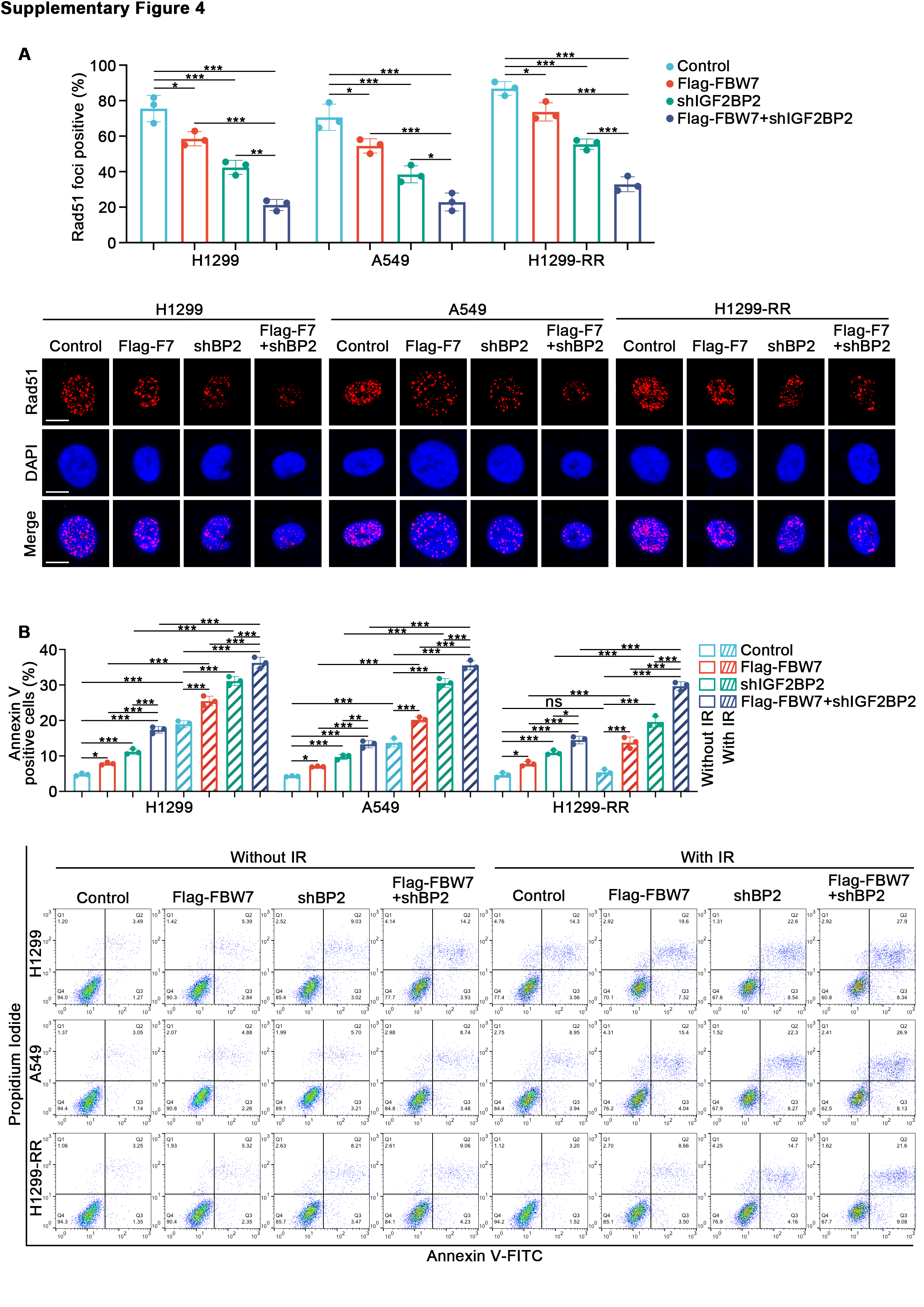


**Supplementary Figure 4. The FBW7/GSK3β/IGF2BP2/SLC7A5 axis modulates radiosensitivity in lung cancer, related to Figure 7.**

**A,** H1299, A549, and H1299-RR cells were infected with indicated lentivirus vectors. After puromycin selection, cells were irradiated at 6 Gy. After 4 h, cells were collected for Rad51 foci formation assay. *n* = 3, one-way ANOVA. Data are presented as Mean ± SD. Scale bar, 10 μm.

**B,** H1299, A549, and H1299-RR cells were infected with indicated lentivirus vectors. After puromycin selection, cells were treated with or without IR (6 Gy). After 48 h, cells were collected for Annexin V/PI assay. *n* = 3, one-way ANOVA. Data are presented as Mean ± SD.

ns, not significant, *P* > 0.05; *, *P* < 0.05; **, *P* < 0.01; ***, *P* < 0.001.

Abbreviations: BP2, IGF2BP2; F7, FBW7.

**Table S1. Sequences for shRNAs and siRNAs**

**Sequences for shRNAs:**

| shIGF2BP2#1 | 5’-AGTGAAGCTGGAAGCGCATAT-3’ |
| --- | --- |
| shIGF2BP2#2 | 5’-TTCCCGCATCATCACTCTTAT-3’ |
| shMETTL3 | 5’-GCCTTAACATTGCCCACTGAT-3’ |
| shSLC7A5#1 | 5’-GCATTATACAGCGGCCTCTTT-3’ |
| shSLC7A5#2 | 5’-CTAGATCCCAACTTCTCATTT-3’ |
| shSETD1A#1 | 5’-GGAAAGAGCCATCGGAAATTT-3’ |
| shSETD1A#2 | 5’-GACAACAACGAATGAAATATT-3’ |
| shFBW7#1 | 5’-ACAGGACAGTGTTTACAAA-3’ |
| shFBW7#2 | 5’-CAACAACGACGCCGAATTA-3’ |
| shGSK3β#1 | 5’-CCCAAACTACACAGAATTTAA-3’ |
| shGSK3β#2 | 5’-AGCAAATCAGAGAAATGAAC-3’ |

**Sequences for siRNAs:**

| siIGF2BP2#1 | 5’-AGTGAAGCTGGAAGCGCATAT-3’ |
| --- | --- |
| siIGF2BP2#2 | 5’-TTCCCGCATCATCACTCTTAT-3’ |

**Table S2. Sequences for primers used for RT-qPCR**

| **Species** | **Gene** | **Forward (5’-3’)** | **Reverse (5’-3’)** |
| --- | --- | --- | --- |
| Human | *GAPDH* | CCAGAACATCATCCCTGCCT | CCTGCTTCACCACCTTCTTG |
| Human | *β-Actin* | GCAGAAGGAGATCACTGCCCT | GCTGATCCACATCTGCTGGAA |
| Human | *IGF2BP2* | AGTGGAATTGCATGGGAAAATCA | CAACGGCGGTTTCTGTGTC |
| Human | *SLC7A5* | TGTTTTCAAGCACAACCC | AATAAACATGGTGCCGACA |

**Table S3. Sequences for primers used for ChIP-qPCR**

| **Species** | **Gene** | **Forward (5’-3’)** | **Reverse (5’-3’)** |
| --- | --- | --- | --- |
| Human | *IGF2BP2*  *(Primer I)* | GGAAGACGCGGATGATGAAC | GGTAGTCCACGAAGGCGTA |
| Human | *IGF2BP2*  *(Primer O)* | CATATTCCAGGGTCCCGCTC | CACCTGCCACCTACTGAGTG |

**Table S4. RNA-seq data of siIGF2BP2 vs. siControl**

| gene_id | si_IGF2BP2 | si_Control | log2FoldChange | pvalue | padj | gene_name | gene_biotype |
| --- | --- | --- | --- | --- | --- | --- | --- |
| ENSG00000134333 | 7632.65208 | 30212.1975 | -1.9847861 | 0 | 0 | LDHA | protein_coding |
| ENSG00000148730 | 512.512539 | 3001.05934 | -2.5510596 | 1.02E-219 | 1.19E-215 | EIF4EBP2 | protein_coding |
| ENSG00000118523 | 862.262356 | 3707.05606 | -2.104251 | 2.96E-219 | 2.30E-215 | CTGF | protein_coding |
| ENSG00000196937 | 9112.76733 | 3226.78926 | 1.49767651 | 1.42E-206 | 8.26E-203 | FAM3C | protein_coding |
| ENSG00000124795 | 3051.45479 | 8197.15879 | -1.4255571 | 9.54E-194 | 4.45E-190 | DEK | protein_coding |
| ENSG00000006534 | 2018.67574 | 376.194833 | 2.42453895 | 1.97E-183 | 7.67E-180 | ALDH3B1 | protein_coding |
| ENSG00000204054 | 4289.74556 | 1174.18839 | 1.8698526 | 1.82E-180 | 6.06E-177 | LINC00963 | processed_transcript |
| ENSG00000167460 | 8380.34609 | 2727.84813 | 1.61972249 | 1.77E-168 | 5.17E-165 | TPM4 | protein_coding |
| ENSG00000074695 | 795.939766 | 3187.48784 | -2.0009568 | 1.74E-164 | 4.51E-161 | LMAN1 | protein_coding |
| ENSG00000103257 | 1435.85134 | 6476.89561 | -2.1731296 | 2.61E-161 | 6.09E-158 | SLC7A5 | protein_coding |
| ENSG00000080824 | 26297.161 | 62128.069 | -1.2403253 | 8.78E-161 | 1.86E-157 | HSP90AA1 | protein_coding |
| ENSG00000143401 | 1359.90813 | 4012.45693 | -1.5607814 | 1.83E-153 | 3.56E-150 | ANP32E | protein_coding |
| ENSG00000148677 | 99.3910943 | 1007.52676 | -3.3410018 | 2.15E-141 | 3.87E-138 | ANKRD1 | protein_coding |
| ENSG00000189403 | 2701.01224 | 6862.57424 | -1.3449089 | 1.96E-139 | 3.27E-136 | HMGB1 | protein_coding |
| ENSG00000011426 | 2334.39952 | 6138.93384 | -1.3948807 | 1.26E-137 | 1.96E-134 | ANLN | protein_coding |
| ENSG00000151239 | 2915.1573 | 7215.52357 | -1.3074225 | 2.55E-132 | 3.71E-129 | TWF1 | protein_coding |
| ENSG00000145907 | 2434.61176 | 6448.08749 | -1.404937 | 3.83E-130 | 5.25E-127 | G3BP1 | protein_coding |
| ENSG00000149547 | 447.853109 | 2174.48488 | -2.2791179 | 2.34E-122 | 3.04E-119 | EI24 | protein_coding |
| ENSG00000073792 | 869.820815 | 2664.4514 | -1.6147353 | 3.42E-122 | 4.19E-119 | IGF2BP2 | protein_coding |
| ENSG00000242265 | 2295.85449 | 6085.03711 | -1.4057431 | 8.95E-121 | 1.04E-117 | PEG10 | protein_coding |
| ENSG00000136824 | 793.397389 | 2557.53847 | -1.6879133 | 2.29E-114 | 2.55E-111 | SMC2 | protein_coding |
| ENSG00000134690 | 624.694425 | 2134.62288 | -1.7716474 | 9.76E-107 | 1.04E-103 | CDCA8 | protein_coding |
| ENSG00000272405 | 1999.11461 | 587.334601 | 1.76771647 | 4.29E-106 | 4.35E-103 | AL365181.3 | antisense |
| ENSG00000176171 | 1837.68277 | 5028.72388 | -1.4520694 | 2.07E-105 | 2.01E-102 | BNIP3 | protein_coding |
| ENSG00000213064 | 807.962151 | 2562.00513 | -1.664083 | 5.43E-105 | 5.06E-102 | SFT2D2 | protein_coding |
| ENSG00000171848 | 859.559669 | 2841.53789 | -1.7250405 | 1.06E-104 | 9.54E-102 | RRM2 | protein_coding |
| ENSG00000114850 | 2580.65904 | 5682.80821 | -1.1385654 | 1.31E-102 | 1.09E-99 | SSR3 | protein_coding |
| ENSG00000182481 | 3061.34129 | 6885.98977 | -1.1694842 | 6.55E-101 | 5.09E-98 | KPNA2 | protein_coding |
| ENSG00000156802 | 1979.42043 | 4653.68066 | -1.2331577 | 2.03E-100 | 1.53E-97 | ATAD2 | protein_coding |
| ENSG00000169504 | 7495.29366 | 15042.0894 | -1.0048492 | 3.66E-99 | 2.67E-96 | CLIC4 | protein_coding |
| ENSG00000149480 | 775.50003 | 2293.81636 | -1.564163 | 4.35E-99 | 3.08E-96 | MTA2 | protein_coding |
| ENSG00000142949 | 5968.60291 | 14763.0897 | -1.3063866 | 1.83E-97 | 1.25E-94 | PTPRF | protein_coding |
| ENSG00000111669 | 5215.8834 | 14161.2897 | -1.4408812 | 5.84E-97 | 3.89E-94 | TPI1 | protein_coding |
| ENSG00000168411 | 705.662668 | 2351.05156 | -1.7366954 | 1.28E-96 | 8.28E-94 | RFWD3 | protein_coding |
| ENSG00000165732 | 2418.51605 | 5341.05588 | -1.1428771 | 2.72E-95 | 1.71E-92 | DDX21 | protein_coding |
| ENSG00000139734 | 389.028724 | 1433.17113 | -1.8818878 | 6.97E-95 | 4.28E-92 | DIAPH3 | protein_coding |
| ENSG00000148773 | 2156.08023 | 5410.94279 | -1.3269535 | 1.11E-94 | 6.63E-92 | MKI67 | protein_coding |
| ENSG00000113810 | 2186.31407 | 5424.01987 | -1.3106473 | 1.71E-93 | 9.97E-91 | SMC4 | protein_coding |
| ENSG00000102144 | 7360.78834 | 16720.5173 | -1.1836163 | 2.45E-93 | 1.39E-90 | PGK1 | protein_coding |
| ENSG00000120802 | 2517.68288 | 5324.76801 | -1.080766 | 7.25E-93 | 4.03E-90 | TMPO | protein_coding |
| ENSG00000083312 | 2727.75291 | 5845.77944 | -1.099539 | 1.98E-92 | 1.07E-89 | TNPO1 | protein_coding |
| ENSG00000119681 | 2425.86564 | 805.725598 | 1.5908433 | 8.75E-92 | 4.64E-89 | LTBP2 | protein_coding |
| ENSG00000198959 | 1795.19664 | 4946.89965 | -1.4621247 | 1.67E-91 | 8.63E-89 | TGM2 | protein_coding |
| ENSG00000117394 | 1330.74779 | 4050.6496 | -1.6056163 | 9.04E-89 | 4.59E-86 | SLC2A1 | protein_coding |
| ENSG00000089902 | 1055.11874 | 2778.78156 | -1.3963734 | 2.31E-86 | 1.15E-83 | RCOR1 | protein_coding |
| ENSG00000100528 | 661.514608 | 1884.29637 | -1.5097848 | 2.85E-86 | 1.38E-83 | CNIH1 | protein_coding |
| ENSG00000111252 | 1728.18196 | 3829.65639 | -1.1478201 | 5.43E-86 | 2.59E-83 | SH2B3 | protein_coding |
| ENSG00000085449 | 738.354575 | 2239.4825 | -1.6002888 | 1.82E-84 | 8.50E-82 | WDFY1 | protein_coding |
| ENSG00000117399 | 2156.14715 | 5207.88794 | -1.2721439 | 1.43E-83 | 6.55E-81 | CDC20 | protein_coding |
| ENSG00000101224 | 1802.55419 | 4241.00333 | -1.2340238 | 2.97E-83 | 1.33E-80 | CDC25B | protein_coding |
| ENSG00000110108 | 883.575865 | 2750.3583 | -1.6386408 | 1.50E-82 | 6.60E-80 | TMEM109 | protein_coding |
| ENSG00000111057 | 4019.25645 | 9322.51359 | -1.2137867 | 1.37E-81 | 5.92E-79 | KRT18 | protein_coding |
| ENSG00000117500 | 1005.07658 | 2444.16315 | -1.2815273 | 1.69E-81 | 7.15E-79 | TMED5 | protein_coding |
| ENSG00000082153 | 3270.85612 | 6674.73197 | -1.0293081 | 2.76E-81 | 1.15E-78 | BZW1 | protein_coding |
| ENSG00000134287 | 1479.13836 | 3881.96186 | -1.3914832 | 1.42E-80 | 5.81E-78 | ARF3 | protein_coding |
| ENSG00000131747 | 1885.22967 | 4122.68239 | -1.1284812 | 1.79E-80 | 7.20E-78 | TOP2A | protein_coding |
| ENSG00000087586 | 1733.56603 | 3943.02389 | -1.1854289 | 3.96E-80 | 1.57E-77 | AURKA | protein_coding |
| ENSG00000104738 | 2717.36404 | 5916.13775 | -1.1221288 | 2.56E-78 | 9.97E-76 | MCM4 | protein_coding |
| ENSG00000180488 | 540.630109 | 1561.00524 | -1.5294313 | 3.82E-78 | 1.46E-75 | MIGA1 | protein_coding |
| ENSG00000170312 | 1551.06886 | 3563.8845 | -1.1996768 | 2.36E-77 | 8.87E-75 | CDK1 | protein_coding |
| ENSG00000142945 | 1457.43576 | 3370.25872 | -1.2091546 | 5.43E-77 | 2.01E-74 | KIF2C | protein_coding |
| ENSG00000144959 | 981.862681 | 2384.8205 | -1.2804324 | 9.97E-77 | 3.63E-74 | NCEH1 | protein_coding |
| ENSG00000114346 | 1642.30636 | 3697.76899 | -1.1706822 | 2.90E-76 | 1.04E-73 | ECT2 | protein_coding |
| ENSG00000065923 | 5173.03286 | 2493.03635 | 1.05328233 | 4.69E-76 | 1.63E-73 | SLC9A7 | protein_coding |
| ENSG00000135476 | 1292.29898 | 3091.69438 | -1.2577122 | 8.37E-76 | 2.87E-73 | ESPL1 | protein_coding |
| ENSG00000122861 | 3000.72966 | 6824.71283 | -1.1855509 | 1.98E-74 | 6.70E-72 | PLAU | protein_coding |
| ENSG00000181061 | 192.077418 | 845.187369 | -2.1364892 | 4.41E-74 | 1.47E-71 | HIGD1A | protein_coding |
| ENSG00000100697 | 2330.75836 | 5372.46105 | -1.2043302 | 3.91E-73 | 1.28E-70 | DICER1 | protein_coding |
| ENSG00000169679 | 1245.50627 | 2770.98343 | -1.1536107 | 4.76E-73 | 1.54E-70 | BUB1 | protein_coding |
| ENSG00000175322 | 455.806921 | 1485.59285 | -1.7056496 | 5.87E-73 | 1.88E-70 | ZNF519 | protein_coding |
| ENSG00000123485 | 765.611367 | 1944.08433 | -1.3440497 | 4.18E-72 | 1.30E-69 | HJURP | protein_coding |
| ENSG00000080819 | 466.418189 | 1432.92351 | -1.6188363 | 4.42E-72 | 1.36E-69 | CPOX | protein_coding |
| ENSG00000171793 | 1659.65387 | 3697.63411 | -1.1556712 | 6.93E-72 | 2.10E-69 | CTPS1 | protein_coding |
| ENSG00000096063 | 1294.54916 | 2960.93929 | -1.1932716 | 2.70E-71 | 8.08E-69 | SRPK1 | protein_coding |
| ENSG00000186432 | 2098.38179 | 4243.55899 | -1.0159169 | 2.77E-70 | 8.18E-68 | KPNA4 | protein_coding |
| ENSG00000133606 | 574.082968 | 1706.35474 | -1.5705313 | 4.46E-70 | 1.30E-67 | MKRN1 | protein_coding |
| ENSG00000129128 | 1199.06731 | 3022.11075 | -1.3328338 | 7.41E-70 | 2.13E-67 | SPCS3 | protein_coding |
| ENSG00000171298 | 1722.82849 | 617.058361 | 1.48183443 | 2.34E-69 | 6.67E-67 | GAA | protein_coding |
| ENSG00000050405 | 1596.75767 | 3706.66094 | -1.2143607 | 5.65E-69 | 1.59E-66 | LIMA1 | protein_coding |
| ENSG00000127528 | 437.654565 | 1481.61197 | -1.7585092 | 3.27E-67 | 9.09E-65 | KLF2 | protein_coding |
| ENSG00000109805 | 1265.67794 | 2919.07623 | -1.2051711 | 7.59E-67 | 2.08E-64 | NCAPG | protein_coding |
| ENSG00000132646 | 1687.12761 | 3477.16615 | -1.0434369 | 1.86E-66 | 5.06E-64 | PCNA | protein_coding |
| ENSG00000183723 | 1102.65448 | 2668.10722 | -1.2744726 | 2.07E-66 | 5.56E-64 | CMTM4 | protein_coding |
| ENSG00000158864 | 2822.12444 | 1309.31855 | 1.10811088 | 2.45E-66 | 6.50E-64 | NDUFS2 | protein_coding |
| ENSG00000138061 | 549.3421 | 78.9524618 | 2.79934632 | 4.02E-66 | 1.05E-63 | CYP1B1 | protein_coding |
| ENSG00000076554 | 470.30056 | 1651.77034 | -1.8122944 | 5.47E-66 | 1.42E-63 | TPD52 | protein_coding |
| ENSG00000197045 | 727.0642 | 1961.02319 | -1.4311275 | 8.53E-66 | 2.19E-63 | GMFB | protein_coding |
| ENSG00000089597 | 5999.58944 | 2940.73326 | 1.02912041 | 9.90E-66 | 2.51E-63 | GANAB | protein_coding |
| ENSG00000167110 | 2299.24759 | 1025.87356 | 1.16407006 | 1.79E-65 | 4.49E-63 | GOLGA2 | protein_coding |
| ENSG00000186480 | 1908.20677 | 787.432193 | 1.27720987 | 2.31E-65 | 5.73E-63 | INSIG1 | protein_coding |
| ENSG00000005100 | 791.87664 | 1882.06422 | -1.2490109 | 5.89E-65 | 1.43E-62 | DHX33 | protein_coding |
| ENSG00000099942 | 1393.57921 | 3072.20777 | -1.1401369 | 8.17E-65 | 1.97E-62 | CRKL | protein_coding |
| ENSG00000146670 | 640.564188 | 1816.79752 | -1.5038004 | 1.47E-64 | 3.46E-62 | CDCA5 | protein_coding |
| ENSG00000156471 | 1046.32229 | 2762.08843 | -1.4002219 | 5.03E-64 | 1.16E-61 | PTDSS1 | protein_coding |
| ENSG00000196715 | 803.883801 | 1939.19238 | -1.2697948 | 5.29E-64 | 1.21E-61 | VKORC1L1 | protein_coding |
| ENSG00000172115 | 722.600101 | 1977.37105 | -1.451411 | 2.83E-63 | 6.22E-61 | CYCS | protein_coding |
| ENSG00000198843 | 1191.43715 | 2559.55588 | -1.1028533 | 9.74E-63 | 2.12E-60 | SELENOT | protein_coding |
| ENSG00000143164 | 2855.4316 | 1367.64654 | 1.06225065 | 1.80E-62 | 3.88E-60 | DCAF6 | protein_coding |
| ENSG00000174695 | 1094.49314 | 2390.9394 | -1.1275804 | 5.52E-62 | 1.17E-59 | TMEM167A | protein_coding |
| ENSG00000082641 | 3776.61548 | 1805.3736 | 1.06516722 | 8.67E-62 | 1.82E-59 | NFE2L1 | protein_coding |
| ENSG00000171241 | 729.808073 | 1800.59433 | -1.3022443 | 1.27E-61 | 2.65E-59 | SHCBP1 | protein_coding |
| ENSG00000139625 | 2167.89418 | 945.709818 | 1.1960359 | 2.96E-61 | 6.11E-59 | MAP3K12 | protein_coding |
| ENSG00000138160 | 1447.96698 | 3033.25441 | -1.0663376 | 7.00E-61 | 1.43E-58 | KIF11 | protein_coding |
| ENSG00000138180 | 774.742924 | 1902.34472 | -1.2957699 | 8.11E-61 | 1.64E-58 | CEP55 | protein_coding |
| ENSG00000113368 | 2176.58334 | 4361.06986 | -1.0026067 | 2.31E-60 | 4.65E-58 | LMNB1 | protein_coding |
| ENSG00000114019 | 1804.04116 | 4123.79432 | -1.192685 | 3.20E-59 | 6.37E-57 | AMOTL2 | protein_coding |
| ENSG00000056586 | 1101.19196 | 2379.06175 | -1.111092 | 1.19E-58 | 2.33E-56 | RC3H2 | protein_coding |
| ENSG00000134057 | 2391.5638 | 4803.62539 | -1.0059801 | 1.38E-58 | 2.69E-56 | CCNB1 | protein_coding |
| ENSG00000076382 | 990.784724 | 2253.5887 | -1.1852806 | 6.43E-58 | 1.24E-55 | SPAG5 | protein_coding |
| ENSG00000170779 | 2163.12618 | 4988.44222 | -1.2055897 | 2.46E-57 | 4.70E-55 | CDCA4 | protein_coding |
| ENSG00000117395 | 3158.58679 | 6772.18644 | -1.1001798 | 2.82E-57 | 5.35E-55 | EBNA1BP2 | protein_coding |
| ENSG00000145391 | 1567.98983 | 639.203181 | 1.29458806 | 5.59E-57 | 1.05E-54 | SETD7 | protein_coding |
| ENSG00000156970 | 591.262175 | 1588.12355 | -1.4254462 | 8.50E-57 | 1.57E-54 | BUB1B | protein_coding |
| ENSG00000188229 | 5040.87695 | 11012.1641 | -1.1274156 | 2.03E-56 | 3.73E-54 | TUBB4B | protein_coding |
| ENSG00000123473 | 502.214467 | 1387.42637 | -1.4648877 | 2.31E-56 | 4.20E-54 | STIL | protein_coding |
| ENSG00000121152 | 415.321128 | 1160.42046 | -1.4813805 | 3.31E-56 | 5.98E-54 | NCAPH | protein_coding |
| ENSG00000023445 | 427.46992 | 1158.75194 | -1.4382988 | 1.55E-55 | 2.77E-53 | BIRC3 | protein_coding |
| ENSG00000244879 | 1081.54308 | 347.902497 | 1.63517004 | 2.30E-55 | 4.06E-53 | GABPB1-AS1 | antisense |
| ENSG00000123384 | 2932.34489 | 1369.25556 | 1.09936495 | 3.99E-55 | 7.00E-53 | LRP1 | protein_coding |
| ENSG00000168078 | 454.806138 | 1233.78203 | -1.439979 | 5.66E-55 | 9.85E-53 | PBK | protein_coding |
| ENSG00000196396 | 2105.44661 | 4219.90308 | -1.0029091 | 8.34E-55 | 1.44E-52 | PTPN1 | protein_coding |
| ENSG00000196950 | 410.628583 | 1170.33566 | -1.5097351 | 2.57E-54 | 4.40E-52 | SLC39A10 | protein_coding |
| ENSG00000092853 | 405.198366 | 1145.41135 | -1.4991901 | 5.58E-54 | 9.43E-52 | CLSPN | protein_coding |
| ENSG00000124702 | 2419.13741 | 968.575638 | 1.32136603 | 9.02E-54 | 1.51E-51 | KLHDC3 | protein_coding |
| ENSG00000106261 | 2160.00134 | 967.79421 | 1.1590752 | 2.49E-53 | 4.14E-51 | ZKSCAN1 | protein_coding |
| ENSG00000166128 | 123.655581 | 578.330171 | -2.2278986 | 4.73E-53 | 7.82E-51 | RAB8B | protein_coding |
| ENSG00000105223 | 7750.35702 | 3686.1316 | 1.07254463 | 1.05E-52 | 1.71E-50 | PLD3 | protein_coding |
| ENSG00000186185 | 712.718523 | 1692.37348 | -1.2482504 | 1.88E-52 | 3.04E-50 | KIF18B | protein_coding |
| ENSG00000163346 | 1406.81495 | 516.201526 | 1.44762272 | 2.18E-52 | 3.50E-50 | PBXIP1 | protein_coding |
| ENSG00000139291 | 156.551254 | 647.658361 | -2.0482265 | 2.61E-52 | 4.17E-50 | TMEM19 | protein_coding |
| ENSG00000138594 | 624.387246 | 1490.21821 | -1.2553628 | 7.49E-52 | 1.19E-49 | TMOD3 | protein_coding |
| ENSG00000173207 | 664.461673 | 1726.3838 | -1.3779424 | 3.10E-51 | 4.87E-49 | CKS1B | protein_coding |
| ENSG00000102870 | 1058.57375 | 341.67308 | 1.63355052 | 7.54E-51 | 1.16E-48 | ZNF629 | protein_coding |
| ENSG00000123416 | 4604.53748 | 9996.43764 | -1.1184134 | 1.20E-50 | 1.84E-48 | TUBA1B | protein_coding |
| ENSG00000137075 | 482.47288 | 1348.36693 | -1.4811701 | 1.29E-50 | 1.96E-48 | RNF38 | protein_coding |
| ENSG00000175063 | 1442.41906 | 3079.36602 | -1.0935254 | 1.81E-50 | 2.74E-48 | UBE2C | protein_coding |
| ENSG00000185551 | 990.799964 | 2157.82761 | -1.1228019 | 4.93E-50 | 7.43E-48 | NR2F2 | protein_coding |
| ENSG00000101003 | 234.486977 | 765.045545 | -1.7055374 | 5.95E-50 | 8.90E-48 | GINS1 | protein_coding |
| ENSG00000159399 | 546.351442 | 1444.13129 | -1.4016227 | 1.00E-49 | 1.47E-47 | HK2 | protein_coding |
| ENSG00000077235 | 1639.00842 | 3360.3366 | -1.0355548 | 1.95E-49 | 2.85E-47 | GTF3C1 | protein_coding |
| ENSG00000154127 | 1630.01125 | 3376.71829 | -1.0504441 | 3.68E-49 | 5.33E-47 | UBASH3B | protein_coding |
| ENSG00000066117 | 726.169528 | 1820.31833 | -1.3265692 | 1.98E-48 | 2.82E-46 | SMARCD1 | protein_coding |
| ENSG00000188747 | 601.156979 | 135.473431 | 2.15148463 | 2.71E-48 | 3.84E-46 | NOXA1 | protein_coding |
| ENSG00000104964 | 4677.98426 | 2174.86587 | 1.10543053 | 8.56E-48 | 1.20E-45 | AES | protein_coding |
| ENSG00000198826 | 1115.07763 | 2402.68771 | -1.1070982 | 1.35E-47 | 1.86E-45 | ARHGAP11A | protein_coding |
| ENSG00000211455 | 524.599379 | 1270.90718 | -1.2763742 | 1.76E-47 | 2.40E-45 | STK38L | protein_coding |
| ENSG00000075702 | 929.575041 | 1959.72729 | -1.0757557 | 2.19E-47 | 2.93E-45 | WDR62 | protein_coding |
| ENSG00000106636 | 1348.93317 | 2944.79148 | -1.1260167 | 2.23E-47 | 2.98E-45 | YKT6 | protein_coding |
| ENSG00000256463 | 390.543551 | 1160.97733 | -1.5712928 | 5.11E-47 | 6.73E-45 | SALL3 | protein_coding |
| ENSG00000140299 | 409.248442 | 1075.77106 | -1.3939932 | 7.34E-47 | 9.56E-45 | BNIP2 | protein_coding |
| ENSG00000135473 | 1350.80624 | 575.598546 | 1.2307849 | 7.39E-47 | 9.58E-45 | PAN2 | protein_coding |
| ENSG00000080986 | 921.766644 | 1962.61835 | -1.0899211 | 9.56E-47 | 1.23E-44 | NDC80 | protein_coding |
| ENSG00000124762 | 784.927568 | 2084.31127 | -1.4085404 | 9.90E-47 | 1.27E-44 | CDKN1A | protein_coding |
| ENSG00000156671 | 472.584642 | 1174.06641 | -1.3118849 | 1.04E-46 | 1.32E-44 | SAMD8 | protein_coding |
| ENSG00000204304 | 2315.96845 | 1150.96162 | 1.00955921 | 1.07E-46 | 1.35E-44 | PBX2 | protein_coding |
| ENSG00000125885 | 710.34599 | 1573.43102 | -1.1472014 | 1.91E-46 | 2.39E-44 | MCM8 | protein_coding |
| ENSG00000166147 | 1281.32166 | 522.535893 | 1.29459818 | 3.95E-46 | 4.90E-44 | FBN1 | protein_coding |
| ENSG00000104626 | 758.945669 | 1704.87081 | -1.1677071 | 6.27E-46 | 7.69E-44 | ERI1 | protein_coding |
| ENSG00000158195 | 2403.56622 | 4937.08318 | -1.0380172 | 8.75E-46 | 1.07E-43 | WASF2 | protein_coding |
| ENSG00000184208 | 956.298252 | 338.563738 | 1.49881904 | 1.15E-45 | 1.40E-43 | C22orf46 | protein_coding |
| ENSG00000110906 | 689.801513 | 1586.72602 | -1.2019946 | 5.51E-45 | 6.52E-43 | KCTD10 | protein_coding |
| ENSG00000214114 | 156.59263 | 588.154345 | -1.9090114 | 1.25E-44 | 1.48E-42 | MYCBP | protein_coding |
| ENSG00000249992 | 412.740008 | 1082.07424 | -1.390149 | 1.86E-44 | 2.17E-42 | TMEM158 | protein_coding |
| ENSG00000172667 | 310.813854 | 903.597531 | -1.5397036 | 2.54E-44 | 2.95E-42 | ZMAT3 | protein_coding |
| ENSG00000173559 | 2264.06977 | 1090.16069 | 1.054668 | 4.00E-44 | 4.59E-42 | NABP1 | protein_coding |
| ENSG00000188486 | 1172.93351 | 3012.36526 | -1.3612253 | 8.11E-44 | 9.23E-42 | H2AFX | protein_coding |
| ENSG00000006634 | 486.660362 | 1185.49551 | -1.2840793 | 1.03E-43 | 1.17E-41 | DBF4 | protein_coding |
| ENSG00000104756 | 726.000011 | 1543.38133 | -1.0881649 | 1.04E-43 | 1.17E-41 | KCTD9 | protein_coding |
| ENSG00000140525 | 713.993932 | 1651.97486 | -1.210017 | 1.48E-43 | 1.66E-41 | FANCI | protein_coding |
| ENSG00000112742 | 460.202964 | 1219.81487 | -1.4073021 | 3.73E-43 | 4.16E-41 | TTK | protein_coding |
| ENSG00000101335 | 1992.98337 | 933.060815 | 1.09565875 | 1.01E-42 | 1.12E-40 | MYL9 | protein_coding |
| ENSG00000133119 | 477.608371 | 1133.08662 | -1.2466843 | 1.39E-42 | 1.54E-40 | RFC3 | protein_coding |
| ENSG00000138778 | 662.394155 | 1559.29225 | -1.2355868 | 1.54E-42 | 1.70E-40 | CENPE | protein_coding |
| ENSG00000255248 | 1933.33285 | 924.76957 | 1.06350923 | 2.16E-42 | 2.35E-40 | MIR100HG | processed_transcript |
| ENSG00000165617 | 1008.65063 | 357.142308 | 1.4992177 | 2.51E-42 | 2.72E-40 | DACT1 | protein_coding |
| ENSG00000166851 | 1688.83924 | 3583.16216 | -1.0853215 | 3.05E-42 | 3.28E-40 | PLK1 | protein_coding |
| ENSG00000108100 | 907.14555 | 1853.73226 | -1.03029 | 7.05E-42 | 7.41E-40 | CCNY | protein_coding |
| ENSG00000171208 | 813.028052 | 1801.01508 | -1.1469828 | 2.07E-41 | 2.14E-39 | NETO2 | protein_coding |
| ENSG00000137812 | 801.223799 | 1699.42031 | -1.0849676 | 2.20E-41 | 2.27E-39 | KNL1 | protein_coding |
| ENSG00000108179 | 1137.21207 | 2303.9109 | -1.0183262 | 3.21E-41 | 3.30E-39 | PPIF | protein_coding |
| ENSG00000259781 | 323.130524 | 854.9312 | -1.404138 | 4.24E-41 | 4.30E-39 | HMGB1P6 | processed_pseudogene |
| ENSG00000071537 | 842.687624 | 1747.80125 | -1.051853 | 5.37E-41 | 5.43E-39 | SEL1L | protein_coding |
| ENSG00000089486 | 159.425422 | 616.776666 | -1.9531234 | 9.30E-41 | 9.31E-39 | CDIP1 | protein_coding |
| ENSG00000185100 | 1004.70993 | 351.097862 | 1.51717989 | 1.01E-40 | 1.01E-38 | ADSSL1 | protein_coding |
| ENSG00000184203 | 596.388313 | 1341.47553 | -1.1687124 | 1.25E-40 | 1.24E-38 | PPP1R2 | protein_coding |
| ENSG00000129195 | 274.08116 | 795.2522 | -1.5365396 | 1.32E-40 | 1.29E-38 | PIMREG | protein_coding |
| ENSG00000275216 | 127.65802 | 531.674934 | -2.0613955 | 1.82E-40 | 1.77E-38 | AL161431.1 | lincRNA |
| ENSG00000136826 | 964.281819 | 381.292783 | 1.3392388 | 3.28E-40 | 3.12E-38 | KLF4 | protein_coding |
| ENSG00000126787 | 760.827617 | 1589.51641 | -1.0623932 | 5.51E-40 | 5.23E-38 | DLGAP5 | protein_coding |
| ENSG00000196381 | 347.738898 | 949.503578 | -1.449258 | 6.02E-40 | 5.69E-38 | ZNF781 | protein_coding |
| ENSG00000073111 | 1148.90434 | 2604.74695 | -1.1801174 | 8.30E-40 | 7.81E-38 | MCM2 | protein_coding |
| ENSG00000178951 | 2028.57763 | 991.080881 | 1.03440372 | 1.74E-39 | 1.61E-37 | ZBTB7A | protein_coding |
| ENSG00000069702 | 319.446687 | 863.698964 | -1.4343199 | 2.78E-39 | 2.55E-37 | TGFBR3 | protein_coding |
| ENSG00000100360 | 743.741365 | 249.111366 | 1.57875259 | 5.00E-39 | 4.57E-37 | IFT27 | protein_coding |
| ENSG00000168496 | 966.526109 | 2042.77261 | -1.0793253 | 1.21E-38 | 1.10E-36 | FEN1 | protein_coding |
| ENSG00000174938 | 1995.04448 | 982.467972 | 1.0225391 | 1.22E-38 | 1.10E-36 | SEZ6L2 | protein_coding |
| ENSG00000070950 | 293.032032 | 831.889706 | -1.5040739 | 1.57E-38 | 1.40E-36 | RAD18 | protein_coding |
| ENSG00000172893 | 429.553459 | 1073.67293 | -1.3208527 | 1.99E-38 | 1.78E-36 | DHCR7 | protein_coding |
| ENSG00000196083 | 545.885989 | 1228.59592 | -1.1705088 | 2.23E-38 | 1.99E-36 | IL1RAP | protein_coding |
| ENSG00000085982 | 1310.0717 | 559.592421 | 1.22821166 | 2.67E-38 | 2.36E-36 | USP40 | protein_coding |
| ENSG00000184014 | 1498.3492 | 707.933621 | 1.08247648 | 3.77E-38 | 3.32E-36 | DENND5A | protein_coding |
| ENSG00000101447 | 1027.52302 | 2131.49665 | -1.0527065 | 4.55E-38 | 3.99E-36 | FAM83D | protein_coding |
| ENSG00000132635 | 898.072785 | 352.766238 | 1.34926157 | 4.95E-38 | 4.31E-36 | PCED1A | protein_coding |
| ENSG00000102098 | 255.896036 | 757.11 | -1.5656005 | 7.63E-38 | 6.57E-36 | SCML2 | protein_coding |
| ENSG00000153989 | 1011.2017 | 2049.56464 | -1.0190649 | 1.22E-37 | 1.04E-35 | NUS1 | protein_coding |
| ENSG00000119969 | 728.704022 | 1646.86838 | -1.1768711 | 1.27E-37 | 1.08E-35 | HELLS | protein_coding |
| ENSG00000149809 | 900.321441 | 299.859908 | 1.58760647 | 1.42E-37 | 1.20E-35 | TM7SF2 | protein_coding |
| ENSG00000124098 | 982.62972 | 410.505811 | 1.25897966 | 2.44E-37 | 2.04E-35 | FAM210B | protein_coding |
| ENSG00000169299 | 892.698727 | 1799.62415 | -1.011568 | 2.66E-37 | 2.21E-35 | PGM2 | protein_coding |
| ENSG00000137713 | 696.018772 | 1439.96441 | -1.0484608 | 2.98E-37 | 2.46E-35 | PPP2R1B | protein_coding |
| ENSG00000070882 | 602.243977 | 1288.04989 | -1.0964231 | 3.97E-37 | 3.26E-35 | OSBPL3 | protein_coding |
| ENSG00000197265 | 390.548132 | 1027.55158 | -1.39482 | 4.25E-37 | 3.47E-35 | GTF2E2 | protein_coding |
| ENSG00000180921 | 691.798853 | 233.227636 | 1.56957314 | 6.93E-37 | 5.56E-35 | FAM83H | protein_coding |
| ENSG00000143228 | 610.692626 | 1316.2655 | -1.1084909 | 8.68E-37 | 6.86E-35 | NUF2 | protein_coding |
| ENSG00000116133 | 1155.00649 | 2351.59159 | -1.0254066 | 9.25E-37 | 7.29E-35 | DHCR24 | protein_coding |
| ENSG00000110047 | 1251.91893 | 2747.65409 | -1.1337861 | 1.60E-36 | 1.25E-34 | EHD1 | protein_coding |
| ENSG00000139354 | 584.9583 | 1242.50954 | -1.0870924 | 2.15E-36 | 1.66E-34 | GAS2L3 | protein_coding |
| ENSG00000171703 | 1552.50172 | 689.754608 | 1.17087551 | 2.26E-36 | 1.74E-34 | TCEA2 | protein_coding |
| ENSG00000145293 | 437.182963 | 1053.08612 | -1.2671172 | 2.37E-36 | 1.82E-34 | ENOPH1 | protein_coding |
| ENSG00000167123 | 1654.30885 | 724.313037 | 1.19190371 | 2.48E-36 | 1.90E-34 | CERCAM | protein_coding |
| ENSG00000057704 | 260.044886 | 730.056412 | -1.4893161 | 2.50E-36 | 1.91E-34 | TMCC3 | protein_coding |
| ENSG00000054965 | 759.992237 | 1527.73644 | -1.0073101 | 2.57E-36 | 1.95E-34 | FAM168A | protein_coding |
| ENSG00000126458 | 2254.27363 | 1085.00202 | 1.05601716 | 3.24E-36 | 2.46E-34 | RRAS | protein_coding |
| ENSG00000166734 | 666.624189 | 1399.80289 | -1.0699727 | 3.75E-36 | 2.83E-34 | CASC4 | protein_coding |
| ENSG00000155959 | 721.556435 | 1477.55371 | -1.0335107 | 3.86E-36 | 2.90E-34 | VBP1 | protein_coding |
| ENSG00000060491 | 1570.27529 | 733.726617 | 1.09855736 | 4.04E-36 | 3.02E-34 | OGFR | protein_coding |
| ENSG00000188070 | 1210.3654 | 548.931457 | 1.14121859 | 5.20E-36 | 3.86E-34 | C11orf95 | protein_coding |
| ENSG00000182010 | 236.878206 | 723.739281 | -1.6096628 | 5.32E-36 | 3.94E-34 | RTKN2 | protein_coding |
| ENSG00000112029 | 487.989238 | 1108.49964 | -1.183509 | 6.18E-36 | 4.56E-34 | FBXO5 | protein_coding |
| ENSG00000135678 | 413.73718 | 969.71229 | -1.2291588 | 7.49E-36 | 5.51E-34 | CPM | protein_coding |
| ENSG00000189057 | 312.848431 | 800.687785 | -1.3565229 | 8.80E-36 | 6.46E-34 | FAM111B | protein_coding |
| ENSG00000166073 | 406.250641 | 1050.877 | -1.3713094 | 9.48E-36 | 6.93E-34 | GPR176 | protein_coding |
| ENSG00000101187 | 315.796301 | 862.349701 | -1.4483268 | 1.06E-35 | 7.73E-34 | SLCO4A1 | protein_coding |
| ENSG00000163535 | 412.662892 | 960.858377 | -1.2192849 | 1.76E-35 | 1.27E-33 | SGO2 | protein_coding |
| ENSG00000106086 | 466.165523 | 1091.92321 | -1.2280409 | 2.17E-35 | 1.56E-33 | PLEKHA8 | protein_coding |
| ENSG00000259330 | 218.883312 | 710.630198 | -1.6982132 | 4.95E-35 | 3.50E-33 | INAFM2 | protein_coding |
| ENSG00000155254 | 1261.25395 | 536.436118 | 1.23390321 | 5.99E-35 | 4.21E-33 | MARVELD1 | protein_coding |
| ENSG00000130956 | 1042.63394 | 432.120346 | 1.27186291 | 1.46E-34 | 1.01E-32 | HABP4 | protein_coding |
| ENSG00000267041 | 396.351265 | 957.867808 | -1.2717982 | 1.80E-34 | 1.25E-32 | ZNF850 | protein_coding |
| ENSG00000168283 | 647.209033 | 1380.91984 | -1.0933968 | 1.98E-34 | 1.36E-32 | BMI1 | protein_coding |
| ENSG00000151725 | 376.039545 | 899.341165 | -1.2578531 | 4.57E-34 | 3.08E-32 | CENPU | protein_coding |
| ENSG00000275395 | 561.053878 | 184.201217 | 1.6055136 | 5.85E-34 | 3.91E-32 | FCGBP | protein_coding |
| ENSG00000182287 | 642.592634 | 1299.53102 | -1.0154449 | 5.85E-34 | 3.91E-32 | AP1S2 | protein_coding |
| ENSG00000164109 | 563.349407 | 1252.04944 | -1.1518537 | 7.09E-34 | 4.71E-32 | MAD2L1 | protein_coding |
| ENSG00000141499 | 571.748851 | 1269.40767 | -1.1503355 | 1.07E-33 | 7.04E-32 | WRAP53 | protein_coding |
| ENSG00000078070 | 829.17117 | 342.277611 | 1.27619504 | 1.37E-33 | 8.96E-32 | MCCC1 | protein_coding |
| ENSG00000160209 | 977.14401 | 1961.37579 | -1.0044561 | 1.79E-33 | 1.16E-31 | PDXK | protein_coding |
| ENSG00000075975 | 254.586593 | 697.595778 | -1.4549577 | 2.02E-33 | 1.31E-31 | MKRN2 | protein_coding |
| ENSG00000169231 | 1264.45949 | 568.138551 | 1.15263606 | 2.03E-33 | 1.31E-31 | THBS3 | protein_coding |
| ENSG00000078018 | 477.560578 | 139.692995 | 1.77193541 | 2.29E-33 | 1.46E-31 | MAP2 | protein_coding |
| ENSG00000197147 | 216.211176 | 659.778503 | -1.6103376 | 2.56E-33 | 1.63E-31 | LRRC8B | protein_coding |
| ENSG00000136982 | 361.989182 | 886.725704 | -1.2917473 | 3.66E-33 | 2.29E-31 | DSCC1 | protein_coding |
| ENSG00000178719 | 873.474959 | 1947.81152 | -1.1570382 | 1.43E-32 | 8.79E-31 | GRINA | protein_coding |
| ENSG00000163291 | 428.021642 | 954.442843 | -1.1565121 | 1.63E-32 | 9.93E-31 | PAQR3 | protein_coding |
| ENSG00000105270 | 718.363987 | 280.51526 | 1.35709315 | 2.47E-32 | 1.50E-30 | CLIP3 | protein_coding |
| ENSG00000158710 | 805.678502 | 1719.46569 | -1.0927043 | 3.23E-32 | 1.96E-30 | TAGLN2 | protein_coding |
| ENSG00000111665 | 608.098515 | 1239.66534 | -1.026761 | 4.83E-32 | 2.92E-30 | CDCA3 | protein_coding |
| ENSG00000063180 | 520.801803 | 166.260751 | 1.64820752 | 5.76E-32 | 3.46E-30 | CA11 | protein_coding |
| ENSG00000128510 | 1113.39074 | 516.904358 | 1.10587324 | 8.24E-32 | 4.93E-30 | CPA4 | protein_coding |
| ENSG00000154839 | 225.088326 | 635.618213 | -1.4962053 | 1.07E-31 | 6.40E-30 | SKA1 | protein_coding |
| ENSG00000117650 | 370.624309 | 840.218216 | -1.1809909 | 1.78E-31 | 1.05E-29 | NEK2 | protein_coding |
| ENSG00000127564 | 563.83852 | 1138.63214 | -1.0139559 | 1.81E-31 | 1.06E-29 | PKMYT1 | protein_coding |
| ENSG00000165997 | 574.757936 | 1254.76974 | -1.1253478 | 3.97E-31 | 2.31E-29 | ARL5B | protein_coding |
| ENSG00000144677 | 461.249548 | 986.65369 | -1.0968389 | 4.03E-31 | 2.34E-29 | CTDSPL | protein_coding |
| ENSG00000146476 | 589.317634 | 1248.83508 | -1.0841069 | 5.12E-31 | 2.93E-29 | ARMT1 | protein_coding |
| ENSG00000169607 | 460.585933 | 990.501964 | -1.1050022 | 5.71E-31 | 3.25E-29 | CKAP2L | protein_coding |
| ENSG00000142459 | 1160.5879 | 524.419381 | 1.14650649 | 7.12E-31 | 4.03E-29 | EVI5L | protein_coding |
| ENSG00000152127 | 518.1022 | 1134.39589 | -1.1296916 | 9.74E-31 | 5.47E-29 | MGAT5 | protein_coding |
| ENSG00000075218 | 731.774342 | 1488.54941 | -1.0238794 | 1.67E-30 | 9.33E-29 | GTSE1 | protein_coding |
| ENSG00000065328 | 447.409867 | 1029.13675 | -1.2007567 | 1.91E-30 | 1.06E-28 | MCM10 | protein_coding |
| ENSG00000156531 | 622.794776 | 1255.16723 | -1.0109837 | 3.00E-30 | 1.64E-28 | PHF6 | protein_coding |
| ENSG00000100749 | 618.429285 | 1239.75411 | -1.0028816 | 4.59E-30 | 2.46E-28 | VRK1 | protein_coding |
| ENSG00000169550 | 1248.02411 | 526.050649 | 1.24535646 | 5.50E-30 | 2.95E-28 | MUC15 | protein_coding |
| ENSG00000120437 | 476.48397 | 1034.57564 | -1.1191861 | 6.21E-30 | 3.31E-28 | ACAT2 | protein_coding |
| ENSG00000100767 | 869.540247 | 373.331808 | 1.2185223 | 1.04E-29 | 5.51E-28 | PAPLN | protein_coding |
| ENSG00000148356 | 1616.43417 | 787.910978 | 1.03715357 | 1.26E-29 | 6.69E-28 | LRSAM1 | protein_coding |
| ENSG00000272068 | 268.703871 | 42.4891553 | 2.66603542 | 1.47E-29 | 7.75E-28 | AL365181.2 | lincRNA |
| ENSG00000126524 | 508.356121 | 1103.78683 | -1.1196211 | 1.93E-29 | 1.02E-27 | SBDS | protein_coding |
| ENSG00000152256 | 442.648626 | 994.930764 | -1.1683468 | 2.21E-29 | 1.16E-27 | PDK1 | protein_coding |
| ENSG00000100441 | 1101.76929 | 478.385385 | 1.20447921 | 4.16E-29 | 2.17E-27 | KHNYN | protein_coding |
| ENSG00000247315 | 1282.13011 | 589.098112 | 1.12300389 | 4.32E-29 | 2.25E-27 | ZCCHC3 | protein_coding |
| ENSG00000116984 | 435.113513 | 991.905336 | -1.1880534 | 4.77E-29 | 2.47E-27 | MTR | protein_coding |
| ENSG00000174028 | 505.125587 | 162.019759 | 1.64267654 | 6.86E-29 | 3.53E-27 | FAM3C2 | processed_pseudogene |
| ENSG00000035499 | 309.872217 | 772.229944 | -1.3178269 | 6.97E-29 | 3.58E-27 | DEPDC1B | protein_coding |
| ENSG00000111077 | 885.639355 | 410.922522 | 1.10817828 | 7.52E-29 | 3.84E-27 | TNS2 | protein_coding |
| ENSG00000101868 | 592.183747 | 1232.93358 | -1.0567431 | 7.74E-29 | 3.94E-27 | POLA1 | protein_coding |
| ENSG00000147475 | 1039.67599 | 508.434627 | 1.03252322 | 7.75E-29 | 3.94E-27 | ERLIN2 | protein_coding |
| ENSG00000174371 | 287.43042 | 792.418411 | -1.4626567 | 1.55E-28 | 7.82E-27 | EXO1 | protein_coding |
| ENSG00000254858 | 162.548133 | 524.816088 | -1.6909692 | 1.80E-28 | 9.05E-27 | MPV17L2 | protein_coding |
| ENSG00000215375 | 436.988276 | 124.725386 | 1.80901861 | 2.46E-28 | 1.23E-26 | MYL5 | protein_coding |
| ENSG00000197299 | 154.792664 | 466.321827 | -1.5897111 | 3.89E-28 | 1.93E-26 | BLM | protein_coding |
| ENSG00000148943 | 223.035607 | 611.625613 | -1.4536702 | 5.01E-28 | 2.48E-26 | LIN7C | protein_coding |
| ENSG00000118193 | 482.485818 | 1114.32244 | -1.2069507 | 6.72E-28 | 3.31E-26 | KIF14 | protein_coding |
| ENSG00000132510 | 1313.14178 | 634.235472 | 1.0513236 | 7.56E-28 | 3.71E-26 | KDM6B | protein_coding |
| ENSG00000164305 | 462.424817 | 992.685966 | -1.1023532 | 8.68E-28 | 4.24E-26 | CASP3 | protein_coding |
| ENSG00000186871 | 279.71587 | 664.881804 | -1.249101 | 1.03E-27 | 5.04E-26 | ERCC6L | protein_coding |
| ENSG00000126391 | 578.502481 | 1170.00671 | -1.0161568 | 1.36E-27 | 6.60E-26 | FRMD8 | protein_coding |
| ENSG00000127328 | 410.416324 | 873.933592 | -1.0897195 | 1.51E-27 | 7.28E-26 | RAB3IP | protein_coding |
| ENSG00000115540 | 349.04279 | 828.635407 | -1.2471892 | 2.37E-27 | 1.13E-25 | MOB4 | protein_coding |
| ENSG00000134758 | 290.921631 | 690.974681 | -1.2485666 | 3.64E-27 | 1.74E-25 | RNF138 | protein_coding |
| ENSG00000144824 | 427.453727 | 919.606708 | -1.1042716 | 3.91E-27 | 1.86E-25 | PHLDB2 | protein_coding |
| ENSG00000105341 | 487.29657 | 1073.00324 | -1.1385017 | 5.06E-27 | 2.39E-25 | DMAC2 | protein_coding |
| ENSG00000111247 | 443.357133 | 1043.56976 | -1.235923 | 8.38E-27 | 3.94E-25 | RAD51AP1 | protein_coding |
| ENSG00000163507 | 427.992519 | 1011.89184 | -1.2405195 | 9.53E-27 | 4.45E-25 | CIP2A | protein_coding |
| ENSG00000184939 | 952.036305 | 452.435882 | 1.07244902 | 1.11E-26 | 5.17E-25 | ZFP90 | protein_coding |
| ENSG00000165490 | 157.978479 | 476.902803 | -1.5953106 | 2.85E-26 | 1.31E-24 | DDIAS | protein_coding |
| ENSG00000152518 | 554.876055 | 1128.05771 | -1.0242056 | 3.94E-26 | 1.80E-24 | ZFP36L2 | protein_coding |
| ENSG00000175938 | 440.340063 | 137.727778 | 1.67887506 | 4.62E-26 | 2.09E-24 | ORAI3 | protein_coding |
| ENSG00000145882 | 597.11325 | 225.067519 | 1.40724603 | 4.71E-26 | 2.13E-24 | PCYOX1L | protein_coding |
| ENSG00000167637 | 227.51255 | 595.541672 | -1.3879843 | 4.84E-26 | 2.19E-24 | ZNF283 | protein_coding |
| ENSG00000176087 | 817.983708 | 1659.05853 | -1.0199033 | 4.97E-26 | 2.23E-24 | SLC35A4 | protein_coding |
| ENSG00000161692 | 295.179724 | 688.28426 | -1.2217673 | 5.37E-26 | 2.41E-24 | DBF4B | protein_coding |
| ENSG00000145242 | 231.050617 | 36.4294165 | 2.66650206 | 6.74E-26 | 2.98E-24 | EPHA5 | protein_coding |
| ENSG00000130313 | 1892.11241 | 865.703954 | 1.12842853 | 7.61E-26 | 3.36E-24 | PGLS | protein_coding |
| ENSG00000161677 | 568.67641 | 221.250203 | 1.36201311 | 9.09E-26 | 4.01E-24 | JOSD2 | protein_coding |
| ENSG00000146410 | 201.493587 | 558.023835 | -1.4710507 | 9.55E-26 | 4.20E-24 | MTFR2 | protein_coding |
| ENSG00000205208 | 285.414307 | 658.570151 | -1.2068708 | 1.31E-25 | 5.72E-24 | C4orf46 | protein_coding |
| ENSG00000119953 | 473.932787 | 977.879765 | -1.04584 | 1.47E-25 | 6.42E-24 | SMNDC1 | protein_coding |
| ENSG00000128656 | 132.798421 | 428.610573 | -1.6896682 | 1.49E-25 | 6.47E-24 | CHN1 | protein_coding |
| ENSG00000051341 | 386.370584 | 816.234152 | -1.0793619 | 2.69E-25 | 1.15E-23 | POLQ | protein_coding |
| ENSG00000164045 | 418.744385 | 916.211929 | -1.1283021 | 2.97E-25 | 1.27E-23 | CDC25A | protein_coding |
| ENSG00000114853 | 497.145891 | 188.427392 | 1.3988296 | 3.83E-25 | 1.64E-23 | ZBTB47 | protein_coding |
| ENSG00000261609 | 256.472499 | 612.185052 | -1.2549929 | 3.88E-25 | 1.66E-23 | GAN | protein_coding |
| ENSG00000142156 | 543.079247 | 212.44981 | 1.35303507 | 4.14E-25 | 1.76E-23 | COL6A1 | protein_coding |
| ENSG00000172830 | 798.687138 | 361.194018 | 1.14567574 | 4.75E-25 | 2.00E-23 | SSH3 | protein_coding |
| ENSG00000240225 | 583.400875 | 1184.03563 | -1.0211197 | 4.93E-25 | 2.07E-23 | ZNF542P | transcribed_unprocessed_pseudogene |
| ENSG00000142731 | 390.711974 | 823.217015 | -1.0745331 | 5.72E-25 | 2.40E-23 | PLK4 | protein_coding |
| ENSG00000197283 | 760.501629 | 343.465713 | 1.14623438 | 5.97E-25 | 2.50E-23 | SYNGAP1 | protein_coding |
| ENSG00000119711 | 687.718345 | 294.57681 | 1.2227046 | 6.86E-25 | 2.86E-23 | ALDH6A1 | protein_coding |
| ENSG00000104147 | 100.765061 | 339.654223 | -1.7529053 | 1.04E-24 | 4.30E-23 | OIP5 | protein_coding |
| ENSG00000154319 | 249.783749 | 641.299982 | -1.3591355 | 1.24E-24 | 5.08E-23 | FAM167A | protein_coding |
| ENSG00000181444 | 501.165477 | 179.992399 | 1.47687934 | 1.29E-24 | 5.29E-23 | ZNF467 | protein_coding |
| ENSG00000172831 | 1009.43553 | 495.926972 | 1.02619157 | 1.63E-24 | 6.63E-23 | CES2 | protein_coding |
| ENSG00000006282 | 501.498279 | 198.357263 | 1.33849529 | 2.76E-24 | 1.11E-22 | SPATA20 | protein_coding |
| ENSG00000178295 | 330.72712 | 755.322643 | -1.1905598 | 3.76E-24 | 1.49E-22 | GEN1 | protein_coding |
| ENSG00000074527 | 612.760214 | 236.855131 | 1.37218741 | 4.66E-24 | 1.85E-22 | NTN4 | protein_coding |
| ENSG00000167535 | 716.65823 | 329.811514 | 1.11884484 | 5.39E-24 | 2.13E-22 | CACNB3 | protein_coding |
| ENSG00000176225 | 248.322093 | 594.672891 | -1.2600969 | 7.31E-24 | 2.89E-22 | RTTN | protein_coding |
| ENSG00000164615 | 943.881372 | 465.508549 | 1.02034688 | 7.33E-24 | 2.89E-22 | CAMLG | protein_coding |
| ENSG00000145332 | 323.1469 | 708.207091 | -1.1323282 | 7.67E-24 | 3.02E-22 | KLHL8 | protein_coding |
| ENSG00000146376 | 337.611651 | 718.900977 | -1.0903367 | 8.37E-24 | 3.28E-22 | ARHGAP18 | protein_coding |
| ENSG00000109436 | 838.951387 | 400.384403 | 1.06774033 | 8.96E-24 | 3.51E-22 | TBC1D9 | protein_coding |
| ENSG00000143622 | 757.611511 | 357.039651 | 1.0863934 | 9.00E-24 | 3.52E-22 | RIT1 | protein_coding |
| ENSG00000103044 | 705.707871 | 318.10075 | 1.14926661 | 9.91E-24 | 3.87E-22 | HAS3 | protein_coding |
| ENSG00000111271 | 733.464962 | 336.733524 | 1.12173127 | 1.02E-23 | 3.98E-22 | ACAD10 | protein_coding |
| ENSG00000246705 | 1016.81166 | 487.442139 | 1.06190685 | 1.12E-23 | 4.34E-22 | H2AFJ | protein_coding |
| ENSG00000152078 | 383.615964 | 797.461854 | -1.0559019 | 1.14E-23 | 4.42E-22 | TMEM56 | protein_coding |
| ENSG00000181744 | 260.577141 | 603.729993 | -1.2124751 | 1.33E-23 | 5.12E-22 | C3orf58 | protein_coding |
| ENSG00000127415 | 482.680232 | 164.585226 | 1.55031228 | 1.69E-23 | 6.46E-22 | IDUA | protein_coding |
| ENSG00000270696 | 92.9556248 | 322.15256 | -1.7909384 | 2.14E-23 | 8.13E-22 | AC005034.3 | antisense |
| ENSG00000114023 | 403.498567 | 829.421546 | -1.0400825 | 3.14E-23 | 1.18E-21 | FAM162A | protein_coding |
| ENSG00000153936 | 464.957615 | 945.98793 | -1.02338 | 3.20E-23 | 1.20E-21 | HS2ST1 | protein_coding |
| ENSG00000231607 | 73.1500253 | 285.556467 | -1.9672243 | 3.84E-23 | 1.44E-21 | DLEU2 | antisense |
| ENSG00000171365 | 402.112563 | 849.731725 | -1.0784443 | 4.64E-23 | 1.73E-21 | CLCN5 | protein_coding |
| ENSG00000186106 | 374.899479 | 782.86159 | -1.0625393 | 6.10E-23 | 2.26E-21 | ANKRD46 | protein_coding |
| ENSG00000239779 | 395.244989 | 137.734598 | 1.52196386 | 6.61E-23 | 2.45E-21 | WBP1 | protein_coding |
| ENSG00000166436 | 829.038087 | 340.188579 | 1.28339521 | 6.65E-23 | 2.45E-21 | TRIM66 | protein_coding |
| ENSG00000150281 | 219.395052 | 38.6620878 | 2.4965364 | 7.22E-23 | 2.65E-21 | CTF1 | protein_coding |
| ENSG00000198743 | 326.736034 | 731.62589 | -1.161792 | 8.08E-23 | 2.96E-21 | SLC5A3 | protein_coding |
| ENSG00000159792 | 859.091662 | 415.923412 | 1.04736695 | 8.90E-23 | 3.24E-21 | PSKH1 | protein_coding |
| ENSG00000179406 | 583.555791 | 213.455987 | 1.44776782 | 9.57E-23 | 3.47E-21 | LINC00174 | processed_transcript |
| ENSG00000136943 | 110.189067 | 369.940169 | -1.7491781 | 9.57E-23 | 3.47E-21 | CTSV | protein_coding |
| ENSG00000142235 | 286.754041 | 72.8155207 | 1.98070815 | 1.64E-22 | 5.90E-21 | LMTK3 | protein_coding |
| ENSG00000136492 | 226.46484 | 532.899072 | -1.2342084 | 2.09E-22 | 7.48E-21 | BRIP1 | protein_coding |
| ENSG00000178105 | 245.477846 | 568.038552 | -1.2094375 | 2.47E-22 | 8.83E-21 | DDX10 | protein_coding |
| ENSG00000187231 | 305.435326 | 661.078311 | -1.1136326 | 2.49E-22 | 8.90E-21 | SESTD1 | protein_coding |
| ENSG00000118363 | 344.446898 | 709.72001 | -1.0427407 | 3.70E-22 | 1.31E-20 | SPCS2 | protein_coding |
| ENSG00000185305 | 52.0015471 | 222.585289 | -2.0979408 | 3.81E-22 | 1.34E-20 | ARL15 | protein_coding |
| ENSG00000165195 | 197.575762 | 498.539222 | -1.3332573 | 4.04E-22 | 1.42E-20 | PIGA | protein_coding |
| ENSG00000110492 | 2264.94414 | 1090.89874 | 1.05482276 | 4.35E-22 | 1.53E-20 | MDK | protein_coding |
| ENSG00000105327 | 374.522952 | 121.464223 | 1.62733497 | 5.44E-22 | 1.90E-20 | BBC3 | protein_coding |
| ENSG00000254245 | 647.904871 | 298.142002 | 1.12103777 | 7.78E-22 | 2.70E-20 | PCDHGA3 | protein_coding |
| ENSG00000180537 | 159.985509 | 427.637317 | -1.4187098 | 8.38E-22 | 2.89E-20 | RNF182 | protein_coding |
| ENSG00000196705 | 402.639284 | 833.258498 | -1.0485595 | 8.67E-22 | 2.99E-20 | ZNF431 | protein_coding |
| ENSG00000224032 | 606.935837 | 267.363466 | 1.18401415 | 9.12E-22 | 3.14E-20 | EPB41L4A-AS1 | lincRNA |
| ENSG00000077238 | 445.370606 | 910.744918 | -1.0308744 | 9.76E-22 | 3.35E-20 | IL4R | protein_coding |
| ENSG00000213918 | 652.047901 | 304.594877 | 1.09770117 | 1.30E-21 | 4.42E-20 | DNASE1 | protein_coding |
| ENSG00000123219 | 198.691355 | 543.837021 | -1.4504824 | 1.47E-21 | 5.00E-20 | CENPK | protein_coding |
| ENSG00000108960 | 85.110346 | 298.011698 | -1.8074896 | 1.60E-21 | 5.41E-20 | MMD | protein_coding |
| ENSG00000041982 | 423.42303 | 161.296993 | 1.39326021 | 1.64E-21 | 5.53E-20 | TNC | protein_coding |
| ENSG00000240875 | 131.121345 | 12.8742338 | 3.34506703 | 1.80E-21 | 6.07E-20 | LINC00886 | lincRNA |
| ENSG00000197558 | 222.380089 | 38.6244174 | 2.51829869 | 2.23E-21 | 7.50E-20 | SSPO | protein_coding |
| ENSG00000132967 | 277.188362 | 654.122203 | -1.2387614 | 2.42E-21 | 8.11E-20 | HMGB1P5 | transcribed_processed_pseudogene |
| ENSG00000198478 | 260.951918 | 590.387346 | -1.1770268 | 3.19E-21 | 1.06E-19 | SH3BGRL2 | protein_coding |
| ENSG00000174442 | 466.029529 | 936.789239 | -1.0071504 | 3.47E-21 | 1.15E-19 | ZWILCH | protein_coding |
| ENSG00000161671 | 391.67935 | 907.198013 | -1.2131418 | 3.96E-21 | 1.31E-19 | EMC10 | protein_coding |
| ENSG00000223745 | 467.828795 | 141.088503 | 1.72859549 | 4.38E-21 | 1.44E-19 | CCDC18-AS1 | processed_transcript |
| ENSG00000136367 | 277.661912 | 81.4533723 | 1.77040212 | 5.38E-21 | 1.76E-19 | ZFHX2 | protein_coding |
| ENSG00000100577 | 338.708748 | 114.217478 | 1.56967578 | 1.02E-20 | 3.26E-19 | GSTZ1 | protein_coding |
| ENSG00000114268 | 224.677196 | 533.509651 | -1.2459495 | 1.03E-20 | 3.29E-19 | PFKFB4 | protein_coding |
| ENSG00000138439 | 144.535926 | 415.076953 | -1.5207068 | 1.07E-20 | 3.43E-19 | FAM117B | protein_coding |
| ENSG00000125864 | 274.180132 | 75.465441 | 1.86206144 | 1.16E-20 | 3.71E-19 | BFSP1 | protein_coding |
| ENSG00000272556 | 868.040454 | 426.540431 | 1.02351158 | 2.15E-20 | 6.72E-19 | GTF2IP13 | transcribed_unprocessed_pseudogene |
| ENSG00000140465 | 269.938043 | 77.7285702 | 1.79539645 | 2.29E-20 | 7.13E-19 | CYP1A1 | protein_coding |
| ENSG00000131153 | 356.771682 | 755.205477 | -1.0819203 | 2.92E-20 | 9.04E-19 | GINS2 | protein_coding |
| ENSG00000197757 | 671.953825 | 325.621623 | 1.04552828 | 3.13E-20 | 9.64E-19 | HOXC6 | protein_coding |
| ENSG00000172687 | 226.817249 | 518.534069 | -1.1916984 | 3.48E-20 | 1.07E-18 | ZNF738 | protein_coding |
| ENSG00000163808 | 342.879593 | 694.330441 | -1.0179545 | 3.59E-20 | 1.10E-18 | KIF15 | protein_coding |
| ENSG00000226985 | 899.266455 | 415.073381 | 1.11364602 | 4.53E-20 | 1.38E-18 | LINC01203 | lincRNA |
| ENSG00000185453 | 435.698853 | 177.932914 | 1.29329701 | 6.81E-20 | 2.06E-18 | ZSWIM9 | protein_coding |
| ENSG00000225138 | 230.132215 | 58.3634621 | 1.97786418 | 7.09E-20 | 2.13E-18 | SLC9A3-AS1 | processed_transcript |
| ENSG00000171320 | 202.310652 | 492.986434 | -1.2841604 | 7.86E-20 | 2.35E-18 | ESCO2 | protein_coding |
| ENSG00000181649 | 297.121165 | 658.483811 | -1.1483298 | 9.47E-20 | 2.82E-18 | PHLDA2 | protein_coding |
| ENSG00000166401 | 181.686861 | 452.633948 | -1.3172603 | 1.24E-19 | 3.68E-18 | SERPINB8 | protein_coding |
| ENSG00000021762 | 383.615878 | 138.380661 | 1.46992757 | 1.27E-19 | 3.76E-18 | OSBPL5 | protein_coding |
| ENSG00000104267 | 142.825641 | 388.041506 | -1.4443352 | 2.12E-19 | 6.19E-18 | CA2 | protein_coding |
| ENSG00000094804 | 289.932027 | 620.422182 | -1.0965849 | 2.16E-19 | 6.29E-18 | CDC6 | protein_coding |
| ENSG00000113739 | 648.465399 | 312.406412 | 1.05443609 | 2.30E-19 | 6.66E-18 | STC2 | protein_coding |
| ENSG00000146143 | 333.677537 | 676.740008 | -1.0192743 | 2.64E-19 | 7.64E-18 | PRIM2 | protein_coding |
| ENSG00000159167 | 73.3859606 | 258.914194 | -1.81977 | 2.89E-19 | 8.31E-18 | STC1 | protein_coding |
| ENSG00000169379 | 224.618561 | 503.028266 | -1.1627859 | 4.15E-19 | 1.19E-17 | ARL13B | protein_coding |
| ENSG00000117305 | 843.919192 | 415.97811 | 1.02093799 | 6.63E-19 | 1.88E-17 | HMGCL | protein_coding |
| ENSG00000125149 | 329.759245 | 678.42303 | -1.0401938 | 9.03E-19 | 2.54E-17 | C16orf70 | protein_coding |
| ENSG00000235173 | 424.822155 | 860.115459 | -1.0174237 | 1.32E-18 | 3.66E-17 | HGH1 | protein_coding |
| ENSG00000172167 | 227.494849 | 497.392925 | -1.1285563 | 1.53E-18 | 4.19E-17 | MTBP | protein_coding |
| ENSG00000184381 | 341.413712 | 126.241117 | 1.43511454 | 2.11E-18 | 5.72E-17 | PLA2G6 | protein_coding |
| ENSG00000197780 | 246.216628 | 530.031558 | -1.106631 | 2.68E-18 | 7.21E-17 | TAF13 | protein_coding |
| ENSG00000087076 | 490.661988 | 204.019465 | 1.26786156 | 2.99E-18 | 8.02E-17 | HSD17B14 | protein_coding |
| ENSG00000105379 | 781.396117 | 372.064433 | 1.07234162 | 3.76E-18 | 1.00E-16 | ETFB | protein_coding |
| ENSG00000196584 | 308.006315 | 632.537265 | -1.0384728 | 4.12E-18 | 1.10E-16 | XRCC2 | protein_coding |
| ENSG00000214021 | 623.890747 | 281.999025 | 1.14420338 | 4.22E-18 | 1.12E-16 | TTLL3 | protein_coding |
| ENSG00000102384 | 200.30971 | 488.675269 | -1.2840609 | 4.63E-18 | 1.22E-16 | CENPI | protein_coding |
| ENSG00000247626 | 229.266386 | 492.566893 | -1.1030347 | 5.46E-18 | 1.44E-16 | MARS2 | protein_coding |
| ENSG00000178878 | 457.333118 | 201.49006 | 1.18395587 | 7.47E-18 | 1.95E-16 | APOLD1 | protein_coding |
| ENSG00000178695 | 188.465578 | 452.69734 | -1.2646544 | 8.24E-18 | 2.14E-16 | KCTD12 | protein_coding |
| ENSG00000152253 | 203.632158 | 465.577962 | -1.1918428 | 8.41E-18 | 2.18E-16 | SPC25 | protein_coding |
| ENSG00000116661 | 643.229267 | 270.613674 | 1.25074286 | 1.69E-17 | 4.28E-16 | FBXO2 | protein_coding |
| ENSG00000162073 | 218.690077 | 479.559974 | -1.1320756 | 2.24E-17 | 5.61E-16 | PAQR4 | protein_coding |
| ENSG00000203760 | 193.199784 | 447.976612 | -1.2114751 | 3.31E-17 | 8.21E-16 | CENPW | protein_coding |
| ENSG00000143127 | 218.083876 | 491.483684 | -1.1715134 | 4.89E-17 | 1.20E-15 | ITGA10 | protein_coding |
| ENSG00000158402 | 236.996429 | 498.677157 | -1.072607 | 4.92E-17 | 1.20E-15 | CDC25C | protein_coding |
| ENSG00000165480 | 281.167125 | 568.160346 | -1.0151945 | 5.62E-17 | 1.37E-15 | SKA3 | protein_coding |
| ENSG00000188313 | 287.902629 | 576.961671 | -1.0026003 | 7.34E-17 | 1.77E-15 | PLSCR1 | protein_coding |
| ENSG00000186806 | 313.623237 | 116.080468 | 1.43621553 | 1.05E-16 | 2.52E-15 | VSIG10L | protein_coding |
| ENSG00000140939 | 528.970491 | 242.588317 | 1.12420728 | 1.07E-16 | 2.56E-15 | NOL3 | protein_coding |
| ENSG00000167740 | 271.553409 | 79.7018231 | 1.76981889 | 1.30E-16 | 3.09E-15 | CYB5D2 | protein_coding |
| ENSG00000260001 | 279.566273 | 86.7076463 | 1.68960359 | 1.32E-16 | 3.15E-15 | TGFBR3L | protein_coding |
| ENSG00000128578 | 165.881423 | 388.170904 | -1.2261006 | 1.43E-16 | 3.40E-15 | STRIP2 | protein_coding |
| ENSG00000162572 | 303.883774 | 106.364986 | 1.51338669 | 1.88E-16 | 4.41E-15 | SCNN1D | protein_coding |
| ENSG00000172232 | 129.605991 | 23.3817495 | 2.4680105 | 2.24E-16 | 5.24E-15 | AZU1 | protein_coding |
| ENSG00000162576 | 555.61539 | 273.092726 | 1.02401736 | 2.59E-16 | 5.99E-15 | MXRA8 | protein_coding |
| ENSG00000123689 | 94.0885473 | 275.036097 | -1.5455859 | 2.79E-16 | 6.44E-15 | G0S2 | protein_coding |
| ENSG00000181027 | 572.099044 | 276.485973 | 1.04790526 | 3.41E-16 | 7.83E-15 | FKRP | protein_coding |
| ENSG00000115257 | 366.324468 | 148.745056 | 1.29989594 | 3.63E-16 | 8.33E-15 | PCSK4 | protein_coding |
| ENSG00000144642 | 370.954635 | 155.37498 | 1.25612748 | 4.15E-16 | 9.48E-15 | RBMS3 | protein_coding |
| ENSG00000164463 | 446.171807 | 197.881846 | 1.17136698 | 4.35E-16 | 9.89E-15 | CREBRF | protein_coding |
| ENSG00000235109 | 355.972697 | 131.063863 | 1.44417154 | 4.46E-16 | 1.01E-14 | ZSCAN31 | protein_coding |
| ENSG00000242779 | 372.200593 | 127.061583 | 1.55327057 | 5.56E-16 | 1.25E-14 | ZNF702P | transcribed_processed_pseudogene |
| ENSG00000160703 | 479.178647 | 226.713763 | 1.07967703 | 5.96E-16 | 1.34E-14 | NLRX1 | protein_coding |
| ENSG00000133460 | 266.863497 | 92.4757545 | 1.52719051 | 6.83E-16 | 1.52E-14 | SLC2A11 | protein_coding |
| ENSG00000186481 | 259.324142 | 556.28628 | -1.1012744 | 1.01E-15 | 2.22E-14 | ANKRD20A5P | transcribed_unprocessed_pseudogene |
| ENSG00000271270 | 388.473771 | 155.94291 | 1.31799286 | 1.05E-15 | 2.29E-14 | TMCC1-AS1 | antisense |
| ENSG00000224411 | 138.652162 | 341.225519 | -1.297804 | 1.42E-15 | 3.08E-14 | HSP90AA2P | processed_pseudogene |
| ENSG00000041880 | 537.480891 | 267.439199 | 1.00754905 | 1.59E-15 | 3.41E-14 | PARP3 | protein_coding |
| ENSG00000172638 | 388.748693 | 163.711342 | 1.24733272 | 1.65E-15 | 3.55E-14 | EFEMP2 | protein_coding |
| ENSG00000234840 | 152.553466 | 31.3577511 | 2.27628093 | 1.76E-15 | 3.78E-14 | LINC01239 | lincRNA |
| ENSG00000233426 | 377.67217 | 164.039611 | 1.20480063 | 1.85E-15 | 3.96E-14 | EIF3FP3 | processed_pseudogene |
| ENSG00000086289 | 407.327949 | 176.844393 | 1.20446034 | 2.64E-15 | 5.61E-14 | EPDR1 | protein_coding |
| ENSG00000197020 | 360.737438 | 736.706921 | -1.0289431 | 2.66E-15 | 5.64E-14 | ZNF100 | protein_coding |
| ENSG00000138613 | 330.100656 | 126.530208 | 1.38268317 | 3.05E-15 | 6.44E-14 | APH1B | protein_coding |
| ENSG00000113578 | 91.4069039 | 256.36861 | -1.4859639 | 3.06E-15 | 6.45E-14 | FGF1 | protein_coding |
| ENSG00000267324 | 129.009108 | 327.742379 | -1.3458646 | 3.51E-15 | 7.35E-14 | AC006557.2 | processed_pseudogene |
| ENSG00000139178 | 204.054201 | 59.2840467 | 1.78206031 | 4.89E-15 | 1.01E-13 | C1RL | protein_coding |
| ENSG00000092758 | 151.925425 | 36.0761229 | 2.07398749 | 5.86E-15 | 1.20E-13 | COL9A3 | protein_coding |
| ENSG00000181513 | 330.455239 | 121.079211 | 1.44712214 | 9.02E-15 | 1.82E-13 | ACBD4 | protein_coding |
| ENSG00000173531 | 291.722746 | 95.2094058 | 1.61047045 | 9.42E-15 | 1.90E-13 | MST1 | protein_coding |
| ENSG00000144455 | 404.684591 | 172.057601 | 1.23459332 | 9.51E-15 | 1.92E-13 | SUMF1 | protein_coding |
| ENSG00000144597 | 182.87085 | 416.966183 | -1.188058 | 9.57E-15 | 1.93E-13 | EAF1 | protein_coding |
| ENSG00000143303 | 375.127672 | 166.515167 | 1.17245874 | 1.04E-14 | 2.09E-13 | RRNAD1 | protein_coding |
| ENSG00000174939 | 839.353789 | 415.366905 | 1.01661429 | 1.10E-14 | 2.21E-13 | ASPHD1 | protein_coding |
| ENSG00000133302 | 185.914237 | 401.38533 | -1.109358 | 1.11E-14 | 2.22E-13 | SLF1 | protein_coding |
| ENSG00000121481 | 237.403759 | 489.213033 | -1.0432659 | 1.21E-14 | 2.42E-13 | RNF2 | protein_coding |
| ENSG00000105479 | 355.729333 | 147.949552 | 1.26468576 | 1.22E-14 | 2.43E-13 | CCDC114 | protein_coding |
| ENSG00000151849 | 219.095389 | 451.610743 | -1.0443412 | 1.58E-14 | 3.12E-13 | CENPJ | protein_coding |
| ENSG00000075399 | 591.934105 | 278.179639 | 1.09003007 | 1.71E-14 | 3.36E-13 | VPS9D1 | protein_coding |
| ENSG00000198520 | 162.81998 | 41.7191509 | 1.9621703 | 1.76E-14 | 3.45E-13 | ARMH1 | protein_coding |
| ENSG00000229953 | 253.845265 | 87.2331952 | 1.5444118 | 1.78E-14 | 3.49E-13 | AL590666.2 | antisense |
| ENSG00000159055 | 224.898505 | 468.929695 | -1.0607494 | 2.14E-14 | 4.17E-13 | MIS18A | protein_coding |
| ENSG00000030110 | 374.932403 | 758.084327 | -1.0153845 | 4.61E-14 | 8.77E-13 | BAK1 | protein_coding |
| ENSG00000010626 | 366.761578 | 165.718485 | 1.14535047 | 4.70E-14 | 8.92E-13 | LRRC23 | protein_coding |
| ENSG00000160323 | 217.501334 | 57.3929543 | 1.91608199 | 4.82E-14 | 9.14E-13 | ADAMTS13 | protein_coding |
| ENSG00000113594 | 177.753258 | 381.867534 | -1.1028553 | 5.44E-14 | 1.03E-12 | LIFR | protein_coding |
| ENSG00000182796 | 503.080964 | 247.227631 | 1.02283058 | 6.81E-14 | 1.27E-12 | TMEM198B | transcribed_unitary_pseudogene |
| ENSG00000100889 | 337.151117 | 127.318564 | 1.40283386 | 7.98E-14 | 1.48E-12 | PCK2 | protein_coding |
| ENSG00000198890 | 415.941757 | 179.126841 | 1.21714663 | 8.21E-14 | 1.52E-12 | PRMT6 | protein_coding |
| ENSG00000234880 | 113.754595 | 20.1705729 | 2.49894809 | 9.87E-14 | 1.82E-12 | LINC00163 | lincRNA |
| ENSG00000261373 | 352.746772 | 149.324809 | 1.23793177 | 9.92E-14 | 1.83E-12 | VPS9D1-AS1 | antisense |
| ENSG00000114735 | 455.280926 | 226.592362 | 1.00765515 | 1.20E-13 | 2.19E-12 | HEMK1 | protein_coding |
| ENSG00000008517 | 72.0912304 | 219.721909 | -1.6046434 | 1.26E-13 | 2.30E-12 | IL32 | protein_coding |
| ENSG00000174292 | 258.846738 | 86.0674334 | 1.58586384 | 1.27E-13 | 2.31E-12 | TNK1 | protein_coding |
| ENSG00000285108 | 75.9987672 | 212.71059 | -1.4844285 | 1.65E-13 | 2.99E-12 | AC103718.1 | lincRNA |
| ENSG00000163703 | 338.143395 | 148.470783 | 1.18767273 | 1.72E-13 | 3.10E-12 | CRELD1 | protein_coding |
| ENSG00000261706 | 151.383834 | 38.0403461 | 1.99683415 | 1.77E-13 | 3.19E-12 | LINC00165 | lincRNA |
| ENSG00000168874 | 303.178713 | 114.234049 | 1.41181872 | 3.10E-13 | 5.50E-12 | ATOH8 | protein_coding |
| ENSG00000273151 | 302.320626 | 128.694853 | 1.22975324 | 4.80E-13 | 8.39E-12 | AC073957.3 | antisense |
| ENSG00000152056 | 158.020565 | 338.395162 | -1.0981933 | 4.93E-13 | 8.60E-12 | AP1S3 | protein_coding |
| ENSG00000180626 | 443.958977 | 215.610069 | 1.04283718 | 5.95E-13 | 1.03E-11 | ZNF594 | protein_coding |
| ENSG00000000460 | 162.946585 | 347.869329 | -1.0938561 | 5.96E-13 | 1.03E-11 | C1orf112 | protein_coding |
| ENSG00000129173 | 193.393546 | 392.199653 | -1.0189107 | 7.45E-13 | 1.28E-11 | E2F8 | protein_coding |
| ENSG00000075240 | 429.198401 | 206.312413 | 1.05860639 | 7.84E-13 | 1.35E-11 | GRAMD4 | protein_coding |
| ENSG00000230733 | 245.923296 | 499.564282 | -1.0225067 | 9.75E-13 | 1.66E-11 | AC092171.2 | lincRNA |
| ENSG00000163794 | 233.226809 | 79.1608172 | 1.5590528 | 1.05E-12 | 1.78E-11 | UCN | protein_coding |
| ENSG00000153531 | 149.90563 | 41.7913509 | 1.84158443 | 1.16E-12 | 1.95E-11 | ADPRHL1 | protein_coding |
| ENSG00000132436 | 141.074706 | 319.839837 | -1.1826156 | 1.20E-12 | 2.02E-11 | FIGNL1 | protein_coding |
| ENSG00000135637 | 371.504038 | 179.258495 | 1.05007895 | 1.40E-12 | 2.34E-11 | CCDC142 | protein_coding |
| ENSG00000196338 | 173.501323 | 46.670656 | 1.88773205 | 1.40E-12 | 2.34E-11 | NLGN3 | protein_coding |
| ENSG00000138380 | 150.810558 | 42.2462703 | 1.83738061 | 1.48E-12 | 2.46E-11 | CARF | protein_coding |
| ENSG00000099219 | 228.033097 | 458.423706 | -1.0073704 | 1.61E-12 | 2.66E-11 | ERMP1 | protein_coding |
| ENSG00000168389 | 34.8133352 | 137.410192 | -1.9825402 | 1.63E-12 | 2.69E-11 | MFSD2A | protein_coding |
| ENSG00000169297 | 376.999905 | 154.290176 | 1.29136209 | 1.68E-12 | 2.77E-11 | NR0B1 | protein_coding |
| ENSG00000129810 | 109.810436 | 276.8005 | -1.3339848 | 1.73E-12 | 2.85E-11 | SGO1 | protein_coding |
| ENSG00000145217 | 257.343776 | 99.1876297 | 1.37782173 | 1.76E-12 | 2.89E-11 | SLC26A1 | protein_coding |
| ENSG00000181026 | 285.845399 | 577.859295 | -1.0161741 | 1.77E-12 | 2.92E-11 | AEN | protein_coding |
| ENSG00000107443 | 209.522608 | 427.152934 | -1.0264763 | 2.29E-12 | 3.73E-11 | CCNJ | protein_coding |
| ENSG00000091947 | 637.159403 | 316.721687 | 1.00983505 | 2.47E-12 | 4.02E-11 | TMEM101 | protein_coding |
| ENSG00000117899 | 416.16284 | 207.828144 | 1.00375652 | 2.85E-12 | 4.61E-11 | MESD | protein_coding |
| ENSG00000248810 | 295.734718 | 128.528816 | 1.20419702 | 3.79E-12 | 6.05E-11 | LINC02432 | lincRNA |
| ENSG00000109339 | 136.914346 | 35.5862814 | 1.94246637 | 3.85E-12 | 6.14E-11 | MAPK10 | protein_coding |
| ENSG00000185761 | 555.003613 | 275.810483 | 1.0086033 | 4.07E-12 | 6.48E-11 | ADAMTSL5 | protein_coding |
| ENSG00000165501 | 107.221747 | 269.094619 | -1.3301159 | 4.60E-12 | 7.27E-11 | LRR1 | protein_coding |
| ENSG00000118242 | 189.208698 | 392.797402 | -1.051926 | 4.78E-12 | 7.54E-11 | MREG | protein_coding |
| ENSG00000140057 | 190.554139 | 64.7388241 | 1.55852979 | 5.42E-12 | 8.50E-11 | AK7 | protein_coding |
| ENSG00000204611 | 264.575553 | 106.724314 | 1.30893714 | 6.01E-12 | 9.38E-11 | ZNF616 | protein_coding |
| ENSG00000100647 | 200.512877 | 408.159692 | -1.0246583 | 6.76E-12 | 1.05E-10 | SUSD6 | protein_coding |
| ENSG00000233559 | 183.875062 | 64.0810457 | 1.51898838 | 7.27E-12 | 1.13E-10 | LINC00513 | lincRNA |
| ENSG00000273270 | 141.08643 | 32.9782487 | 2.09165805 | 7.38E-12 | 1.15E-10 | AC090114.2 | lincRNA |
| ENSG00000167103 | 223.653162 | 88.9672421 | 1.33141181 | 1.05E-11 | 1.61E-10 | PIP5KL1 | protein_coding |
| ENSG00000176208 | 186.487159 | 380.400573 | -1.0301513 | 1.10E-11 | 1.68E-10 | ATAD5 | protein_coding |
| ENSG00000144488 | 165.036455 | 53.2126314 | 1.63442421 | 1.20E-11 | 1.83E-10 | ESPNL | protein_coding |
| ENSG00000188191 | 306.902161 | 141.452789 | 1.1157998 | 1.40E-11 | 2.13E-10 | PRKAR1B | protein_coding |
| ENSG00000106976 | 245.310392 | 100.333103 | 1.28766973 | 1.53E-11 | 2.32E-10 | DNM1 | protein_coding |
| ENSG00000177602 | 158.019769 | 326.370345 | -1.0467675 | 1.55E-11 | 2.34E-10 | HASPIN | protein_coding |
| ENSG00000100784 | 116.310801 | 271.523393 | -1.2218156 | 2.22E-11 | 3.31E-10 | RPS6KA5 | protein_coding |
| ENSG00000161558 | 257.491139 | 103.694113 | 1.31428365 | 2.30E-11 | 3.43E-10 | TMEM143 | protein_coding |
| ENSG00000230487 | 202.141788 | 73.1315365 | 1.46878967 | 2.98E-11 | 4.39E-10 | PSMG3-AS1 | lincRNA |
| ENSG00000078487 | 125.480219 | 33.1885671 | 1.91722073 | 3.16E-11 | 4.64E-10 | ZCWPW1 | protein_coding |
| ENSG00000185522 | 250.646644 | 95.5275682 | 1.39218061 | 3.40E-11 | 4.98E-10 | LMNTD2 | protein_coding |
| ENSG00000229043 | 197.56738 | 70.6259148 | 1.48534637 | 3.74E-11 | 5.47E-10 | AC091729.3 | antisense |
| ENSG00000109854 | 183.914846 | 66.7188968 | 1.46237877 | 5.48E-11 | 7.87E-10 | HTATIP2 | protein_coding |
| ENSG00000091986 | 196.751396 | 75.9199678 | 1.37508295 | 6.49E-11 | 9.29E-10 | CCDC80 | protein_coding |
| ENSG00000188177 | 322.830247 | 143.527915 | 1.17073057 | 1.06E-10 | 1.49E-09 | ZC3H6 | protein_coding |
| ENSG00000169962 | 112.01335 | 29.0548429 | 1.95058808 | 1.10E-10 | 1.53E-09 | TAS1R3 | protein_coding |
| ENSG00000248508 | 176.131916 | 60.4170582 | 1.54024696 | 1.90E-10 | 2.58E-09 | SRP14-AS1 | lincRNA |
| ENSG00000165698 | 211.428562 | 88.7965571 | 1.25176542 | 1.94E-10 | 2.63E-09 | SPACA9 | protein_coding |
| ENSG00000148942 | 262.408638 | 114.54738 | 1.19565363 | 1.96E-10 | 2.66E-09 | SLC5A12 | protein_coding |
| ENSG00000100979 | 222.145904 | 95.3835609 | 1.2203675 | 2.06E-10 | 2.79E-09 | PLTP | protein_coding |
| ENSG00000162004 | 231.101225 | 95.2673266 | 1.27587897 | 2.30E-10 | 3.12E-09 | CCDC78 | protein_coding |
| ENSG00000161980 | 165.962937 | 336.954708 | -1.0210708 | 2.57E-10 | 3.45E-09 | POLR3K | protein_coding |
| ENSG00000161920 | 172.279387 | 63.1505005 | 1.44936153 | 3.00E-10 | 4.00E-09 | MED11 | protein_coding |
| ENSG00000082497 | 48.5111166 | 145.079891 | -1.5815739 | 3.46E-10 | 4.57E-09 | SERTAD4 | protein_coding |
| ENSG00000074621 | 259.172354 | 115.034227 | 1.17028017 | 3.58E-10 | 4.72E-09 | SLC24A1 | protein_coding |
| ENSG00000204366 | 217.867106 | 88.3673846 | 1.30231743 | 3.67E-10 | 4.84E-09 | ZBTB12 | protein_coding |
| ENSG00000132692 | 210.563261 | 82.7992034 | 1.34541736 | 4.28E-10 | 5.60E-09 | BCAN | protein_coding |
| ENSG00000205670 | 130.800718 | 22.8509512 | 2.5189142 | 4.48E-10 | 5.85E-09 | SMIM11A | protein_coding |
| ENSG00000189143 | 200.515068 | 80.2877942 | 1.32193102 | 4.64E-10 | 6.04E-09 | CLDN4 | protein_coding |
| ENSG00000257261 | 74.0161228 | 13.3287606 | 2.47726493 | 4.97E-10 | 6.46E-09 | AC008014.1 | lincRNA |
| ENSG00000178082 | 107.889415 | 244.349226 | -1.1786297 | 5.62E-10 | 7.26E-09 | TWF1P1 | processed_pseudogene |
| ENSG00000167173 | 137.92876 | 310.270839 | -1.1699405 | 6.43E-10 | 8.25E-09 | C15orf39 | protein_coding |
| ENSG00000187790 | 148.781428 | 305.484256 | -1.0361899 | 7.37E-10 | 9.38E-09 | FANCM | protein_coding |
| ENSG00000172086 | 217.63699 | 93.5804548 | 1.21919124 | 7.78E-10 | 9.85E-09 | KRCC1 | protein_coding |
| ENSG00000114841 | 454.969791 | 218.937505 | 1.05228741 | 8.15E-10 | 1.03E-08 | DNAH1 | protein_coding |
| ENSG00000144485 | 188.430375 | 377.678035 | -1.0011123 | 9.10E-10 | 1.15E-08 | HES6 | protein_coding |
| ENSG00000147576 | 138.942593 | 40.4548407 | 1.77606053 | 9.13E-10 | 1.15E-08 | ADHFE1 | protein_coding |
| ENSG00000112812 | 250.919391 | 112.85954 | 1.15047947 | 9.34E-10 | 1.17E-08 | PRSS16 | protein_coding |
| ENSG00000118898 | 132.617762 | 42.8119908 | 1.6298399 | 1.01E-09 | 1.26E-08 | PPL | protein_coding |
| ENSG00000106479 | 300.302936 | 145.55066 | 1.04332144 | 1.02E-09 | 1.27E-08 | ZNF862 | protein_coding |
| ENSG00000067992 | 131.651496 | 285.345712 | -1.1145717 | 1.03E-09 | 1.28E-08 | PDK3 | protein_coding |
| ENSG00000198003 | 136.084871 | 45.7424665 | 1.57127018 | 1.07E-09 | 1.34E-08 | CCDC151 | protein_coding |
| ENSG00000198948 | 164.652628 | 336.09219 | -1.0272019 | 1.11E-09 | 1.39E-08 | MFAP3L | protein_coding |
| ENSG00000125898 | 147.688557 | 300.02359 | -1.0206959 | 1.13E-09 | 1.41E-08 | FAM110A | protein_coding |
| ENSG00000177181 | 24.6721881 | 97.319536 | -1.9780961 | 1.19E-09 | 1.47E-08 | RIMKLA | protein_coding |
| ENSG00000220785 | 290.860433 | 133.326809 | 1.12172016 | 1.46E-09 | 1.79E-08 | MTMR9LP | transcribed_unprocessed_pseudogene |
| ENSG00000154134 | 260.73041 | 124.382446 | 1.06689018 | 1.52E-09 | 1.86E-08 | ROBO3 | protein_coding |
| ENSG00000169252 | 118.557725 | 257.360793 | -1.1164918 | 1.56E-09 | 1.91E-08 | ADRB2 | protein_coding |
| ENSG00000171346 | 81.6858511 | 195.435425 | -1.2594523 | 1.62E-09 | 1.98E-08 | KRT15 | protein_coding |
| ENSG00000172728 | 127.68083 | 270.463695 | -1.0832578 | 1.80E-09 | 2.20E-08 | FUT10 | protein_coding |
| ENSG00000123612 | 40.2281299 | 125.760305 | -1.6463232 | 1.83E-09 | 2.23E-08 | ACVR1C | protein_coding |
| ENSG00000101945 | 142.316975 | 287.31533 | -1.0145903 | 2.30E-09 | 2.79E-08 | SUV39H1 | protein_coding |
| ENSG00000163704 | 178.804225 | 69.4414096 | 1.36280065 | 2.38E-09 | 2.88E-08 | PRRT3 | protein_coding |
| ENSG00000068079 | 282.365827 | 136.739705 | 1.04859314 | 2.70E-09 | 3.25E-08 | IFI35 | protein_coding |
| ENSG00000265415 | 155.184146 | 312.509754 | -1.0113499 | 2.71E-09 | 3.26E-08 | AC099850.3 | antisense |
| ENSG00000226435 | 71.0804022 | 175.913664 | -1.3095097 | 2.98E-09 | 3.56E-08 | ANKRD18DP | transcribed_unprocessed_pseudogene |
| ENSG00000258376 | 194.414307 | 83.932462 | 1.21096103 | 2.99E-09 | 3.57E-08 | AC004846.1 | antisense |
| ENSG00000258701 | 170.261713 | 65.7212554 | 1.37670063 | 3.22E-09 | 3.83E-08 | LINC00638 | lincRNA |
| ENSG00000140905 | 80.795636 | 194.485313 | -1.2694093 | 3.24E-09 | 3.85E-08 | GCSH | protein_coding |
| ENSG00000101213 | 101.91991 | 27.360575 | 1.90081622 | 3.33E-09 | 3.95E-08 | PTK6 | protein_coding |
| ENSG00000213707 | 14.3846307 | 90.0336957 | -2.6569408 | 3.34E-09 | 3.96E-08 | HMGB1P10 | processed_pseudogene |
| ENSG00000222009 | 307.939756 | 150.779725 | 1.02761329 | 3.48E-09 | 4.12E-08 | BTBD19 | protein_coding |
| ENSG00000138756 | 135.375827 | 273.452238 | -1.0146907 | 3.52E-09 | 4.16E-08 | BMP2K | protein_coding |
| ENSG00000180998 | 61.6796304 | 160.468922 | -1.3807666 | 3.56E-09 | 4.20E-08 | GPR137C | protein_coding |
| ENSG00000213983 | 86.4896905 | 17.110369 | 2.33085917 | 3.60E-09 | 4.24E-08 | AP1G2 | protein_coding |
| ENSG00000198780 | 132.635904 | 265.352789 | -1.0005055 | 3.63E-09 | 4.28E-08 | FAM169A | protein_coding |
| ENSG00000181544 | 94.5494198 | 224.180286 | -1.2422752 | 3.65E-09 | 4.30E-08 | FANCB | protein_coding |
| ENSG00000135899 | 122.097897 | 256.252375 | -1.0687898 | 3.81E-09 | 4.48E-08 | SP110 | protein_coding |
| ENSG00000260285 | 154.548718 | 55.1233979 | 1.48511811 | 4.78E-09 | 5.53E-08 | AL133367.1 | antisense |
| ENSG00000196388 | 173.055675 | 64.0096309 | 1.43195939 | 4.96E-09 | 5.74E-08 | INCA1 | protein_coding |
| ENSG00000100802 | 241.734221 | 117.213087 | 1.04501764 | 4.97E-09 | 5.74E-08 | C14orf93 | protein_coding |
| ENSG00000073150 | 151.96364 | 51.4385585 | 1.56730347 | 5.07E-09 | 5.85E-08 | PANX2 | protein_coding |
| ENSG00000152661 | 41.2352603 | 127.582484 | -1.6274336 | 5.44E-09 | 6.25E-08 | GJA1 | protein_coding |
| ENSG00000205464 | 145.726687 | 49.1079404 | 1.5743857 | 5.53E-09 | 6.35E-08 | ATP6AP1L | protein_coding |
| ENSG00000101265 | 42.0292482 | 123.464217 | -1.5546579 | 6.82E-09 | 7.74E-08 | RASSF2 | protein_coding |
| ENSG00000280187 | 95.9475908 | 24.7207609 | 1.95393824 | 6.94E-09 | 7.86E-08 | AC022107.1 | TEC |
| ENSG00000226415 | 49.4959657 | 141.688854 | -1.52119 | 7.02E-09 | 7.94E-08 | TPI1P1 | processed_pseudogene |
| ENSG00000024862 | 116.828076 | 259.890014 | -1.1565628 | 7.94E-09 | 8.93E-08 | CCDC28A | protein_coding |
| ENSG00000204583 | 98.0982004 | 21.2721956 | 2.20090277 | 8.30E-09 | 9.31E-08 | LRCOL1 | protein_coding |
| ENSG00000179523 | 227.672946 | 106.618763 | 1.09237456 | 9.10E-09 | 1.02E-07 | EIF3J-DT | lincRNA |
| ENSG00000204934 | 140.707869 | 50.7005825 | 1.47611999 | 9.26E-09 | 1.03E-07 | ATP6V0E2-AS1 | antisense |
| ENSG00000235706 | 217.212228 | 94.8108368 | 1.19277063 | 9.45E-09 | 1.05E-07 | DICER1-AS1 | antisense |
| ENSG00000280206 | 109.571868 | 236.703519 | -1.1083424 | 1.09E-08 | 1.21E-07 | AC026401.3 | lincRNA |
| ENSG00000165171 | 211.139881 | 91.1316394 | 1.20969984 | 1.13E-08 | 1.25E-07 | METTL27 | protein_coding |
| ENSG00000197275 | 57.979772 | 154.760451 | -1.4125501 | 1.30E-08 | 1.41E-07 | RAD54B | protein_coding |
| ENSG00000197568 | 121.640809 | 40.6659443 | 1.57865929 | 1.43E-08 | 1.55E-07 | HHLA3 | protein_coding |
| ENSG00000260428 | 105.409544 | 32.1909257 | 1.71489262 | 1.45E-08 | 1.56E-07 | SCX | protein_coding |
| ENSG00000179715 | 145.190189 | 55.4015977 | 1.38928462 | 1.48E-08 | 1.60E-07 | PCED1B | protein_coding |
| ENSG00000089847 | 95.9057729 | 24.4814093 | 1.96190599 | 1.50E-08 | 1.62E-07 | ANKRD24 | protein_coding |
| ENSG00000088367 | 291.055277 | 143.482209 | 1.01793601 | 1.58E-08 | 1.70E-07 | EPB41L1 | protein_coding |
| ENSG00000186301 | 158.98296 | 59.9184339 | 1.40530164 | 1.64E-08 | 1.76E-07 | MST1P2 | unprocessed_pseudogene |
| ENSG00000108187 | 145.601675 | 54.3035083 | 1.42564746 | 1.73E-08 | 1.85E-07 | PBLD | protein_coding |
| ENSG00000198331 | 145.849847 | 295.687874 | -1.0176503 | 1.83E-08 | 1.95E-07 | HYLS1 | protein_coding |
| ENSG00000166046 | 141.357126 | 53.32304 | 1.40489418 | 1.92E-08 | 2.05E-07 | TCP11L2 | protein_coding |
| ENSG00000135074 | 46.0000012 | 131.547486 | -1.5144013 | 2.09E-08 | 2.22E-07 | ADAM19 | protein_coding |
| ENSG00000173926 | 118.95151 | 254.287633 | -1.0940356 | 2.10E-08 | 2.23E-07 | 3-Mar | protein_coding |
| ENSG00000203791 | 113.088962 | 232.583643 | -1.0417793 | 2.40E-08 | 2.53E-07 | EEF1AKMT2 | protein_coding |
| ENSG00000179841 | 107.378116 | 230.454168 | -1.0990576 | 2.49E-08 | 2.63E-07 | AKAP5 | protein_coding |
| ENSG00000166831 | 117.47806 | 247.614131 | -1.0764875 | 2.63E-08 | 2.77E-07 | RBPMS2 | protein_coding |
| ENSG00000175305 | 124.058346 | 253.450843 | -1.0327533 | 2.81E-08 | 2.95E-07 | CCNE2 | protein_coding |
| ENSG00000007255 | 112.519981 | 36.2563759 | 1.63068019 | 2.97E-08 | 3.10E-07 | TRAPPC6A | protein_coding |
| ENSG00000154146 | 92.8421388 | 204.401399 | -1.1379239 | 3.00E-08 | 3.13E-07 | NRGN | protein_coding |
| ENSG00000103150 | 273.303436 | 134.042362 | 1.02668158 | 3.30E-08 | 3.42E-07 | MLYCD | protein_coding |
| ENSG00000266714 | 135.732376 | 35.9869589 | 1.90702684 | 3.44E-08 | 3.56E-07 | MYO15B | protein_coding |
| ENSG00000187634 | 188.615331 | 78.6204509 | 1.26659239 | 3.81E-08 | 3.93E-07 | SAMD11 | protein_coding |
| ENSG00000276805 | 223.269776 | 106.07447 | 1.07462748 | 4.39E-08 | 4.46E-07 | AL133216.2 | transcribed_unprocessed_pseudogene |
| ENSG00000025434 | 165.78097 | 70.207488 | 1.24072842 | 4.49E-08 | 4.55E-07 | NR1H3 | protein_coding |
| ENSG00000151470 | 14.0076625 | 69.5545664 | -2.3231189 | 4.53E-08 | 4.59E-07 | C4orf33 | protein_coding |
| ENSG00000184489 | 147.427439 | 53.2689818 | 1.47324796 | 4.67E-08 | 4.73E-07 | PTP4A3 | protein_coding |
| ENSG00000099338 | 163.202326 | 60.5627816 | 1.42561988 | 5.66E-08 | 5.67E-07 | CATSPERG | protein_coding |
| ENSG00000008513 | 76.5322608 | 187.230216 | -1.2886724 | 5.83E-08 | 5.83E-07 | ST3GAL1 | protein_coding |
| ENSG00000160766 | 167.339381 | 71.9407497 | 1.21749006 | 5.95E-08 | 5.93E-07 | GBAP1 | transcribed_unprocessed_pseudogene |
| ENSG00000285887 | 199.874634 | 88.5207129 | 1.17456498 | 6.20E-08 | 6.17E-07 | AL009176.1 | lincRNA |
| ENSG00000146021 | 269.203817 | 128.845433 | 1.05946926 | 6.68E-08 | 6.63E-07 | KLHL3 | protein_coding |
| ENSG00000253540 | 147.414675 | 51.9651016 | 1.49807132 | 6.74E-08 | 6.68E-07 | FAM86HP | transcribed_unprocessed_pseudogene |
| ENSG00000102878 | 243.058203 | 121.246363 | 1.00386956 | 7.45E-08 | 7.35E-07 | HSF4 | protein_coding |
| ENSG00000110013 | 197.579789 | 91.1930936 | 1.11451879 | 7.71E-08 | 7.58E-07 | SIAE | protein_coding |
| ENSG00000175197 | 244.019888 | 119.172991 | 1.03572577 | 7.86E-08 | 7.72E-07 | DDIT3 | protein_coding |
| ENSG00000183155 | 90.3334831 | 195.510518 | -1.1133763 | 9.01E-08 | 8.77E-07 | RABIF | protein_coding |
| ENSG00000114698 | 43.4461847 | 126.080785 | -1.5323134 | 1.03E-07 | 1.00E-06 | PLSCR4 | protein_coding |
| ENSG00000198939 | 70.9267781 | 16.9297234 | 2.06280981 | 1.03E-07 | 1.00E-06 | ZFP2 | protein_coding |
| ENSG00000184545 | 129.198731 | 48.5521804 | 1.41370227 | 1.04E-07 | 1.01E-06 | DUSP8 | protein_coding |
| ENSG00000168071 | 213.492123 | 102.429803 | 1.06221477 | 1.20E-07 | 1.15E-06 | CCDC88B | protein_coding |
| ENSG00000133466 | 216.46676 | 97.8396519 | 1.14485969 | 1.33E-07 | 1.28E-06 | C1QTNF6 | protein_coding |
| ENSG00000012171 | 125.836655 | 39.16189 | 1.67591804 | 1.43E-07 | 1.36E-06 | SEMA3B | protein_coding |
| ENSG00000283050 | 142.69131 | 56.7533178 | 1.32694008 | 1.52E-07 | 1.44E-06 | GTF2IP12 | transcribed_unprocessed_pseudogene |
| ENSG00000132481 | 105.266406 | 217.20893 | -1.0428517 | 1.59E-07 | 1.50E-06 | TRIM47 | protein_coding |
| ENSG00000077935 | 119.145272 | 43.9742826 | 1.43961264 | 1.73E-07 | 1.63E-06 | SMC1B | protein_coding |
| ENSG00000106003 | 20.8592853 | 76.8469795 | -1.8826531 | 1.77E-07 | 1.66E-06 | LFNG | protein_coding |
| ENSG00000140398 | 202.327115 | 82.4816805 | 1.28949928 | 2.05E-07 | 1.91E-06 | NEIL1 | protein_coding |
| ENSG00000269896 | 68.7338225 | 16.7471148 | 2.04105888 | 2.15E-07 | 1.99E-06 | AL513477.1 | transcribed_processed_pseudogene |
| ENSG00000168916 | 69.4130164 | 14.6432029 | 2.23231521 | 2.19E-07 | 2.03E-06 | ZNF608 | protein_coding |
| ENSG00000205593 | 167.350024 | 70.3257485 | 1.2500158 | 2.21E-07 | 2.04E-06 | DENND6B | protein_coding |
| ENSG00000268655 | 108.973037 | 38.4365595 | 1.50230472 | 2.22E-07 | 2.04E-06 | AC008687.4 | protein_coding |
| ENSG00000158246 | 72.1145514 | 161.297778 | -1.1629584 | 2.24E-07 | 2.06E-06 | TENT5B | protein_coding |
| ENSG00000183346 | 51.040019 | 7.65645244 | 2.71984296 | 2.34E-07 | 2.14E-06 | CABCOCO1 | protein_coding |
| ENSG00000174021 | 109.594392 | 226.99872 | -1.051193 | 2.37E-07 | 2.17E-06 | GNG5 | protein_coding |
| ENSG00000147124 | 172.566917 | 77.2656533 | 1.15926471 | 2.53E-07 | 2.32E-06 | ZNF41 | protein_coding |
| ENSG00000212724 | 55.4282333 | 134.22026 | -1.2784563 | 2.62E-07 | 2.39E-06 | KRTAP2-3 | protein_coding |
| ENSG00000272419 | 165.246168 | 72.9934181 | 1.18106944 | 3.17E-07 | 2.87E-06 | LINC01145 | transcribed_unprocessed_pseudogene |
| ENSG00000117122 | 214.918707 | 103.191626 | 1.05632299 | 3.24E-07 | 2.92E-06 | MFAP2 | protein_coding |
| ENSG00000049130 | 60.5986408 | 142.775805 | -1.2382055 | 3.31E-07 | 2.99E-06 | KITLG | protein_coding |
| ENSG00000163072 | 41.4348417 | 123.253113 | -1.5667377 | 3.38E-07 | 3.05E-06 | NOSTRIN | protein_coding |
| ENSG00000250986 | 63.3481678 | 15.3052999 | 2.04997641 | 3.39E-07 | 3.05E-06 | LINC02600 | lincRNA |
| ENSG00000143341 | 152.869253 | 65.6007026 | 1.21697542 | 3.40E-07 | 3.06E-06 | HMCN1 | protein_coding |
| ENSG00000153363 | 207.062274 | 102.213842 | 1.02098875 | 3.76E-07 | 3.36E-06 | LINC00467 | lincRNA |
| ENSG00000142233 | 109.096126 | 36.4385919 | 1.57929634 | 3.85E-07 | 3.44E-06 | NTN5 | protein_coding |
| ENSG00000105639 | 202.5436 | 91.2547568 | 1.14690383 | 3.97E-07 | 3.54E-06 | JAK3 | protein_coding |
| ENSG00000277462 | 96.568063 | 197.001927 | -1.0289745 | 4.00E-07 | 3.56E-06 | ZNF670 | protein_coding |
| ENSG00000225031 | 183.994524 | 88.7592794 | 1.05139817 | 4.38E-07 | 3.87E-06 | EIF4BP7 | processed_pseudogene |
| ENSG00000053501 | 244.476848 | 115.489146 | 1.08002027 | 4.56E-07 | 4.02E-06 | USE1 | protein_coding |
| ENSG00000226137 | 139.706999 | 59.8127364 | 1.22383124 | 5.65E-07 | 4.91E-06 | BAIAP2-DT | lincRNA |
| ENSG00000248323 | 202.232922 | 96.7289994 | 1.06375034 | 5.70E-07 | 4.95E-06 | LUCAT1 | antisense |
| ENSG00000198300 | 80.8927462 | 186.254212 | -1.2027327 | 5.84E-07 | 5.06E-06 | PEG3 | protein_coding |
| ENSG00000075213 | 176.134991 | 82.314073 | 1.09521005 | 6.35E-07 | 5.48E-06 | SEMA3A | protein_coding |
| ENSG00000101049 | 100.753708 | 33.6169544 | 1.57756744 | 6.78E-07 | 5.84E-06 | SGK2 | protein_coding |
| ENSG00000148225 | 176.801862 | 86.680329 | 1.02807886 | 7.61E-07 | 6.50E-06 | WDR31 | protein_coding |
| ENSG00000111254 | 68.0183613 | 18.4755198 | 1.8851523 | 8.62E-07 | 7.32E-06 | AKAP3 | protein_coding |
| ENSG00000266010 | 85.9060137 | 26.336009 | 1.70982728 | 8.91E-07 | 7.56E-06 | GATA6-AS1 | antisense |
| ENSG00000120899 | 91.141032 | 25.6516986 | 1.81875095 | 9.03E-07 | 7.64E-06 | PTK2B | protein_coding |
| ENSG00000238266 | 59.8004269 | 139.269465 | -1.2215827 | 9.43E-07 | 7.95E-06 | LINC00707 | lincRNA |
| ENSG00000156535 | 69.818683 | 17.7785006 | 1.96577557 | 1.02E-06 | 8.56E-06 | CD109 | protein_coding |
| ENSG00000179954 | 121.100015 | 47.6487688 | 1.34349674 | 1.08E-06 | 9.06E-06 | SSC5D | protein_coding |
| ENSG00000197479 | 170.398235 | 75.636126 | 1.17083775 | 1.09E-06 | 9.09E-06 | PCDHB11 | protein_coding |
| ENSG00000184524 | 55.9372543 | 12.6582734 | 2.14716627 | 1.21E-06 | 1.00E-05 | CEND1 | protein_coding |
| ENSG00000149927 | 92.5985482 | 32.4140985 | 1.51069565 | 1.23E-06 | 1.02E-05 | DOC2A | protein_coding |
| ENSG00000186994 | 125.655129 | 49.9054709 | 1.33038026 | 1.31E-06 | 1.08E-05 | KANK3 | protein_coding |
| ENSG00000172006 | 134.310858 | 56.1331465 | 1.26071137 | 1.39E-06 | 1.14E-05 | ZNF554 | protein_coding |
| ENSG00000157927 | 82.113799 | 170.050749 | -1.048954 | 1.42E-06 | 1.16E-05 | RADIL | protein_coding |
| ENSG00000269397 | 41.8579237 | 113.342707 | -1.4428813 | 1.53E-06 | 1.25E-05 | AC011503.2 | sense_intronic |
| ENSG00000136379 | 42.2157968 | 107.37856 | -1.3454935 | 1.55E-06 | 1.26E-05 | ABHD17C | protein_coding |
| ENSG00000213846 | 70.327349 | 20.8394897 | 1.75728505 | 1.64E-06 | 1.32E-05 | AC098614.1 | transcribed_processed_pseudogene |
| ENSG00000149591 | 138.722592 | 62.1033286 | 1.159372 | 1.64E-06 | 1.33E-05 | TAGLN | protein_coding |
| ENSG00000197355 | 189.023915 | 82.2733632 | 1.20198094 | 1.65E-06 | 1.33E-05 | UAP1L1 | protein_coding |
| ENSG00000172748 | 145.410632 | 63.6908669 | 1.18855243 | 1.66E-06 | 1.34E-05 | ZNF596 | protein_coding |
| ENSG00000085117 | 93.4312797 | 199.383583 | -1.0899011 | 1.83E-06 | 1.47E-05 | CD82 | protein_coding |
| ENSG00000280079 | 33.2321315 | 92.0069486 | -1.4726524 | 2.00E-06 | 1.60E-05 | AC011447.7 | TEC |
| ENSG00000276900 | 104.289654 | 40.8009219 | 1.35455671 | 2.02E-06 | 1.61E-05 | AC023157.3 | antisense |
| ENSG00000162241 | 156.683662 | 68.3593154 | 1.19315402 | 2.05E-06 | 1.63E-05 | SLC25A45 | protein_coding |
| ENSG00000149212 | 83.4211205 | 170.229432 | -1.0298311 | 2.22E-06 | 1.76E-05 | SESN3 | protein_coding |
| ENSG00000137101 | 74.0891855 | 19.3362205 | 1.92655366 | 2.30E-06 | 1.82E-05 | CD72 | protein_coding |
| ENSG00000229809 | 213.887814 | 106.604484 | 1.00301111 | 2.34E-06 | 1.84E-05 | ZNF688 | protein_coding |
| ENSG00000172382 | 103.339566 | 34.9099052 | 1.56344052 | 2.45E-06 | 1.92E-05 | PRSS27 | protein_coding |
| ENSG00000154553 | 77.5796159 | 165.160515 | -1.0927388 | 2.46E-06 | 1.94E-05 | PDLIM3 | protein_coding |
| ENSG00000267150 | 19.2122327 | 68.6685114 | -1.8376723 | 2.62E-06 | 2.05E-05 | AC006557.1 | antisense |
| ENSG00000143184 | 109.533566 | 44.1168652 | 1.31057909 | 2.86E-06 | 2.22E-05 | XCL1 | protein_coding |
| ENSG00000104883 | 115.396184 | 44.7889227 | 1.3632164 | 3.04E-06 | 2.35E-05 | PEX11G | protein_coding |
| ENSG00000189068 | 12.7628472 | 56.2535156 | -2.1373048 | 3.16E-06 | 2.44E-05 | VSTM1 | protein_coding |
| ENSG00000236675 | 225.477402 | 110.593909 | 1.02635172 | 3.42E-06 | 2.63E-05 | MTX1P1 | unprocessed_pseudogene |
| ENSG00000257576 | 74.632014 | 23.6648061 | 1.65299066 | 3.53E-06 | 2.71E-05 | HSPD1P4 | processed_pseudogene |
| ENSG00000204262 | 59.2234358 | 14.9857507 | 1.98154465 | 3.55E-06 | 2.72E-05 | COL5A2 | protein_coding |
| ENSG00000279821 | 93.1033433 | 32.6292737 | 1.50767622 | 3.63E-06 | 2.78E-05 | AC145098.2 | TEC |
| ENSG00000139410 | 73.8067648 | 21.2610572 | 1.79898073 | 4.06E-06 | 3.07E-05 | SDSL | protein_coding |
| ENSG00000148450 | 122.468803 | 47.4954404 | 1.37076779 | 4.15E-06 | 3.14E-05 | MSRB2 | protein_coding |
| ENSG00000171126 | 41.6163678 | 106.711213 | -1.3539647 | 4.16E-06 | 3.14E-05 | KCNG3 | protein_coding |
| ENSG00000139899 | 54.331223 | 11.92069 | 2.18769235 | 4.41E-06 | 3.31E-05 | CBLN3 | protein_coding |
| ENSG00000113296 | 85.0987079 | 24.5877462 | 1.78451832 | 4.50E-06 | 3.38E-05 | THBS4 | protein_coding |
| ENSG00000130203 | 123.456198 | 52.2531986 | 1.24370709 | 4.56E-06 | 3.42E-05 | APOE | protein_coding |
| ENSG00000235652 | 93.7146536 | 35.721259 | 1.39067067 | 4.63E-06 | 3.46E-05 | AL356599.1 | antisense |
| ENSG00000231770 | 182.968472 | 90.2857561 | 1.01611688 | 4.70E-06 | 3.51E-05 | TMEM44-AS1 | antisense |
| ENSG00000257702 | 43.8798398 | 6.63424208 | 2.70788319 | 5.74E-06 | 4.24E-05 | LBX2-AS1 | antisense |
| ENSG00000134317 | 43.2580429 | 108.149102 | -1.3249123 | 6.02E-06 | 4.44E-05 | GRHL1 | protein_coding |
| ENSG00000128573 | 145.201559 | 69.511254 | 1.06216487 | 6.22E-06 | 4.57E-05 | FOXP2 | protein_coding |
| ENSG00000232533 | 202.456691 | 100.669078 | 1.01012865 | 6.54E-06 | 4.79E-05 | AC093673.1 | antisense |
| ENSG00000270194 | 55.5117826 | 14.7384015 | 1.91807764 | 6.58E-06 | 4.82E-05 | AC097359.2 | antisense |
| ENSG00000270504 | 158.063691 | 72.0670078 | 1.12836396 | 6.70E-06 | 4.89E-05 | AL391422.4 | antisense |
| ENSG00000184508 | 95.6618273 | 37.3039405 | 1.36174786 | 6.75E-06 | 4.92E-05 | HDDC3 | protein_coding |
| ENSG00000100156 | 64.1408476 | 17.2461318 | 1.89392589 | 6.87E-06 | 5.00E-05 | SLC16A8 | protein_coding |
| ENSG00000226314 | 93.0172477 | 36.3572165 | 1.35573904 | 6.88E-06 | 5.01E-05 | ZNF192P1 | transcribed_unprocessed_pseudogene |
| ENSG00000280123 | 53.3062357 | 120.853436 | -1.1789675 | 7.03E-06 | 5.11E-05 | AC023632.6 | TEC |
| ENSG00000256043 | 133.891647 | 46.1201835 | 1.54184069 | 7.19E-06 | 5.22E-05 | CTSO | protein_coding |
| ENSG00000164845 | 85.8621611 | 31.9488259 | 1.42528737 | 7.19E-06 | 5.22E-05 | FAM86FP | transcribed_unprocessed_pseudogene |
| ENSG00000226711 | 86.2483777 | 31.6638064 | 1.44641321 | 7.20E-06 | 5.22E-05 | FAM66C | antisense |
| ENSG00000116819 | 113.497617 | 48.4128842 | 1.22915191 | 8.42E-06 | 6.06E-05 | TFAP2E | protein_coding |
| ENSG00000258366 | 70.6992081 | 155.170367 | -1.137 | 8.54E-06 | 6.14E-05 | RTEL1 | protein_coding |
| ENSG00000152467 | 84.1905493 | 30.2247396 | 1.47766867 | 8.66E-06 | 6.22E-05 | ZSCAN1 | protein_coding |
| ENSG00000079150 | 121.812133 | 53.567641 | 1.18459602 | 9.09E-06 | 6.49E-05 | FKBP7 | protein_coding |
| ENSG00000204540 | 86.1774197 | 32.2691603 | 1.41682942 | 9.20E-06 | 6.56E-05 | PSORS1C1 | protein_coding |
| ENSG00000273382 | 50.6769668 | 13.0107818 | 1.96604648 | 1.02E-05 | 7.21E-05 | AL356488.3 | antisense |
| ENSG00000162426 | 82.3362599 | 29.2784082 | 1.48646987 | 1.05E-05 | 7.41E-05 | SLC45A1 | protein_coding |
| ENSG00000161609 | 101.369513 | 31.5118647 | 1.67582629 | 1.12E-05 | 7.86E-05 | CCDC155 | protein_coding |
| ENSG00000115274 | 65.0771766 | 20.7351157 | 1.64871959 | 1.26E-05 | 8.77E-05 | INO80B | protein_coding |
| ENSG00000262691 | 203.358718 | 100.598803 | 1.01206281 | 1.34E-05 | 9.30E-05 | AC040160.1 | processed_transcript |
| ENSG00000116254 | 76.6598192 | 27.0168494 | 1.49980847 | 1.39E-05 | 9.63E-05 | CHD5 | protein_coding |
| ENSG00000271122 | 157.519199 | 78.5749918 | 1.00107357 | 1.40E-05 | 9.65E-05 | AC018647.2 | antisense |
| ENSG00000071282 | 71.4649391 | 145.973405 | -1.0324139 | 1.43E-05 | 9.88E-05 | LMCD1 | protein_coding |
| ENSG00000163009 | 56.876501 | 126.418622 | -1.1557022 | 1.56E-05 | 0.00010742 | C2orf48 | lincRNA |
| ENSG00000148057 | 105.982664 | 44.6734104 | 1.24882481 | 1.75E-05 | 0.00011947 | IDNK | protein_coding |
| ENSG00000176809 | 146.879501 | 68.468008 | 1.09732192 | 1.77E-05 | 0.00012055 | LRRC37A3 | protein_coding |
| ENSG00000213967 | 79.9986602 | 163.22768 | -1.0292518 | 1.79E-05 | 0.00012195 | ZNF726 | protein_coding |
| ENSG00000214575 | 127.478174 | 56.828019 | 1.16044015 | 1.87E-05 | 0.00012695 | CPEB1 | protein_coding |
| ENSG00000160951 | 94.7188661 | 36.0419859 | 1.39218525 | 1.91E-05 | 0.00012906 | PTGER1 | protein_coding |
| ENSG00000152763 | 81.5595307 | 30.3271506 | 1.42994785 | 1.97E-05 | 0.00013286 | WDR78 | protein_coding |
| ENSG00000204947 | 123.132929 | 58.357035 | 1.07975691 | 1.99E-05 | 0.0001344 | ZNF425 | protein_coding |
| ENSG00000285184 | 88.4040963 | 35.5759282 | 1.31570251 | 2.10E-05 | 0.00014103 | AC244033.2 | bidirectional_promoter_lncRNA |
| ENSG00000141505 | 113.031037 | 50.4227753 | 1.16586978 | 2.12E-05 | 0.00014238 | ASGR1 | protein_coding |
| ENSG00000227354 | 79.174649 | 28.3601793 | 1.47857036 | 2.12E-05 | 0.00014244 | RBM26-AS1 | antisense |
| ENSG00000187908 | 87.3791956 | 34.072659 | 1.35514785 | 2.20E-05 | 0.00014747 | DMBT1 | protein_coding |
| ENSG00000066230 | 53.4042125 | 15.0540247 | 1.8297645 | 2.22E-05 | 0.00014843 | SLC9A3 | protein_coding |
| ENSG00000256667 | 69.8321574 | 145.955055 | -1.0604167 | 2.23E-05 | 0.00014893 | KLRA1P | transcribed_unprocessed_pseudogene |
| ENSG00000204790 | 51.0454831 | 13.9992478 | 1.86876883 | 2.27E-05 | 0.0001516 | AL163540.1 | unprocessed_pseudogene |
| ENSG00000115556 | 60.0984267 | 127.123246 | -1.0817685 | 2.29E-05 | 0.00015298 | PLCD4 | protein_coding |
| ENSG00000272720 | 46.7518162 | 11.8163161 | 1.97836146 | 2.31E-05 | 0.00015399 | AL022322.1 | lincRNA |
| ENSG00000233184 | 146.606502 | 70.2460892 | 1.05959484 | 2.44E-05 | 0.00016222 | AC093157.1 | antisense |
| ENSG00000268902 | 257.557083 | 126.253186 | 1.02752056 | 2.48E-05 | 0.00016451 | CSAG2 | protein_coding |
| ENSG00000159761 | 125.210121 | 60.4479088 | 1.05037124 | 2.65E-05 | 0.00017526 | C16orf86 | protein_coding |
| ENSG00000261061 | 59.9523714 | 127.683964 | -1.0918447 | 2.76E-05 | 0.00018176 | AC092718.4 | sense_intronic |
| ENSG00000255306 | 44.090861 | 9.80367676 | 2.16473401 | 3.01E-05 | 0.00019677 | AC004923.4 | antisense |
| ENSG00000173227 | 72.0693907 | 26.1649314 | 1.46055731 | 3.03E-05 | 0.00019764 | SYT12 | protein_coding |
| ENSG00000233117 | 57.4673216 | 17.0317418 | 1.7577803 | 3.11E-05 | 0.00020266 | LINC00702 | lincRNA |
| ENSG00000248161 | 64.4081143 | 21.5858558 | 1.57279974 | 3.15E-05 | 0.00020487 | AC098487.1 | lincRNA |
| ENSG00000146859 | 91.7868599 | 185.33067 | -1.0117853 | 3.28E-05 | 0.0002127 | TMEM140 | protein_coding |
| ENSG00000174586 | 98.4384596 | 43.0938696 | 1.18986705 | 3.45E-05 | 0.00022328 | ZNF497 | protein_coding |
| ENSG00000114405 | 42.0546039 | 101.112675 | -1.266381 | 3.52E-05 | 0.00022695 | C3orf14 | protein_coding |
| ENSG00000110031 | 73.4002315 | 27.2926936 | 1.43029064 | 3.86E-05 | 0.00024715 | LPXN | protein_coding |
| ENSG00000196335 | 11.0811904 | 44.7202561 | -2.0232802 | 3.91E-05 | 0.00025023 | STK31 | protein_coding |
| ENSG00000285219 | 103.431125 | 45.2204509 | 1.18962288 | 3.92E-05 | 0.00025101 | AL591485.1 | bidirectional_promoter_lncRNA |
| ENSG00000235169 | 58.4007493 | 18.3731088 | 1.66732391 | 3.99E-05 | 0.00025496 | SMIM1 | protein_coding |
| ENSG00000152229 | 2.01979483 | 24.4753747 | -3.6419411 | 4.11E-05 | 0.00026242 | PSTPIP2 | protein_coding |
| ENSG00000185338 | 35.1274422 | 92.4895778 | -1.4018008 | 4.15E-05 | 0.00026483 | SOCS1 | protein_coding |
| ENSG00000176909 | 154.632779 | 71.2513355 | 1.11330153 | 4.23E-05 | 0.00026974 | MAMSTR | protein_coding |
| ENSG00000169583 | 50.9458266 | 15.3386517 | 1.73349606 | 4.29E-05 | 0.0002732 | CLIC3 | protein_coding |
| ENSG00000246273 | 104.300652 | 48.628452 | 1.10072607 | 4.71E-05 | 0.00029736 | SBF2-AS1 | antisense |
| ENSG00000128394 | 112.407136 | 52.9356094 | 1.08656613 | 4.79E-05 | 0.00030253 | APOBEC3F | protein_coding |
| ENSG00000137691 | 89.3411518 | 38.1868547 | 1.22804338 | 5.26E-05 | 0.00032971 | CFAP300 | protein_coding |
| ENSG00000000003 | 29.1634017 | 74.702896 | -1.3581264 | 5.54E-05 | 0.00034581 | TSPAN6 | protein_coding |
| ENSG00000275294 | 50.9901907 | 15.0218507 | 1.76400859 | 5.57E-05 | 0.00034756 | LINC02340 | lincRNA |
| ENSG00000181409 | 62.5736299 | 20.2778407 | 1.62226189 | 5.59E-05 | 0.00034875 | AATK | protein_coding |
| ENSG00000257176 | 62.2342537 | 21.6116026 | 1.53070312 | 5.84E-05 | 0.00036292 | AC009318.1 | antisense |
| ENSG00000102904 | 122.832098 | 57.6687985 | 1.08605422 | 6.25E-05 | 0.00038621 | TSNAXIP1 | protein_coding |
| ENSG00000170162 | 8.97864286 | 38.3610731 | -2.0932405 | 6.38E-05 | 0.00039321 | VGLL2 | protein_coding |
| ENSG00000168477 | 159.257284 | 61.2488713 | 1.37312802 | 6.39E-05 | 0.00039383 | TNXB | protein_coding |
| ENSG00000170498 | 1.64326825 | 20.8402748 | -3.6873196 | 6.40E-05 | 0.00039409 | KISS1 | protein_coding |
| ENSG00000260630 | 62.151501 | 20.4941937 | 1.59435923 | 6.44E-05 | 0.00039639 | SNAI3-AS1 | antisense |
| ENSG00000120324 | 86.1989044 | 34.662556 | 1.31502225 | 6.69E-05 | 0.00041032 | PCDHB10 | protein_coding |
| ENSG00000253669 | 106.353017 | 47.6653403 | 1.16048917 | 6.86E-05 | 0.00042034 | GASAL1 | lincRNA |
| ENSG00000160602 | 124.73199 | 60.975959 | 1.02957584 | 6.98E-05 | 0.0004269 | NEK8 | protein_coding |
| ENSG00000167216 | 94.7530287 | 41.4035277 | 1.19221331 | 7.32E-05 | 0.00044573 | KATNAL2 | protein_coding |
| ENSG00000250802 | 120.391412 | 59.1145395 | 1.02284313 | 7.59E-05 | 0.00046108 | ZBED3-AS1 | antisense |
| ENSG00000263327 | 60.0692867 | 19.6418831 | 1.61014579 | 7.69E-05 | 0.0004659 | TAPT1-AS1 | antisense |
| ENSG00000185267 | 46.2899906 | 11.7123347 | 1.97441455 | 8.14E-05 | 0.00049091 | CDNF | protein_coding |
| ENSG00000171522 | 21.132371 | 62.0742953 | -1.5542845 | 8.69E-05 | 0.000521 | PTGER4 | protein_coding |
| ENSG00000270605 | 75.6264666 | 30.8234194 | 1.29305779 | 9.23E-05 | 0.00055028 | AL353622.1 | antisense |
| ENSG00000196668 | 87.3496139 | 38.8585196 | 1.17045086 | 9.77E-05 | 0.00057866 | LINC00173 | processed_transcript |
| ENSG00000163661 | 55.3581584 | 111.440331 | -1.0112338 | 0.00010112 | 0.00059777 | PTX3 | protein_coding |
| ENSG00000279133 | 26.1917689 | 69.9971696 | -1.4161832 | 0.00010687 | 0.000629 | AC018628.1 | TEC |
| ENSG00000134955 | 119.290531 | 58.7255385 | 1.01909883 | 0.00010743 | 0.00063202 | SLC37A2 | protein_coding |
| ENSG00000167780 | 0 | 14.5146525 | -6.3612884 | 0.00010751 | 0.00063231 | SOAT2 | protein_coding |
| ENSG00000203799 | 120.557714 | 59.1510321 | 1.02469826 | 0.00010941 | 0.0006427 | CCDC162P | transcribed_unitary_pseudogene |
| ENSG00000148832 | 109.931136 | 51.1880685 | 1.10733649 | 0.00011276 | 0.00066069 | PAOX | protein_coding |
| ENSG00000158715 | 54.6182247 | 115.237333 | -1.0770962 | 0.00011369 | 0.00066581 | SLC45A3 | protein_coding |
| ENSG00000198286 | 0 | 13.9807133 | -6.3065556 | 0.0001228 | 0.00071362 | CARD11 | protein_coding |
| ENSG00000175319 | 27.2466062 | 68.7744558 | -1.336481 | 0.00012343 | 0.00071672 | NF1P5 | processed_pseudogene |
| ENSG00000197446 | 0 | 13.5948531 | -6.2668812 | 0.00012797 | 0.00074107 | CYP2F1 | protein_coding |
| ENSG00000229474 | 38.6350449 | 9.97671735 | 1.9616396 | 0.00014016 | 0.00080565 | PATL2 | protein_coding |
| ENSG00000244486 | 67.4518567 | 25.1764655 | 1.42391229 | 0.00014472 | 0.00082923 | SCARF2 | protein_coding |
| ENSG00000278974 | 35.4933257 | 82.283862 | -1.2126021 | 0.00014976 | 0.00085601 | AC093909.6 | TEC |
| ENSG00000171303 | 52.7262567 | 17.4513464 | 1.59954857 | 0.0001512 | 0.00086343 | KCNK3 | protein_coding |
| ENSG00000283959 | 42.7202369 | 11.2808065 | 1.92378177 | 0.00015121 | 0.00086343 | AP002851.1 | bidirectional_promoter_lncRNA |
| ENSG00000157343 | 41.6190006 | 11.3180843 | 1.88245486 | 0.00015287 | 0.00087204 | ARMC12 | protein_coding |
| ENSG00000204044 | 42.4796341 | 12.305765 | 1.78974566 | 0.00016576 | 0.00093758 | SLC12A5-AS1 | antisense |
| ENSG00000186364 | 109.016007 | 54.0686209 | 1.00884001 | 0.00017049 | 0.00096317 | NUDT17 | protein_coding |
| ENSG00000118514 | 31.5440575 | 6.35000771 | 2.30217667 | 0.00017388 | 0.00098181 | ALDH8A1 | protein_coding |
| ENSG00000236090 | 2.81502092 | 23.5306137 | -3.0458775 | 0.00017444 | 0.00098452 | LDHAP3 | processed_pseudogene |
| ENSG00000227855 | 41.2799795 | 90.8015914 | -1.135172 | 0.00017572 | 0.00099124 | DPY19L2P3 | transcribed_unprocessed_pseudogene |
| ENSG00000266208 | 25.0200162 | 3.80657003 | 2.73000381 | 0.00017593 | 0.00099198 | AC080112.1 | antisense |
| ENSG00000226174 | 74.4665086 | 31.9162593 | 1.22128488 | 0.000179 | 0.00100734 | TEX22 | protein_coding |
| ENSG00000143416 | 0 | 12.357075 | -6.1290965 | 0.00018157 | 0.00102005 | SELENBP1 | protein_coding |
| ENSG00000213853 | 41.8591619 | 91.4123404 | -1.1297235 | 0.00018479 | 0.00103689 | EMP2 | protein_coding |
| ENSG00000235560 | 47.2815254 | 14.3866783 | 1.71997648 | 0.00018522 | 0.00103884 | AC002310.1 | antisense |
| ENSG00000279253 | 91.6708277 | 42.3892455 | 1.11072245 | 0.00019567 | 0.00108981 | AL121753.2 | TEC |
| ENSG00000149150 | 1.28320389 | 21.4894794 | -4.0699123 | 0.00020779 | 0.0011546 | SLC43A1 | protein_coding |
| ENSG00000230454 | 33.9817553 | 8.04388302 | 2.06939258 | 0.00021178 | 0.00117423 | U73166.1 | lincRNA |
| ENSG00000125508 | 19.7997804 | 1.9765393 | 3.29655406 | 0.00022095 | 0.0012207 | SRMS | protein_coding |
| ENSG00000120549 | 91.2634114 | 38.7287914 | 1.23365745 | 0.00022161 | 0.00122379 | KIAA1217 | protein_coding |
| ENSG00000232859 | 48.4537196 | 16.2247066 | 1.57479063 | 0.00022307 | 0.00123096 | LYRM9 | protein_coding |
| ENSG00000065618 | 35.5414742 | 84.3747359 | -1.2521621 | 0.00022674 | 0.00124877 | COL17A1 | protein_coding |
| ENSG00000128165 | 50.8871914 | 17.6331698 | 1.52631352 | 0.00022776 | 0.00125286 | ADM2 | protein_coding |
| ENSG00000104901 | 81.813323 | 36.0725896 | 1.18068972 | 0.00022798 | 0.00125362 | DKKL1 | protein_coding |
| ENSG00000076641 | 12.0085559 | 41.1522525 | -1.7837415 | 0.00022837 | 0.00125517 | PAG1 | protein_coding |
| ENSG00000237310 | 42.9773019 | 12.7702524 | 1.74380399 | 0.00023419 | 0.00128532 | GS1-124K5.4 | lincRNA |
| ENSG00000232909 | 26.9692946 | 4.82838779 | 2.49160706 | 0.00023464 | 0.00128749 | AL157823.2 | antisense |
| ENSG00000054983 | 100.540054 | 46.6439151 | 1.11280329 | 0.00024299 | 0.00132989 | GALC | protein_coding |
| ENSG00000130513 | 62.0936624 | 22.777966 | 1.44713227 | 0.00024454 | 0.00133776 | GDF15 | protein_coding |
| ENSG00000265763 | 23.7405967 | 61.0754762 | -1.3623725 | 0.00025041 | 0.00136698 | ZNF488 | protein_coding |
| ENSG00000230699 | 47.1409341 | 14.6701275 | 1.68589487 | 0.0002515 | 0.00137123 | AL645608.2 | lincRNA |
| ENSG00000160712 | 118.88179 | 58.7826741 | 1.0171673 | 0.00025154 | 0.00137123 | IL6R | protein_coding |
| ENSG00000197815 | 96.8039983 | 46.8944051 | 1.04891058 | 0.00025398 | 0.00138385 | AC122129.1 | antisense |
| ENSG00000168676 | 126.624581 | 56.4017402 | 1.16261637 | 0.00025682 | 0.00139834 | KCTD19 | protein_coding |
| ENSG00000254815 | 63.0548355 | 24.5750375 | 1.36189253 | 0.00025889 | 0.00140848 | AP006284.1 | antisense |
| ENSG00000178809 | 63.3461331 | 23.9053355 | 1.40623286 | 0.00025892 | 0.00140848 | TRIM73 | protein_coding |
| ENSG00000128298 | 55.7794907 | 15.2101013 | 1.86048064 | 0.00025998 | 0.00141361 | BAIAP2L2 | protein_coding |
| ENSG00000255262 | 61.6064812 | 22.779929 | 1.43751976 | 0.00026094 | 0.00141849 | ELOBP2 | processed_pseudogene |
| ENSG00000174837 | 0 | 11.5734311 | -6.0326255 | 0.00026125 | 0.00141984 | ADGRE1 | protein_coding |
| ENSG00000164796 | 26.4762943 | 5.07887777 | 2.37739809 | 0.00026449 | 0.00143475 | CSMD3 | protein_coding |
| ENSG00000273230 | 78.4545203 | 32.3405751 | 1.28028032 | 0.00026789 | 0.00145172 | AC102953.2 | lincRNA |
| ENSG00000163545 | 52.3662789 | 108.847299 | -1.0553219 | 0.00026913 | 0.00145791 | NUAK2 | protein_coding |
| ENSG00000107611 | 63.2434888 | 23.6242419 | 1.42374028 | 0.00027107 | 0.00146706 | CUBN | protein_coding |
| ENSG00000115339 | 31.4808414 | 6.20546212 | 2.34937011 | 0.00028258 | 0.00152475 | GALNT3 | protein_coding |
| ENSG00000272438 | 17.8335982 | 1.30644474 | 3.73270044 | 0.00028493 | 0.0015353 | AL645608.6 | lincRNA |
| ENSG00000223764 | 82.4190126 | 30.6502332 | 1.42767322 | 0.00028732 | 0.00154636 | LINC02593 | lincRNA |
| ENSG00000276672 | 56.6514939 | 116.305604 | -1.0367562 | 0.00028803 | 0.00154986 | AL161891.1 | sense_intronic |
| ENSG00000186765 | 51.7532889 | 18.0217782 | 1.52283788 | 0.00029365 | 0.00157682 | FSCN2 | protein_coding |
| ENSG00000204311 | 72.7346686 | 30.0095644 | 1.27668776 | 0.00029466 | 0.00158188 | PJVK | protein_coding |
| ENSG00000126583 | 122.242429 | 59.6810452 | 1.02939623 | 0.00029556 | 0.00158562 | PRKCG | protein_coding |
| ENSG00000262903 | 59.8366942 | 22.6084588 | 1.39936717 | 0.00029651 | 0.00159036 | AC027796.4 | antisense |
| ENSG00000184226 | 39.4843252 | 86.3309614 | -1.1253075 | 0.00030534 | 0.00163396 | PCDH9 | protein_coding |
| ENSG00000139438 | 20.9585002 | 55.3325385 | -1.4038154 | 0.00030721 | 0.00164319 | FAM222A | protein_coding |
| ENSG00000260563 | 79.9298069 | 34.1321503 | 1.22905253 | 0.00030753 | 0.00164427 | AC132872.1 | lincRNA |
| ENSG00000163701 | 12.1908785 | 44.3857059 | -1.8681452 | 0.00030954 | 0.00165376 | IL17RE | protein_coding |
| ENSG00000111348 | 1.65973048 | 18.8372035 | -3.5370296 | 0.00031195 | 0.00166434 | ARHGDIB | protein_coding |
| ENSG00000165905 | 66.1551784 | 27.5404353 | 1.26519235 | 0.0003179 | 0.00169376 | LARGE2 | protein_coding |
| ENSG00000269737 | 79.4844436 | 35.0503792 | 1.18274107 | 0.00032501 | 0.00172928 | AL691432.1 | transcribed_processed_pseudogene |
| ENSG00000107742 | 43.2411391 | 13.5090136 | 1.67295248 | 0.00032953 | 0.00175091 | SPOCK2 | protein_coding |
| ENSG00000266904 | 97.0361492 | 45.4313075 | 1.09065201 | 0.00033122 | 0.00175954 | LINC00663 | lincRNA |
| ENSG00000213888 | 58.8497404 | 21.5452916 | 1.45437296 | 0.0003476 | 0.00183901 | LINC01521 | lincRNA |
| ENSG00000197714 | 48.4958925 | 98.0385849 | -1.0137009 | 0.0003483 | 0.00184189 | ZNF460 | protein_coding |
| ENSG00000184108 | 38.606788 | 10.0898741 | 1.9307552 | 0.00036126 | 0.0019035 | TRIML1 | protein_coding |
| ENSG00000177406 | 44.8109897 | 13.4756618 | 1.72740582 | 0.00038886 | 0.00203786 | AC021054.1 | antisense |
| ENSG00000262580 | 95.4998378 | 42.7866366 | 1.15340879 | 0.00039882 | 0.00208492 | AC087741.1 | antisense |
| ENSG00000205771 | 51.1045598 | 16.4390966 | 1.6304576 | 0.00040433 | 0.00211089 | CATSPER2P1 | transcribed_unprocessed_pseudogene |
| ENSG00000276728 | 27.258046 | 4.51276459 | 2.60616692 | 0.00040766 | 0.00212682 | AC142472.1 | lincRNA |
| ENSG00000198416 | 46.0865383 | 15.7252971 | 1.55484731 | 0.00041027 | 0.0021376 | ZNF658B | transcribed_unprocessed_pseudogene |
| ENSG00000171056 | 43.2833121 | 89.1868815 | -1.0436008 | 0.00041276 | 0.00214957 | SOX7 | protein_coding |
| ENSG00000151320 | 71.5902197 | 31.5982806 | 1.18043858 | 0.00041649 | 0.00216708 | AKAP6 | protein_coding |
| ENSG00000111837 | 56.9975557 | 115.301497 | -1.017752 | 0.0004253 | 0.00220994 | MAK | protein_coding |
| ENSG00000181781 | 49.5406849 | 15.9730389 | 1.63063166 | 0.00043273 | 0.00224558 | ODF3L2 | protein_coding |
| ENSG00000167608 | 81.2844104 | 36.7907077 | 1.13973841 | 0.0004439 | 0.00229791 | TMC4 | protein_coding |
| ENSG00000176244 | 14.6171366 | 45.1486435 | -1.6242176 | 0.00046614 | 0.00240293 | ACBD7 | protein_coding |
| ENSG00000256268 | 44.5635281 | 14.7023015 | 1.60472599 | 0.00046859 | 0.00241339 | LINC02454 | lincRNA |
| ENSG00000250132 | 75.6413357 | 29.4904426 | 1.35144127 | 0.00046887 | 0.00241432 | AC004803.1 | antisense |
| ENSG00000271524 | 5.74994495 | 26.2343832 | -2.1864634 | 0.00048231 | 0.00248025 | BNIP3P17 | processed_pseudogene |
| ENSG00000255521 | 34.1151328 | 9.23756358 | 1.89005105 | 0.00049045 | 0.0025209 | AL356215.1 | antisense |
| ENSG00000242759 | 35.3975401 | 9.87469894 | 1.84190615 | 0.00049074 | 0.00252136 | LINC00882 | lincRNA |
| ENSG00000132000 | 41.0153457 | 11.2911597 | 1.84842729 | 0.00051449 | 0.00263355 | PODNL1 | protein_coding |
| ENSG00000160963 | 0 | 10.3809283 | -5.8782053 | 0.00051903 | 0.00265154 | COL26A1 | protein_coding |
| ENSG00000066056 | 1.91256956 | 23.5438607 | -3.6213922 | 0.0005361 | 0.00273334 | TIE1 | protein_coding |
| ENSG00000175175 | 41.3838619 | 88.6509794 | -1.0993168 | 0.00054111 | 0.00275707 | PPM1E | protein_coding |
| ENSG00000128045 | 0 | 8.56707642 | -5.5938738 | 0.00054144 | 0.00275816 | RASL11B | protein_coding |
| ENSG00000270419 | 15.7716305 | 1.34058174 | 3.54137668 | 0.00054328 | 0.00276694 | CAHM | lincRNA |
| ENSG00000181856 | 20.5321454 | 55.1345362 | -1.4279317 | 0.00054894 | 0.00279213 | SLC2A4 | protein_coding |
| ENSG00000203362 | 19.6748548 | 52.5140418 | -1.4202201 | 0.00055187 | 0.00280334 | POLH-AS1 | antisense |
| ENSG00000275481 | 43.9670869 | 14.0759119 | 1.63677788 | 0.00057886 | 0.0029283 | AC025031.4 | lincRNA |
| ENSG00000254198 | 44.6653759 | 93.2065181 | -1.0648686 | 0.00058008 | 0.0029332 | AC113191.1 | processed_pseudogene |
| ENSG00000283709 | 33.6967883 | 8.14825702 | 2.04886031 | 0.00058317 | 0.00294818 | FAM238C | transcribed_unprocessed_pseudogene |
| ENSG00000273786 | 1.94971996 | 16.9638604 | -3.1327326 | 0.0005996 | 0.00302537 | AC020658.4 | sense_intronic |
| ENSG00000165443 | 14.3562872 | 42.7019749 | -1.5683679 | 0.00060371 | 0.00304347 | PHYHIPL | protein_coding |
| ENSG00000171388 | 3.53937557 | 20.951076 | -2.569113 | 0.00060696 | 0.00305852 | APLN | protein_coding |
| ENSG00000166349 | 59.9321248 | 24.3622179 | 1.30208314 | 0.00061628 | 0.0031035 | RAG1 | protein_coding |
| ENSG00000213889 | 71.4458441 | 32.3025121 | 1.14665873 | 0.00062556 | 0.00314548 | PPM1N | protein_coding |
| ENSG00000225980 | 0.60445154 | 14.3164413 | -4.4991059 | 0.00063392 | 0.00318134 | OR7E19P | transcribed_unprocessed_pseudogene |
| ENSG00000261971 | 103.534566 | 50.2581249 | 1.03892949 | 0.00063579 | 0.00318866 | MMP25-AS1 | antisense |
| ENSG00000225096 | 82.7959808 | 39.2424168 | 1.07864404 | 0.00064337 | 0.00321973 | AL445250.1 | lincRNA |
| ENSG00000150054 | 40.1787432 | 82.7003258 | -1.0433201 | 0.00064381 | 0.00322127 | MPP7 | protein_coding |
| ENSG00000215845 | 0 | 9.46152154 | -5.7442959 | 0.00064401 | 0.00322156 | TSTD1 | protein_coding |
| ENSG00000175463 | 40.2960135 | 11.7127273 | 1.77614432 | 0.00065 | 0.00325015 | TBC1D10C | protein_coding |
| ENSG00000249328 | 40.342924 | 11.1128697 | 1.8537262 | 0.00066851 | 0.00333339 | AC036214.1 | lincRNA |
| ENSG00000198723 | 53.1192455 | 20.1317247 | 1.40206874 | 0.00069226 | 0.00343931 | TEX45 | protein_coding |
| ENSG00000267704 | 22.7646411 | 60.0224153 | -1.392713 | 0.00069623 | 0.0034561 | FRG2LP | processed_pseudogene |
| ENSG00000137968 | 87.3998837 | 42.6424836 | 1.03366523 | 0.00071138 | 0.00352532 | SLC44A5 | protein_coding |
| ENSG00000172995 | 31.8953149 | 8.21378284 | 1.9601601 | 0.00071314 | 0.00353254 | ARPP21 | protein_coding |
| ENSG00000228624 | 91.8729554 | 44.294617 | 1.05297672 | 0.00071943 | 0.00356068 | HDAC2-AS2 | antisense |
| ENSG00000169436 | 0 | 8.96171938 | -5.6638138 | 0.00072516 | 0.00358596 | COL22A1 | protein_coding |
| ENSG00000282386 | 42.0781934 | 13.827385 | 1.60053496 | 0.00073125 | 0.0036115 | AL358472.4 | antisense |
| ENSG00000213742 | 57.3909861 | 22.4719108 | 1.34591752 | 0.00073806 | 0.00364203 | ZNF337-AS1 | antisense |
| ENSG00000260267 | 39.268195 | 12.6578808 | 1.63790384 | 0.00074702 | 0.00367896 | AC026471.1 | antisense |
| ENSG00000257524 | 46.6859673 | 15.9726463 | 1.54621147 | 0.00075668 | 0.00372448 | AL157935.2 | protein_coding |
| ENSG00000262468 | 84.4257746 | 41.3697833 | 1.02652496 | 0.00075787 | 0.00372875 | LINC01569 | lincRNA |
| ENSG00000187372 | 25.8017679 | 5.81724634 | 2.1520474 | 0.00077426 | 0.00379899 | PCDHB13 | protein_coding |
| ENSG00000177374 | 72.9887458 | 31.6043151 | 1.20191028 | 0.00078171 | 0.00382989 | HIC1 | protein_coding |
| ENSG00000128438 | 0 | 8.85970097 | -5.6490065 | 0.00078317 | 0.00383626 | TBC1D27P | transcribed_unprocessed_pseudogene |
| ENSG00000234028 | 38.1263789 | 11.8496679 | 1.68566966 | 0.00080286 | 0.00392612 | AC062029.1 | antisense |
| ENSG00000213417 | 6.27507316 | 30.3870344 | -2.2894001 | 0.00081612 | 0.00398762 | KRTAP2-4 | protein_coding |
| ENSG00000168517 | 47.2448165 | 17.9856782 | 1.39160678 | 0.00085098 | 0.0041458 | HEXIM2 | protein_coding |
| ENSG00000207973 | 6.73514902 | 27.428849 | -2.0237288 | 0.00085387 | 0.00415899 | MIR589 | miRNA |
| ENSG00000133256 | 49.5055691 | 17.7451488 | 1.47321545 | 0.00086232 | 0.00419575 | PDE6B | protein_coding |
| ENSG00000267390 | 20.5321454 | 3.13726066 | 2.72867538 | 0.00086652 | 0.00421525 | AC036176.1 | antisense |
| ENSG00000279267 | 66.8584899 | 29.4744475 | 1.18491276 | 0.00086669 | 0.00421525 | AL078621.3 | TEC |
| ENSG00000270959 | 64.842921 | 28.1760003 | 1.20148619 | 0.00089535 | 0.00433659 | LPP-AS2 | antisense |
| ENSG00000270607 | 26.335348 | 66.5946649 | -1.3450086 | 0.00089817 | 0.00434934 | AC009549.1 | lincRNA |
| ENSG00000272142 | 73.7046486 | 34.4589118 | 1.09278929 | 0.00090532 | 0.00438303 | LYRM4-AS1 | lincRNA |
| ENSG00000246982 | 48.2274743 | 16.784 | 1.52735685 | 0.00090694 | 0.00438996 | Z84485.1 | antisense |
| ENSG00000049540 | 1.60611784 | 18.3545743 | -3.5211358 | 0.00090946 | 0.00440037 | ELN | protein_coding |
| ENSG00000284691 | 80.8488071 | 39.2539478 | 1.03893637 | 0.00093115 | 0.00449785 | AC073111.5 | protein_coding |
| ENSG00000177685 | 52.2454927 | 21.0527018 | 1.31068555 | 0.00093258 | 0.00450289 | CRACR2B | protein_coding |
| ENSG00000255135 | 74.6155518 | 35.755396 | 1.06125512 | 0.00093446 | 0.00451007 | AP002360.1 | lincRNA |
| ENSG00000234899 | 58.9274706 | 25.0073508 | 1.23382295 | 0.00095185 | 0.00458737 | SOX9-AS1 | processed_transcript |
| ENSG00000284681 | 13.1431581 | 38.8608752 | -1.5629975 | 0.00095602 | 0.00460366 | AC007240.1 | protein_coding |
| ENSG00000114737 | 6.66084821 | 27.8504166 | -2.054328 | 0.00097333 | 0.00468119 | CISH | protein_coding |
| ENSG00000237840 | 40.0622695 | 13.011567 | 1.62456442 | 0.00098246 | 0.00472219 | FAM21FP | transcribed_unprocessed_pseudogene |
| ENSG00000099866 | 52.3599317 | 19.6009263 | 1.42121724 | 0.00099192 | 0.0047657 | MADCAM1 | protein_coding |
| ENSG00000167483 | 3.83737539 | 27.2472345 | -2.8306508 | 0.0009942 | 0.00477566 | FAM129C | protein_coding |
| ENSG00000204969 | 35.562959 | 11.1458289 | 1.67007928 | 0.00100922 | 0.00484088 | PCDHA2 | protein_coding |
| ENSG00000158373 | 58.3871883 | 23.3805717 | 1.31729195 | 0.00104509 | 0.00499853 | HIST1H2BD | protein_coding |
| ENSG00000131398 | 42.7362575 | 14.1775378 | 1.58753181 | 0.00106266 | 0.00507214 | KCNC3 | protein_coding |
| ENSG00000271452 | 6.91747167 | 28.7019419 | -2.0398966 | 0.00106507 | 0.00508093 | AC005034.5 | sense_intronic |
| ENSG00000279989 | 59.5361481 | 25.2904074 | 1.23153351 | 0.00106647 | 0.00508618 | AC011815.3 | TEC |
| ENSG00000269516 | 0 | 8.89305278 | -5.653387 | 0.00107969 | 0.00514605 | CYP4F23P | transcribed_unprocessed_pseudogene |
| ENSG00000104826 | 61.0662855 | 26.6555582 | 1.19783676 | 0.00108675 | 0.00517443 | LHB | protein_coding |
| ENSG00000273521 | 11.3006635 | 38.7032283 | -1.7762372 | 0.00109774 | 0.00521932 | AL162274.1 | antisense |
| ENSG00000123977 | 28.3111336 | 63.5474991 | -1.1626137 | 0.00112008 | 0.00531253 | DAW1 | protein_coding |
| ENSG00000235363 | 12.4437176 | 43.0498353 | -1.7914711 | 0.00114894 | 0.00543722 | SNRPGP10 | processed_pseudogene |
| ENSG00000203943 | 80.0829195 | 35.6561258 | 1.16948614 | 0.0011496 | 0.00543928 | SAMD13 | protein_coding |
| ENSG00000221990 | 57.5235671 | 24.3614327 | 1.24357671 | 0.001155 | 0.0054626 | EXOC3-AS1 | antisense |
| ENSG00000124232 | 0.62513972 | 15.6392738 | -4.6240815 | 0.00115812 | 0.00547626 | RBPJL | protein_coding |
| ENSG00000233452 | 37.0533996 | 11.7817865 | 1.64823217 | 0.00116594 | 0.00550877 | STXBP5-AS1 | antisense |
| ENSG00000107796 | 70.9279296 | 30.2787345 | 1.2337252 | 0.00117213 | 0.00553686 | ACTA2 | protein_coding |
| ENSG00000246095 | 80.3954334 | 39.9260054 | 1.0078247 | 0.00118017 | 0.00556695 | LINC01096 | lincRNA |
| ENSG00000145949 | 46.1152368 | 16.440667 | 1.48145965 | 0.001181 | 0.00556974 | MYLK4 | protein_coding |
| ENSG00000270049 | 83.7575788 | 37.2194877 | 1.16315759 | 0.00121058 | 0.00569771 | AC009061.2 | antisense |
| ENSG00000150510 | 20.7271459 | 51.6560893 | -1.3128742 | 0.00122521 | 0.00575613 | FAM124A | protein_coding |
| ENSG00000091490 | 75.0245613 | 36.4961201 | 1.03998743 | 0.00122983 | 0.00577552 | SEL1L3 | protein_coding |
| ENSG00000179528 | 43.8493751 | 15.5875713 | 1.48899059 | 0.00124386 | 0.00583084 | LBX2 | protein_coding |
| ENSG00000167600 | 0.33937618 | 14.3665735 | -5.3832228 | 0.00125058 | 0.00585644 | CYP2S1 | protein_coding |
| ENSG00000253931 | 39.4830871 | 13.5761098 | 1.53886385 | 0.00125186 | 0.00586128 | AC105118.1 | antisense |
| ENSG00000147234 | 0 | 8.22335081 | -5.5410798 | 0.00125489 | 0.0058731 | FRMPD3 | protein_coding |
| ENSG00000232653 | 65.9340257 | 27.6007118 | 1.26188823 | 0.00126915 | 0.00593388 | GOLGA8N | protein_coding |
| ENSG00000182327 | 59.382879 | 26.0633056 | 1.18692537 | 0.00127538 | 0.00595825 | GLTPD2 | protein_coding |
| ENSG00000005108 | 12.7873197 | 0.67048717 | 4.23836182 | 0.00130019 | 0.00606686 | THSD7A | protein_coding |
| ENSG00000138028 | 80.6175393 | 39.5377896 | 1.02365419 | 0.00132502 | 0.00616788 | CGREF1 | protein_coding |
| ENSG00000271826 | 30.7572833 | 9.20342658 | 1.74247347 | 0.00134098 | 0.00623476 | PLS3-AS1 | antisense |
| ENSG00000242732 | 60.6500622 | 26.9409704 | 1.17342263 | 0.00137347 | 0.00636931 | RTL5 | protein_coding |
| ENSG00000165359 | 56.3534075 | 23.7703579 | 1.24653835 | 0.00140938 | 0.00652156 | INTS6L | protein_coding |
| ENSG00000255153 | 30.3431647 | 8.8167812 | 1.78233071 | 0.00142143 | 0.00657212 | TOLLIP-AS1 | antisense |
| ENSG00000109832 | 0 | 8.29123222 | -5.5514931 | 0.00145212 | 0.00670206 | DDX25 | protein_coding |
| ENSG00000213397 | 13.4124595 | 39.1343638 | -1.5400527 | 0.00145556 | 0.00671661 | HAUS7 | protein_coding |
| ENSG00000180914 | 8.80855649 | 31.1096168 | -1.8313293 | 0.00146287 | 0.00674634 | OXTR | protein_coding |
| ENSG00000140678 | 0.30222577 | 10.7909649 | -4.9677749 | 0.00147138 | 0.00678023 | ITGAX | protein_coding |
| ENSG00000248932 | 52.1382674 | 21.8256 | 1.25852661 | 0.00148843 | 0.00684931 | AC097103.2 | antisense |
| ENSG00000129451 | 0 | 7.90537202 | -5.4840565 | 0.0015033 | 0.00690865 | KLK10 | protein_coding |
| ENSG00000129646 | 69.9542518 | 32.5414711 | 1.10624454 | 0.0015034 | 0.00690865 | QRICH2 | protein_coding |
| ENSG00000142609 | 1.30811802 | 19.2731959 | -3.898966 | 0.00150386 | 0.00690941 | CFAP74 | protein_coding |
| ENSG00000134215 | 7.9812025 | 28.4177075 | -1.8274338 | 0.00150829 | 0.0069284 | VAV3 | protein_coding |
| ENSG00000262576 | 68.75794 | 33.2568411 | 1.0463496 | 0.00153079 | 0.00701795 | PCDHGA4 | protein_coding |
| ENSG00000277342 | 12.7788678 | 37.4131713 | -1.5475843 | 0.00155993 | 0.0071347 | AC048344.4 | sense_intronic |
| ENSG00000167798 | 0.64160195 | 17.5122243 | -4.7752393 | 0.00156225 | 0.0071397 | C3P1 | transcribed_unprocessed_pseudogene |
| ENSG00000111859 | 0 | 7.40635506 | -5.3868711 | 0.00157486 | 0.00719593 | NEDD9 | protein_coding |
| ENSG00000138741 | 18.7981141 | 47.7166502 | -1.3417553 | 0.00157547 | 0.0071973 | TRPC3 | protein_coding |
| ENSG00000273145 | 44.2605058 | 16.5339027 | 1.42733048 | 0.00158684 | 0.0072464 | BX537318.1 | lincRNA |
| ENSG00000272070 | 32.9712821 | 9.97632476 | 1.72946699 | 0.00159399 | 0.0072705 | AC005618.1 | lincRNA |
| ENSG00000282381 | 1.8711932 | 15.7678243 | -3.0548105 | 0.00162032 | 0.00737818 | AC104073.4 | lincRNA |
| ENSG00000253438 | 0 | 7.37221806 | -5.3809754 | 0.00162387 | 0.00739092 | PCAT1 | antisense |
| ENSG00000278048 | 0.33937618 | 9.80485455 | -4.8292679 | 0.00163254 | 0.00742314 | RF00004 | snRNA |
| ENSG00000175267 | 0.33937618 | 10.3026937 | -4.9035706 | 0.00168512 | 0.00764283 | VWA3A | protein_coding |
| ENSG00000060709 | 0 | 8.72236777 | -5.6273522 | 0.00171946 | 0.00777742 | RIMBP2 | protein_coding |
| ENSG00000268942 | 17.7420386 | 45.0390201 | -1.3480459 | 0.00172944 | 0.007818 | CKS1BP3 | processed_pseudogene |
| ENSG00000164418 | 22.3374897 | 5.04434817 | 2.14028794 | 0.00175402 | 0.00791992 | GRIK2 | protein_coding |
| ENSG00000259660 | 63.5646531 | 27.3379689 | 1.2160489 | 0.00179431 | 0.00808162 | DNM1P47 | transcribed_unprocessed_pseudogene |
| ENSG00000120217 | 24.1909825 | 56.80692 | -1.2341705 | 0.00181062 | 0.00813766 | CD274 | protein_coding |
| ENSG00000283529 | 0.60445154 | 15.8207046 | -4.6591168 | 0.0018117 | 0.00814098 | AL035685.1 | transcribed_unprocessed_pseudogene |
| ENSG00000247796 | 60.2094364 | 25.9893896 | 1.21373219 | 0.00183122 | 0.00821917 | AC008966.1 | antisense |
| ENSG00000251661 | 44.890668 | 16.2906251 | 1.46307039 | 0.00183404 | 0.00822865 | AC136475.1 | antisense |
| ENSG00000196154 | 6.30078385 | 25.5666442 | -2.0089275 | 0.00183441 | 0.00822875 | S100A4 | protein_coding |
| ENSG00000275183 | 45.708332 | 17.5215834 | 1.3876374 | 0.00183946 | 0.00824823 | LENG9 | protein_coding |
| ENSG00000267065 | 27.605874 | 5.19046415 | 2.38320265 | 0.00186992 | 0.00837515 | LINC02080 | lincRNA |
| ENSG00000105357 | 0.98520407 | 17.8535944 | -4.2006297 | 0.00187686 | 0.00840263 | MYH14 | protein_coding |
| ENSG00000143494 | 36.6494127 | 79.418127 | -1.1102393 | 0.00187714 | 0.00840263 | VASH2 | protein_coding |
| ENSG00000279039 | 9.59154629 | 31.8563755 | -1.7331888 | 0.00187854 | 0.00840729 | AC011447.6 | TEC |
| ENSG00000172578 | 0 | 7.02088747 | -5.3110294 | 0.00190353 | 0.00849792 | KLHL6 | protein_coding |
| ENSG00000177455 | 1.58542966 | 17.8543796 | -3.488768 | 0.0019302 | 0.00860055 | CD19 | protein_coding |
| ENSG00000228589 | 28.0877896 | 68.2110908 | -1.2744632 | 0.00198449 | 0.00881716 | SPCS2P4 | processed_pseudogene |
| ENSG00000267383 | 26.0005528 | 57.4555863 | -1.1459887 | 0.00198499 | 0.00881774 | AC011447.3 | antisense |
| ENSG00000178078 | 59.3816409 | 21.5593238 | 1.45199477 | 0.00199574 | 0.00886211 | STAP2 | protein_coding |
| ENSG00000177694 | 33.4435942 | 9.98510753 | 1.73154285 | 0.00200831 | 0.00890945 | NAALADL2 | protein_coding |
| ENSG00000122420 | 37.7245832 | 13.6114246 | 1.47195948 | 0.00202867 | 0.0089895 | PTGFR | protein_coding |
| ENSG00000135253 | 2.90199957 | 21.3890314 | -2.8889739 | 0.00203433 | 0.00901288 | KCP | protein_coding |
| ENSG00000159110 | 28.8194446 | 65.1933509 | -1.1806895 | 0.00205327 | 0.0090899 | IFNAR2 | protein_coding |
| ENSG00000108785 | 32.0611754 | 10.5098713 | 1.60739525 | 0.00206774 | 0.00914699 | HSD17B1P1 | unprocessed_pseudogene |
| ENSG00000259803 | 78.3537123 | 39.0142036 | 1.00081017 | 0.00208253 | 0.00920602 | SLC22A31 | protein_coding |
| ENSG00000174343 | 0 | 7.80296102 | -5.4666891 | 0.00209057 | 0.00923571 | CHRNA9 | protein_coding |
| ENSG00000197180 | 30.2528433 | 8.9187996 | 1.76888711 | 0.0020998 | 0.00927124 | CH17-340M24.3 | lincRNA |
| ENSG00000108984 | 2.88553734 | 22.4795157 | -2.9642058 | 0.00212191 | 0.00935998 | MAP2K6 | protein_coding |
| ENSG00000089820 | 44.4592907 | 17.8054252 | 1.32458699 | 0.00214311 | 0.00943923 | ARHGAP4 | protein_coding |
| ENSG00000013619 | 17.8779624 | 47.7375402 | -1.4208916 | 0.00215044 | 0.00946975 | MAMLD1 | protein_coding |
| ENSG00000224080 | 13.5817493 | 37.9030129 | -1.483844 | 0.00216541 | 0.00952665 | UBE2FP1 | processed_pseudogene |
| ENSG00000168350 | 29.8020159 | 7.30355146 | 2.01787425 | 0.00219441 | 0.00964334 | DEGS2 | protein_coding |
| ENSG00000248554 | 21.3801876 | 4.76050638 | 2.16468443 | 0.00220992 | 0.00970418 | AC114956.2 | antisense |
| ENSG00000082684 | 0.33937618 | 12.9581104 | -5.2351601 | 0.00221666 | 0.0097307 | SEMA5B | protein_coding |
| ENSG00000167701 | 37.8321635 | 12.3824292 | 1.60496701 | 0.00222131 | 0.00974685 | GPT | protein_coding |
| ENSG00000269845 | 12.0300406 | 35.8574144 | -1.567591 | 0.00227229 | 0.0099346 | AC092364.2 | transcribed_processed_pseudogene |
| ENSG00000219626 | 53.4595048 | 22.2555578 | 1.26082747 | 0.00230892 | 0.01008765 | FAM228B | protein_coding |
| ENSG00000285886 | 19.8120167 | 4.12415622 | 2.27234814 | 0.00251019 | 0.01088948 | AC211476.6 | lincRNA |
| ENSG00000249626 | 65.708222 | 31.630062 | 1.05507445 | 0.00252139 | 0.01093397 | AC024560.2 | processed_pseudogene |
| ENSG00000181143 | 1.24605348 | 18.1144375 | -3.8457385 | 0.00253003 | 0.0109572 | MUC16 | protein_coding |
| ENSG00000240399 | 14.8033302 | 40.6234171 | -1.4569164 | 0.00262309 | 0.01132659 | AC004801.2 | transcribed_processed_pseudogene |
| ENSG00000164898 | 20.2299196 | 4.4774498 | 2.1842036 | 0.00266114 | 0.01148026 | FMC1 | protein_coding |
| ENSG00000259683 | 24.8460589 | 5.08044816 | 2.28882972 | 0.00266541 | 0.01149653 | AC243562.2 | unprocessed_pseudogene |
| ENSG00000158406 | 53.8305673 | 24.1205107 | 1.15635386 | 0.00266701 | 0.01150134 | HIST1H4H | protein_coding |
| ENSG00000105392 | 0.33937618 | 10.5170837 | -4.9339937 | 0.00271132 | 0.01166944 | CRX | protein_coding |
| ENSG00000183305 | 44.2956215 | 15.7927859 | 1.48920219 | 0.00271437 | 0.01167967 | MAGEA2B | protein_coding |
| ENSG00000227398 | 83.7031862 | 40.6881578 | 1.04204919 | 0.00271683 | 0.01168809 | KIF9-AS1 | antisense |
| ENSG00000221821 | 64.4904254 | 31.9146889 | 1.01399141 | 0.00274502 | 0.01180499 | C6orf226 | protein_coding |
| ENSG00000175084 | 0 | 6.66798649 | -5.2370041 | 0.00277332 | 0.01191352 | DES | protein_coding |
| ENSG00000267147 | 52.005773 | 23.1685373 | 1.16728934 | 0.00277934 | 0.01193721 | LINC01842 | lincRNA |
| ENSG00000159618 | 0.33937618 | 12.5718576 | -5.1918618 | 0.00280439 | 0.01203042 | ADGRG5 | protein_coding |
| ENSG00000177103 | 0.32291395 | 9.81402992 | -4.8355772 | 0.00280442 | 0.01203042 | DSCAML1 | protein_coding |
| ENSG00000116544 | 43.4533984 | 88.321571 | -1.0197547 | 0.00283452 | 0.01214956 | DLGAP3 | protein_coding |
| ENSG00000163624 | 34.2383787 | 69.053979 | -1.0123135 | 0.00284223 | 0.01217814 | CDS1 | protein_coding |
| ENSG00000250564 | 66.6136611 | 32.0841962 | 1.05481544 | 0.00285037 | 0.01221078 | AC109454.3 | lincRNA |
| ENSG00000140092 | 0.33937618 | 12.2880158 | -5.1587948 | 0.00286057 | 0.01224997 | FBLN5 | protein_coding |
| ENSG00000117586 | 22.9289084 | 4.97646676 | 2.18750455 | 0.0029669 | 0.01266347 | TNFSF4 | protein_coding |
| ENSG00000228436 | 33.4393682 | 11.0672017 | 1.60377397 | 0.00303791 | 0.01294287 | AL139260.1 | antisense |
| ENSG00000105784 | 22.6017685 | 51.9082952 | -1.2031858 | 0.00304538 | 0.01296759 | RUNDC3B | protein_coding |
| ENSG00000133710 | 46.7196883 | 19.8847681 | 1.23719879 | 0.00306438 | 0.01304138 | SPINK5 | protein_coding |
| ENSG00000145700 | 33.1919932 | 8.85970097 | 1.88911552 | 0.00311127 | 0.01321201 | ANKRD31 | protein_coding |
| ENSG00000214562 | 65.9724142 | 31.3394004 | 1.07975682 | 0.00313914 | 0.01331823 | NUTM2D | protein_coding |
| ENSG00000273117 | 44.3757414 | 17.8725214 | 1.31669443 | 0.00322898 | 0.01366704 | AC144652.1 | lincRNA |
| ENSG00000233198 | 17.146039 | 3.10194586 | 2.47457724 | 0.00324335 | 0.0137229 | RNF224 | protein_coding |
| ENSG00000221963 | 8.00611663 | 27.4760873 | -1.7785219 | 0.00327833 | 0.01385834 | APOL6 | protein_coding |
| ENSG00000175161 | 24.6801985 | 7.12408367 | 1.79123126 | 0.00329095 | 0.01390415 | CADM2 | protein_coding |
| ENSG00000108405 | 0.32291395 | 11.2209227 | -5.0260967 | 0.00329541 | 0.01391793 | P2RX1 | protein_coding |
| ENSG00000241484 | 0.32291395 | 10.6643775 | -4.9556374 | 0.00338968 | 0.01427472 | ARHGAP8 | protein_coding |
| ENSG00000133477 | 0.32291395 | 11.9359 | -5.117056 | 0.0033985 | 0.01430741 | FAM83F | protein_coding |
| ENSG00000189056 | 33.0750779 | 11.8504531 | 1.4779895 | 0.00339943 | 0.01430803 | RELN | protein_coding |
| ENSG00000230513 | 41.975194 | 17.0674492 | 1.30052838 | 0.00345412 | 0.01451989 | THAP7-AS1 | antisense |
| ENSG00000260852 | 35.1400335 | 12.2009985 | 1.52310737 | 0.0035274 | 0.01481455 | FBXL19-AS1 | antisense |
| ENSG00000269388 | 29.6260239 | 7.79614123 | 1.92362452 | 0.00357229 | 0.01498813 | AC018755.3 | transcribed_processed_pseudogene |
| ENSG00000148357 | 0 | 35.4218398 | -7.6435116 | 0.00358488 | 0.0150289 | HMCN2 | protein_coding |
| ENSG00000082482 | 15.8130068 | 2.68155607 | 2.54769962 | 0.00359822 | 0.01507401 | KCNK2 | protein_coding |
| ENSG00000259236 | 31.4482719 | 10.8953389 | 1.52984258 | 0.00367952 | 0.0153787 | GOLGA8VP | unprocessed_pseudogene |
| ENSG00000244649 | 51.6789881 | 23.3483977 | 1.14525312 | 0.00372732 | 0.01555896 | LINC02086 | lincRNA |
| ENSG00000205277 | 0.32291395 | 11.6179212 | -5.0781439 | 0.00374747 | 0.01563189 | MUC12 | protein_coding |
| ENSG00000259877 | 65.8965203 | 30.480479 | 1.10449746 | 0.00378957 | 0.0157962 | AC009113.1 | antisense |
| ENSG00000285844 | 20.8263608 | 5.01021116 | 2.04391885 | 0.00379058 | 0.01579759 | FO393414.3 | bidirectional_promoter_lncRNA |
| ENSG00000265095 | 63.3443834 | 31.6645916 | 1.00167931 | 0.00380263 | 0.01584214 | FTLP12 | processed_pseudogene |
| ENSG00000163873 | 0 | 6.88276907 | -5.2846614 | 0.00381761 | 0.01589602 | GRIK3 | protein_coding |
| ENSG00000267737 | 15.4280283 | 2.32944029 | 2.71071045 | 0.00385574 | 0.01604046 | AC087645.2 | lincRNA |
| ENSG00000197496 | 0 | 7.13325905 | -5.3373145 | 0.00388915 | 0.01616793 | SLC2A10 | protein_coding |
| ENSG00000162105 | 0 | 7.13247385 | -5.3371724 | 0.00389053 | 0.0161708 | SHANK2 | protein_coding |
| ENSG00000166816 | 42.2686129 | 17.3830724 | 1.28154463 | 0.00392692 | 0.01630463 | LDHD | protein_coding |
| ENSG00000112299 | 13.6366 | 40.4607297 | -1.5655017 | 0.00393396 | 0.01633093 | VNN1 | protein_coding |
| ENSG00000105409 | 0.64160195 | 10.1581481 | -3.9860857 | 0.00395585 | 0.01640719 | ATP1A3 | protein_coding |
| ENSG00000205609 | 459.68508 | 209.032386 | 1.1370387 | 0.00400199 | 0.01657795 | EIF3CL | protein_coding |
| ENSG00000105699 | 36.6612074 | 10.9045143 | 1.73893196 | 0.00401664 | 0.01662684 | LSR | protein_coding |
| ENSG00000187621 | 0.32291395 | 11.5837842 | -5.0740532 | 0.00407858 | 0.01684137 | TCL6 | processed_transcript |
| ENSG00000267245 | 82.1519026 | 40.1817447 | 1.02447445 | 0.00408184 | 0.01684637 | AC008805.1 | processed_pseudogene |
| ENSG00000091106 | 48.2549347 | 21.3730362 | 1.17292871 | 0.0040848 | 0.01685215 | NLRC4 | protein_coding |
| ENSG00000269974 | 7.72035309 | 27.1161196 | -1.8145446 | 0.00416671 | 0.01714763 | AC091057.4 | lincRNA |
| ENSG00000197614 | 21.0961037 | 51.4945796 | -1.2865807 | 0.00418886 | 0.01722663 | MFAP5 | protein_coding |
| ENSG00000233396 | 27.8633806 | 8.14668663 | 1.77101986 | 0.00421855 | 0.01733342 | LINC01719 | lincRNA |
| ENSG00000157851 | 4.14382711 | 18.4846951 | -2.155834 | 0.00427806 | 0.01754398 | DPYSL5 | protein_coding |
| ENSG00000145911 | 29.7509495 | 61.3542142 | -1.042482 | 0.00427929 | 0.01754593 | N4BP3 | protein_coding |
| ENSG00000226800 | 46.7560421 | 20.2813741 | 1.20062362 | 0.00428465 | 0.01756482 | CACTIN-AS1 | antisense |
| ENSG00000261655 | 11.0570728 | 0.70423157 | 4.00999676 | 0.00428582 | 0.01756652 | AC100803.2 | sense_overlapping |
| ENSG00000272369 | 13.0017703 | 1.69309012 | 2.93453373 | 0.0043197 | 0.01768363 | AC008035.1 | lincRNA |
| ENSG00000188596 | 57.3640373 | 22.9717129 | 1.31043139 | 0.00434615 | 0.01778254 | CFAP54 | protein_coding |
| ENSG00000105472 | 49.9931919 | 20.7247625 | 1.27601278 | 0.00435193 | 0.01780306 | CLEC11A | protein_coding |
| ENSG00000273654 | 63.4538 | 30.0183472 | 1.07416972 | 0.00439192 | 0.01794151 | AC020904.2 | transcribed_processed_pseudogene |
| ENSG00000217555 | 31.4061856 | 77.6813319 | -1.3114772 | 0.00440951 | 0.01800075 | CKLF | protein_coding |
| ENSG00000267082 | 22.6312635 | 6.03281412 | 1.89981239 | 0.00444243 | 0.01811611 | AC011472.1 | antisense |
| ENSG00000130226 | 0.64582789 | 12.0394888 | -4.2318978 | 0.0045069 | 0.01835654 | DPP6 | protein_coding |
| ENSG00000146555 | 0.32291395 | 11.5496472 | -5.0699006 | 0.00452213 | 0.01841213 | SDK1 | protein_coding |
| ENSG00000131951 | 8.4404818 | 0.31797878 | 4.5255174 | 0.00460322 | 0.01871291 | LRRC9 | protein_coding |
| ENSG00000236375 | 18.9509417 | 4.3734684 | 2.10783773 | 0.00465984 | 0.01892328 | POU5F1P5 | processed_pseudogene |
| ENSG00000226318 | 28.5467139 | 9.45195357 | 1.59043222 | 0.00469787 | 0.0190588 | RPS3AP38 | processed_pseudogene |
| ENSG00000217442 | 24.3821986 | 7.47619946 | 1.70498968 | 0.00481615 | 0.01949021 | SYCE3 | protein_coding |
| ENSG00000168918 | 0.98097812 | 15.5360776 | -4.0045141 | 0.0048524 | 0.01961307 | INPP5D | protein_coding |
| ENSG00000131781 | 37.5413774 | 14.1775378 | 1.4010193 | 0.0048946 | 0.01974943 | FMO5 | protein_coding |
| ENSG00000130720 | 19.6085643 | 47.4252035 | -1.2745143 | 0.00493684 | 0.01989578 | FIBCD1 | protein_coding |
| ENSG00000205885 | 38.8388523 | 14.5657535 | 1.41650506 | 0.00495468 | 0.01995042 | C1RL-AS1 | antisense |
| ENSG00000228801 | 56.4247905 | 25.3586814 | 1.15015004 | 0.00496107 | 0.01997272 | AC064807.1 | antisense |
| ENSG00000011347 | 3.63013862 | 18.1682867 | -2.3433403 | 0.00497385 | 0.02001724 | SYT7 | protein_coding |
| ENSG00000147041 | 33.2160242 | 11.3613967 | 1.53965555 | 0.00499352 | 0.02007909 | SYTL5 | protein_coding |
| ENSG00000176092 | 0.67875235 | 11.9938209 | -4.1974894 | 0.00504755 | 0.02027191 | CRYBG2 | protein_coding |
| ENSG00000100604 | 0 | 6.31508551 | -5.1586562 | 0.00507146 | 0.02035757 | CHGA | protein_coding |
| ENSG00000251287 | 18.9294569 | 3.28337664 | 2.50269604 | 0.00508986 | 0.02041371 | ALG1L2 | protein_coding |
| ENSG00000226696 | 64.7900184 | 31.8111001 | 1.02653983 | 0.00516427 | 0.0206908 | LENG8-AS1 | antisense |
| ENSG00000197943 | 6.44515953 | 26.045949 | -2.0224707 | 0.0052305 | 0.02090946 | PLCG2 | protein_coding |
| ENSG00000143502 | 1.60611784 | 13.3049768 | -3.0555089 | 0.00526161 | 0.02100861 | SUSD4 | protein_coding |
| ENSG00000084636 | 10.0760947 | 39.6396243 | -1.973821 | 0.00526373 | 0.02101349 | COL16A1 | protein_coding |
| ENSG00000280173 | 23.0403596 | 51.1059712 | -1.1556241 | 0.00526846 | 0.02102875 | AC104447.1 | TEC |
| ENSG00000265735 | 4.20967603 | 27.4428179 | -2.7070243 | 0.0053013 | 0.02113452 | RN7SL5P | misc_RNA |
| ENSG00000164125 | 46.8311395 | 21.3024066 | 1.13356141 | 0.00531581 | 0.02118515 | FAM198B | protein_coding |
| ENSG00000224728 | 37.4295712 | 14.3870709 | 1.38166015 | 0.0053341 | 0.02123988 | IMPDH1P8 | processed_pseudogene |
| ENSG00000243989 | 29.2461544 | 9.69326817 | 1.60243279 | 0.00536724 | 0.02134271 | ACY1 | protein_coding |
| ENSG00000141485 | 0 | 5.96336232 | -5.0767931 | 0.00544216 | 0.02160378 | SLC13A5 | protein_coding |
| ENSG00000266094 | 0.30222577 | 8.79103437 | -4.6772007 | 0.00545 | 0.02162709 | RASSF5 | protein_coding |
| ENSG00000250846 | 15.4529425 | 3.06820146 | 2.33337852 | 0.00556286 | 0.02202668 | EPHA5-AS1 | lincRNA |
| ENSG00000204219 | 65.9478551 | 32.3040825 | 1.03109121 | 0.00557096 | 0.02205501 | TCEA3 | protein_coding |
| ENSG00000253210 | 35.7499491 | 13.8968368 | 1.36272392 | 0.00561518 | 0.02220747 | AC040970.1 | antisense |
| ENSG00000142549 | 21.8402634 | 6.13483253 | 1.83294407 | 0.00562684 | 0.02224981 | IGLON5 | protein_coding |
| ENSG00000082397 | 0.30222577 | 10.3460061 | -4.9110192 | 0.00567178 | 0.02240094 | EPB41L3 | protein_coding |
| ENSG00000233175 | 23.1234673 | 6.70290869 | 1.77726279 | 0.0056734 | 0.02240353 | AC008105.1 | antisense |
| ENSG00000204709 | 18.3924474 | 4.12533401 | 2.16604111 | 0.00570018 | 0.02249469 | LINC01556 | lincRNA |
| ENSG00000279565 | 48.9914391 | 22.144364 | 1.14682814 | 0.00575711 | 0.02269952 | AL121835.2 | TEC |
| ENSG00000259673 | 50.4279987 | 22.1125826 | 1.18782113 | 0.00576703 | 0.02273095 | IQCH-AS1 | lincRNA |
| ENSG00000188051 | 29.6551639 | 9.80367676 | 1.59255389 | 0.00579684 | 0.02282529 | TMEM221 | protein_coding |
| ENSG00000108786 | 45.5563875 | 20.7009787 | 1.13688913 | 0.0058853 | 0.02311898 | HSD17B1 | protein_coding |
| ENSG00000124126 | 14.6036622 | 37.7660723 | -1.3762989 | 0.00593354 | 0.02326931 | PREX1 | protein_coding |
| ENSG00000058866 | 0.33937618 | 9.81245953 | -4.8337456 | 0.00596051 | 0.02336327 | DGKG | protein_coding |
| ENSG00000197385 | 33.650831 | 13.2593088 | 1.34376189 | 0.00599515 | 0.02347933 | ZNF860 | protein_coding |
| ENSG00000113248 | 34.3675303 | 12.1235491 | 1.51378552 | 0.00599692 | 0.02348231 | PCDHB15 | protein_coding |
| ENSG00000049089 | 2.60778414 | 22.0322013 | -3.0910209 | 0.0060031 | 0.02350257 | COL9A2 | protein_coding |
| ENSG00000267199 | 30.0401424 | 10.6118897 | 1.50305385 | 0.00603485 | 0.02361755 | AP001029.2 | antisense |
| ENSG00000235897 | 32.4326794 | 11.9187271 | 1.44221329 | 0.00604334 | 0.02364425 | TM4SF19-AS1 | antisense |
| ENSG00000259032 | 31.0628519 | 10.3284405 | 1.59502593 | 0.00608621 | 0.02380399 | ENSAP2 | processed_pseudogene |
| ENSG00000105251 | 0.98097812 | 11.4721978 | -3.570529 | 0.00609262 | 0.02382506 | SHD | protein_coding |
| ENSG00000225518 | 46.317451 | 21.4052102 | 1.11236964 | 0.00611351 | 0.02389473 | LINC01703 | lincRNA |
| ENSG00000183760 | 0.33937618 | 8.47305559 | -4.6240633 | 0.00616309 | 0.02406433 | ACP7 | protein_coding |
| ENSG00000137491 | 0 | 6.95065048 | -5.2967367 | 0.00622229 | 0.02426646 | SLCO2B1 | protein_coding |
| ENSG00000280852 | 24.5054446 | 54.9927388 | -1.1700269 | 0.00622315 | 0.02426646 | AC025048.6 | transcribed_processed_pseudogene |
| ENSG00000169994 | 3.26206393 | 20.9686416 | -2.6969826 | 0.00622672 | 0.02427619 | MYO7B | protein_coding |
| ENSG00000117407 | 55.8959645 | 26.6309892 | 1.06389176 | 0.00625784 | 0.0243853 | ARTN | protein_coding |
| ENSG00000278970 | 52.5380284 | 23.8358837 | 1.13961402 | 0.00628273 | 0.0244741 | HEIH | TEC |
| ENSG00000164933 | 7.881191 | 25.7806416 | -1.7229294 | 0.00641059 | 0.02491808 | SLC25A32 | protein_coding |
| ENSG00000170423 | 0 | 6.91651348 | -5.2901939 | 0.00647438 | 0.02512837 | KRT78 | protein_coding |
| ENSG00000142623 | 0 | 6.91651348 | -5.2901939 | 0.00647438 | 0.02512837 | PADI1 | protein_coding |
| ENSG00000089041 | 0 | 5.89508832 | -5.06178 | 0.00653446 | 0.02529837 | P2RX7 | protein_coding |
| ENSG00000182557 | 0.60445154 | 12.9922474 | -4.3794937 | 0.00666721 | 0.02576155 | SPNS3 | protein_coding |
| ENSG00000136573 | 6.36284838 | 22.6012464 | -1.8220871 | 0.00666945 | 0.02576541 | BLK | protein_coding |
| ENSG00000123364 | 32.2558209 | 67.9096835 | -1.0741988 | 0.00667728 | 0.02579141 | HOXC13 | protein_coding |
| ENSG00000267421 | 16.2110181 | 3.45484684 | 2.2432275 | 0.00669099 | 0.02583151 | AC005498.2 | lincRNA |
| ENSG00000096996 | 0.33937618 | 10.2777321 | -4.9018543 | 0.00671812 | 0.02590675 | IL12RB1 | protein_coding |
| ENSG00000260860 | 0 | 6.88237647 | -5.2835992 | 0.0068003 | 0.02619718 | AC126773.3 | processed_pseudogene |
| ENSG00000099960 | 0 | 5.64538354 | -4.9974116 | 0.00682329 | 0.02626402 | SLC7A4 | protein_coding |
| ENSG00000269425 | 23.1276932 | 7.05502447 | 1.70516047 | 0.00685486 | 0.02637248 | AC104521.1 | antisense |
| ENSG00000205795 | 49.1071163 | 22.8835178 | 1.10346428 | 0.00693667 | 0.02666527 | CYS1 | protein_coding |
| ENSG00000183638 | 0 | 5.92883272 | -5.0689576 | 0.00694397 | 0.02668893 | RP1L1 | protein_coding |
| ENSG00000235092 | 38.8794321 | 15.5089441 | 1.33368888 | 0.00698754 | 0.02682984 | ID2-AS1 | antisense |
| ENSG00000230306 | 10.3775239 | 0.98846595 | 3.36086213 | 0.00703972 | 0.02701244 | BANF1P2 | processed_pseudogene |
| ENSG00000101680 | 0.64160195 | 12.038311 | -4.2352024 | 0.00709372 | 0.02721068 | LAMA1 | protein_coding |
| ENSG00000135502 | 57.5864282 | 28.6424507 | 1.00410312 | 0.00709796 | 0.02722247 | SLC26A10 | protein_coding |
| ENSG00000070182 | 0.62513972 | 10.4150653 | -4.0404701 | 0.00712439 | 0.02731485 | SPTB | protein_coding |
| ENSG00000007171 | 0 | 6.7009457 | -5.2425419 | 0.00715911 | 0.02743445 | NOS2 | protein_coding |
| ENSG00000261105 | 35.4009695 | 12.770645 | 1.46285532 | 0.00717639 | 0.0274871 | LMO7-AS1 | antisense |
| ENSG00000146192 | 0.64160195 | 11.300335 | -4.1455915 | 0.00721313 | 0.02761422 | FGD2 | protein_coding |
| ENSG00000231788 | 5.48064364 | 0 | 4.86553378 | 0.00744265 | 0.02841829 | RPL31P50 | processed_pseudogene |
| ENSG00000256006 | 2.96784849 | 15.3819641 | -2.3955382 | 0.0075158 | 0.02866208 | AC084117.1 | sense_intronic |
| ENSG00000123338 | 0 | 6.63267169 | -5.2289869 | 0.00751633 | 0.02866208 | NCKAP1L | protein_coding |
| ENSG00000168481 | 0 | 5.86095131 | -5.0540213 | 0.00753399 | 0.02872004 | LGI3 | protein_coding |
| ENSG00000184144 | 0.33937618 | 10.8454157 | -4.9792199 | 0.00755278 | 0.02877285 | CNTN2 | protein_coding |
| ENSG00000254531 | 53.2800834 | 25.4650184 | 1.06419563 | 0.00759541 | 0.02893053 | FLJ20021 | lincRNA |
| ENSG00000073067 | 24.1879947 | 7.76004124 | 1.63749844 | 0.0076327 | 0.02905833 | CYP2W1 | protein_coding |
| ENSG00000149634 | 40.6004305 | 18.05513 | 1.1687222 | 0.00769125 | 0.0292478 | SPATA25 | protein_coding |
| ENSG00000229848 | 26.0828639 | 8.49762462 | 1.61672893 | 0.00770951 | 0.02930771 | AC139149.1 | antisense |
| ENSG00000187800 | 16.1979853 | 38.9689282 | -1.2719346 | 0.00781822 | 0.02966777 | PEAR1 | protein_coding |
| ENSG00000198691 | 0.33937618 | 9.39206974 | -4.7715698 | 0.00786113 | 0.02981121 | ABCA4 | protein_coding |
| ENSG00000228510 | 16.5863066 | 3.45484684 | 2.27067708 | 0.00787138 | 0.02984522 | AL359091.2 | processed_pseudogene |
| ENSG00000231185 | 35.0083357 | 13.931759 | 1.32861146 | 0.00795074 | 0.03009719 | SPRY4-AS1 | antisense |
| ENSG00000265660 | 23.2154685 | 7.19196508 | 1.69685083 | 0.0080587 | 0.03043673 | MIR4664 | miRNA |
| ENSG00000260063 | 22.178843 | 6.20428432 | 1.84192067 | 0.00806644 | 0.03045923 | AL512408.1 | antisense |
| ENSG00000198901 | 12.3195886 | 34.355323 | -1.4747896 | 0.00810659 | 0.0305879 | PRC1 | protein_coding |
| ENSG00000115380 | 9.52868519 | 0.67009457 | 3.8108205 | 0.00812747 | 0.03064685 | EFEMP1 | protein_coding |
| ENSG00000180425 | 40.2243455 | 17.0297788 | 1.24001465 | 0.00817216 | 0.03080043 | C11orf71 | protein_coding |
| ENSG00000233478 | 0 | 5.86055872 | -5.05372 | 0.00822167 | 0.03097201 | AL031280.1 | antisense |
| ENSG00000155961 | 37.7123469 | 15.7939637 | 1.26139489 | 0.00832369 | 0.03133103 | RAB39B | protein_coding |
| ENSG00000253741 | 49.7074284 | 23.1359707 | 1.10204455 | 0.00838837 | 0.03149825 | LNCOC1 | antisense |
| ENSG00000234962 | 39.5869695 | 16.2564881 | 1.28137363 | 0.00839958 | 0.0315302 | LINC00700 | lincRNA |
| ENSG00000157653 | 29.4044461 | 9.87234336 | 1.57594982 | 0.00841427 | 0.03157362 | C9orf43 | protein_coding |
| ENSG00000167723 | 2.89354768 | 19.7384684 | -2.7767907 | 0.00848667 | 0.03181235 | TRPV3 | protein_coding |
| ENSG00000119535 | 0 | 6.53026069 | -5.2082839 | 0.00864308 | 0.03229869 | CSF3R | protein_coding |
| ENSG00000018625 | 0 | 6.53026069 | -5.2082839 | 0.00864308 | 0.03229869 | ATP1A2 | protein_coding |
| ENSG00000268864 | 0 | 6.53026069 | -5.2082839 | 0.00864308 | 0.03229869 | AC011487.2 | transcribed_unprocessed_pseudogene |
| ENSG00000230415 | 30.1194657 | 10.6563799 | 1.48842834 | 0.00866595 | 0.03237898 | LINC01786 | lincRNA |
| ENSG00000162738 | 0 | 6.41710392 | -5.1791539 | 0.00867711 | 0.03240508 | VANGL2 | protein_coding |
| ENSG00000163694 | 2.2354835 | 15.7075478 | -2.8152772 | 0.00868781 | 0.03243988 | RBM47 | protein_coding |
| ENSG00000187944 | 33.8323571 | 13.8948738 | 1.28078542 | 0.00874772 | 0.03262702 | C2orf66 | protein_coding |
| ENSG00000213213 | 37.769744 | 16.679626 | 1.18027608 | 0.00876311 | 0.03267918 | CCDC183 | protein_coding |
| ENSG00000088543 | 37.3737673 | 14.4525968 | 1.37868879 | 0.00881044 | 0.03283994 | C3orf18 | protein_coding |
| ENSG00000186952 | 32.0734116 | 12.9079782 | 1.31252637 | 0.00885399 | 0.03298117 | TMEM232 | protein_coding |
| ENSG00000236453 | 0 | 5.11340736 | -4.8502728 | 0.00886825 | 0.03302375 | AC003092.1 | lincRNA |
| ENSG00000184163 | 60.3119942 | 29.7555411 | 1.02031566 | 0.00888288 | 0.03305712 | C1QTNF12 | protein_coding |
| ENSG00000137460 | 37.4632922 | 16.1897845 | 1.20661765 | 0.00888601 | 0.03306351 | FHDC1 | protein_coding |
| ENSG00000085465 | 52.9289125 | 26.2788734 | 1.00578747 | 0.00890714 | 0.03313684 | OVGP1 | protein_coding |
| ENSG00000229619 | 41.9848841 | 15.3275133 | 1.4642211 | 0.00891619 | 0.03315993 | MBNL1-AS1 | antisense |
| ENSG00000119714 | 24.8828543 | 7.65684504 | 1.68721723 | 0.00895416 | 0.03326931 | GPR68 | protein_coding |
| ENSG00000234933 | 0 | 5.07848517 | -4.8416094 | 0.00905762 | 0.03361622 | CDC42P1 | processed_pseudogene |
| ENSG00000204613 | 0 | 5.57671693 | -4.9812482 | 0.00920557 | 0.03411647 | TRIM10 | protein_coding |
| ENSG00000196951 | 27.558877 | 8.42974321 | 1.70935584 | 0.00922044 | 0.03416615 | SCOC-AS1 | antisense |
| ENSG00000203709 | 25.1153602 | 51.0253811 | -1.0184501 | 0.00926809 | 0.03431003 | MIR29B2CHG | lincRNA |
| ENSG00000100565 | 0 | 6.49612369 | -5.2012457 | 0.0092728 | 0.03432201 | LRRC74A | protein_coding |
| ENSG00000107165 | 9.29733086 | 0.70501677 | 3.75561292 | 0.00929549 | 0.03438962 | TYRP1 | protein_coding |
| ENSG00000254689 | 14.3026745 | 2.75061527 | 2.38806816 | 0.00932039 | 0.03447081 | LINC02235 | bidirectional_promoter_lncRNA |
| ENSG00000268651 | 61.1781782 | 123.177798 | -1.0130529 | 0.00943847 | 0.0348578 | CTAG1A | protein_coding |
| ENSG00000167613 | 0 | 6.28055591 | -5.1507109 | 0.00944424 | 0.03487357 | LAIR1 | protein_coding |
| ENSG00000197599 | 18.1308015 | 4.69223238 | 1.93863529 | 0.0095044 | 0.03505685 | CCDC154 | protein_coding |
| ENSG00000146216 | 0.33937618 | 8.37025198 | -4.6070484 | 0.00952029 | 0.03510994 | TTBK1 | protein_coding |
| ENSG00000184619 | 41.0811946 | 18.7232616 | 1.13402707 | 0.00953597 | 0.03516218 | KRBA2 | protein_coding |
| ENSG00000167210 | 0.32291395 | 7.58700064 | -4.462966 | 0.00960591 | 0.03536419 | LOXHD1 | protein_coding |
| ENSG00000164128 | 41.7611851 | 18.5867135 | 1.16651022 | 0.00960752 | 0.03536453 | NPY1R | protein_coding |
| ENSG00000081479 | 0.33937618 | 6.98596527 | -4.3432046 | 0.00968209 | 0.03559968 | LRP2 | protein_coding |
| ENSG00000268049 | 38.4886345 | 16.0114945 | 1.26418668 | 0.00969686 | 0.03564839 | AC012313.2 | antisense |
| ENSG00000112541 | 0 | 5.04474076 | -4.8331472 | 0.00975054 | 0.03581192 | PDE10A | protein_coding |
| ENSG00000196132 | 6.40800913 | 20.9157612 | -1.7081223 | 0.00978854 | 0.03592612 | MYT1 | protein_coding |
| ENSG00000263326 | 14.3719528 | 2.71490788 | 2.39915924 | 0.00989494 | 0.03624615 | AC133552.4 | unprocessed_pseudogene |
| ENSG00000249258 | 26.850188 | 9.10062298 | 1.55970665 | 0.0098952 | 0.03624615 | AC079193.2 | lincRNA |
| ENSG00000171174 | 44.9641723 | 21.2237795 | 1.08595603 | 0.00989699 | 0.03624699 | RBKS | protein_coding |
| ENSG00000272595 | 0 | 5.54257993 | -4.9731656 | 0.01007303 | 0.03681083 | OR10AH1P | transcribed_unprocessed_pseudogene |
| ENSG00000007952 | 2.23125756 | 12.203354 | -2.4474145 | 0.01008284 | 0.0368409 | NOX1 | protein_coding |
| ENSG00000113209 | 45.6475056 | 22.0764826 | 1.04875453 | 0.0101536 | 0.03707623 | PCDHB5 | protein_coding |
| ENSG00000279863 | 13.5162553 | 35.831275 | -1.3990323 | 0.01025152 | 0.03739319 | AC069547.1 | TEC |
| ENSG00000145990 | 13.1638463 | 32.3466097 | -1.2999871 | 0.01025556 | 0.0373998 | GFOD1 | protein_coding |
| ENSG00000163576 | 36.6229054 | 15.7586489 | 1.21992682 | 0.01029722 | 0.03753601 | EFHB | protein_coding |
| ENSG00000173482 | 0 | 6.2122819 | -5.1361812 | 0.01039819 | 0.03784064 | PTPRM | protein_coding |
| ENSG00000167104 | 0 | 6.2122819 | -5.1361812 | 0.01039819 | 0.03784064 | BPIFB6 | protein_coding |
| ENSG00000237870 | 2.92647214 | 14.1791081 | -2.2876082 | 0.01042398 | 0.03790921 | AC073130.1 | processed_transcript |
| ENSG00000261474 | 22.0424777 | 6.48655571 | 1.76460125 | 0.01044868 | 0.03798128 | AC026471.4 | lincRNA |
| ENSG00000106809 | 1.91256956 | 12.0295282 | -2.651855 | 0.01057519 | 0.03836527 | OGN | protein_coding |
| ENSG00000130045 | 29.0312623 | 11.1112993 | 1.38163047 | 0.01059462 | 0.03842793 | NXNL2 | protein_coding |
| ENSG00000205710 | 37.3219044 | 16.2231363 | 1.19805173 | 0.01064109 | 0.03856652 | C17orf107 | protein_coding |
| ENSG00000136206 | 5.74193461 | 21.4818744 | -1.9028484 | 0.01066042 | 0.03862458 | SPDYE1 | protein_coding |
| ENSG00000237686 | 10.6552771 | 1.30605214 | 2.99362709 | 0.0109453 | 0.0395523 | AL109615.3 | antisense |
| ENSG00000255282 | 0 | 5.01060376 | -4.8244299 | 0.01101054 | 0.03976957 | WTAPP1 | transcribed_processed_pseudogene |
| ENSG00000165695 | 42.0055722 | 17.6768748 | 1.24208441 | 0.01103358 | 0.03984663 | AK8 | protein_coding |
| ENSG00000137393 | 3.51446145 | 14.9527915 | -2.0867027 | 0.0111004 | 0.04005073 | RNF144B | protein_coding |
| ENSG00000102001 | 0 | 6.1781449 | -5.1288025 | 0.01111855 | 0.04009762 | CACNA1F | protein_coding |
| ENSG00000125999 | 0 | 6.1781449 | -5.1288025 | 0.01111855 | 0.04009762 | BPIFB1 | protein_coding |
| ENSG00000258628 | 0 | 6.1781449 | -5.1288025 | 0.01111855 | 0.04009762 | AC126603.1 | unprocessed_pseudogene |
| ENSG00000099365 | 37.9310234 | 16.1199401 | 1.22807045 | 0.01113554 | 0.04015265 | STX1B | protein_coding |
| ENSG00000260698 | 28.2444881 | 10.6813415 | 1.40975495 | 0.01119148 | 0.04032943 | AL591848.3 | lincRNA |
| ENSG00000176919 | 40.1453772 | 18.167109 | 1.13827 | 0.01122717 | 0.04044555 | C8G | protein_coding |
| ENSG00000198010 | 0 | 5.50844293 | -4.9648985 | 0.01139634 | 0.04099169 | DLGAP2 | protein_coding |
| ENSG00000151023 | 17.5609541 | 4.02135261 | 2.11892443 | 0.01140807 | 0.04102754 | ENKUR | protein_coding |
| ENSG00000216867 | 7.23958906 | 23.4833753 | -1.7105561 | 0.01141525 | 0.04104705 | RPL22P12 | processed_pseudogene |
| ENSG00000272009 | 15.5694162 | 3.17061246 | 2.319596 | 0.01141848 | 0.04105232 | AL121944.1 | antisense |
| ENSG00000271889 | 30.1321436 | 11.3598263 | 1.39852957 | 0.01142892 | 0.04107719 | AC016747.2 | lincRNA |
| ENSG00000155926 | 0 | 5.96257712 | -5.0755442 | 0.01150472 | 0.04134325 | SLA | protein_coding |
| ENSG00000142185 | 1.98687037 | 12.6996228 | -2.700911 | 0.01153737 | 0.04144784 | TRPM2 | protein_coding |
| ENSG00000229207 | 1.59388156 | 11.2144955 | -2.8095882 | 0.01162819 | 0.04174838 | SERPINH1P1 | processed_pseudogene |
| ENSG00000202474 | 28.8928623 | 10.5789305 | 1.44844232 | 0.01163319 | 0.0417599 | RNA5SP283 | rRNA_pseudogene |
| ENSG00000155629 | 0.30222577 | 8.97167995 | -4.7061957 | 0.01168377 | 0.04189612 | PIK3AP1 | protein_coding |
| ENSG00000273156 | 3.53514962 | 14.6344201 | -2.0506475 | 0.01179044 | 0.04223988 | AC124016.2 | lincRNA |
| ENSG00000179593 | 0.62513972 | 11.2316684 | -4.1517732 | 0.01182148 | 0.04233157 | ALOX15B | protein_coding |
| ENSG00000158473 | 0.30222577 | 6.63424208 | -4.2692573 | 0.0118827 | 0.04252467 | CD1D | protein_coding |
| ENSG00000248522 | 0 | 5.92844012 | -5.0679418 | 0.01197165 | 0.0428036 | SBF1P1 | processed_pseudogene |
| ENSG00000273891 | 12.3107817 | 31.2489129 | -1.348998 | 0.01198059 | 0.042829 | AL731566.1 | antisense |
| ENSG00000233903 | 21.1944355 | 6.55561491 | 1.70086228 | 0.01204817 | 0.04304421 | Z83851.1 | lincRNA |
| ENSG00000166589 | 0.32291395 | 8.15429161 | -4.5673193 | 0.01206038 | 0.04308122 | CDH16 | protein_coding |
| ENSG00000002587 | 0 | 6.1440079 | -5.1213223 | 0.01210373 | 0.04320299 | HS3ST1 | protein_coding |
| ENSG00000105707 | 0 | 6.1440079 | -5.1213223 | 0.01210373 | 0.04320299 | HPN | protein_coding |
| ENSG00000154274 | 13.1803086 | 31.383498 | -1.2518811 | 0.01211914 | 0.04324807 | C4orf19 | protein_coding |
| ENSG00000005844 | 2.60355819 | 16.9457186 | -2.7157056 | 0.01219437 | 0.04347996 | ITGAL | protein_coding |
| ENSG00000138650 | 19.8040064 | 6.17014732 | 1.6883572 | 0.01225918 | 0.04365129 | PCDH10 | protein_coding |
| ENSG00000198975 | 17.5715107 | 4.09158961 | 2.10124756 | 0.0122799 | 0.04370474 | MIRLET7A2 | miRNA |
| ENSG00000197191 | 34.4292398 | 13.3371508 | 1.35857891 | 0.01230908 | 0.04379523 | CYSRT1 | protein_coding |
| ENSG00000188305 | 2.85217132 | 17.2863034 | -2.5962637 | 0.01232863 | 0.04385807 | PEAK3 | protein_coding |
| ENSG00000126217 | 23.5299305 | 8.18043103 | 1.52782691 | 0.01239802 | 0.04407137 | MCF2L | protein_coding |
| ENSG00000205592 | 0.66229012 | 10.2781247 | -3.9895497 | 0.01240534 | 0.04409064 | MUC19 | protein_coding |
| ENSG00000078237 | 15.3372653 | 34.6307746 | -1.1734907 | 0.01242746 | 0.04415795 | TIGAR | protein_coding |
| ENSG00000176732 | 19.7833182 | 6.06734372 | 1.70113967 | 0.01243099 | 0.04416165 | PFN4 | protein_coding |
| ENSG00000275056 | 2.56640778 | 14.0072453 | -2.4524725 | 0.01246598 | 0.04427246 | AC020663.3 | sense_intronic |
| ENSG00000269699 | 6.85883652 | 23.2869435 | -1.7767089 | 0.01249766 | 0.04435795 | ZIM2 | protein_coding |
| ENSG00000080166 | 9.69956813 | 1.02221036 | 3.25245661 | 0.01251843 | 0.04440463 | DCT | protein_coding |
| ENSG00000117643 | 1.26674166 | 10.2006753 | -3.0058612 | 0.01255493 | 0.04449351 | MAN1C1 | protein_coding |
| ENSG00000166111 | 0 | 5.89430312 | -5.0602718 | 0.01257803 | 0.04456182 | SVOP | protein_coding |
| ENSG00000279286 | 0 | 5.89430312 | -5.0602718 | 0.01257803 | 0.04456182 | AL133373.2 | TEC |
| ENSG00000257556 | 34.6655301 | 13.7944257 | 1.3263741 | 0.01259476 | 0.04460757 | LINC02298 | lincRNA |
| ENSG00000277738 | 50.3991271 | 23.8735541 | 1.08033012 | 0.01272852 | 0.04503345 | AC126175.1 | lincRNA |
| ENSG00000071242 | 0.32291395 | 8.12015461 | -4.561694 | 0.01273825 | 0.04505419 | RPS6KA2 | protein_coding |
| ENSG00000072818 | 48.4410418 | 21.2421302 | 1.1798802 | 0.01278343 | 0.04519821 | ACAP1 | protein_coding |
| ENSG00000187134 | 58.7313186 | 26.286871 | 1.15072182 | 0.01285222 | 0.04537473 | AKR1C1 | protein_coding |
| ENSG00000167634 | 0.32291395 | 8.0181362 | -4.5451522 | 0.01288052 | 0.04546089 | NLRP7 | protein_coding |
| ENSG00000238198 | 22.9077786 | 6.03085113 | 1.91720368 | 0.01317786 | 0.04635602 | AL357055.3 | lincRNA |
| ENSG00000237595 | 30.3309285 | 12.5550772 | 1.27262081 | 0.0131961 | 0.04639145 | AL161937.2 | lincRNA |
| ENSG00000240137 | 25.3218004 | 9.55554237 | 1.40816364 | 0.01320389 | 0.04640186 | ERICH6-AS1 | antisense |
| ENSG00000214402 | 18.8559527 | 5.39764174 | 1.80071498 | 0.01325317 | 0.04655715 | LCNL1 | protein_coding |
| ENSG00000187566 | 30.3588304 | 11.3537917 | 1.42296371 | 0.013255 | 0.04655715 | NHLRC1 | protein_coding |
| ENSG00000196092 | 0 | 5.78114635 | -5.0277742 | 0.01336582 | 0.04691814 | PAX5 | protein_coding |
| ENSG00000166501 | 0 | 5.86016612 | -5.0525157 | 0.01340369 | 0.04700156 | PRKCB | protein_coding |
| ENSG00000176887 | 0 | 5.86016612 | -5.0525157 | 0.01340369 | 0.04700156 | SOX11 | protein_coding |
| ENSG00000196260 | 0 | 5.86016612 | -5.0525157 | 0.01340369 | 0.04700156 | SFTA2 | protein_coding |
| ENSG00000149435 | 0 | 5.86016612 | -5.0525157 | 0.01340369 | 0.04700156 | GGTLC1 | protein_coding |
| ENSG00000105122 | 0 | 5.86016612 | -5.0525157 | 0.01340369 | 0.04700156 | RASAL3 | protein_coding |
| ENSG00000205396 | 0 | 5.86016612 | -5.0525157 | 0.01340369 | 0.04700156 | LINC00661 | processed_transcript |
| ENSG00000205085 | 30.8530689 | 12.5558624 | 1.29816244 | 0.01345266 | 0.04715913 | FAM71F2 | protein_coding |
| ENSG00000275769 | 13.8096742 | 32.0524147 | -1.2145438 | 0.01346428 | 0.04718568 | AC068792.1 | sense_intronic |
| ENSG00000134871 | 0 | 5.71287235 | -5.0120879 | 0.01347874 | 0.04722925 | COL4A2 | protein_coding |
| ENSG00000161640 | 0 | 4.97489637 | -4.8150331 | 0.01349418 | 0.04726433 | SIGLEC11 | protein_coding |
| ENSG00000276747 | 0 | 6.1098709 | -5.1136993 | 0.01356898 | 0.04744567 | PADI6 | protein_coding |
| ENSG00000142149 | 0 | 6.1098709 | -5.1136993 | 0.01356898 | 0.04744567 | HUNK | protein_coding |
| ENSG00000158816 | 0 | 6.1098709 | -5.1136993 | 0.01356898 | 0.04744567 | VWA5B1 | protein_coding |
| ENSG00000122012 | 0 | 6.1098709 | -5.1136993 | 0.01356898 | 0.04744567 | SV2C | protein_coding |
| ENSG00000276717 | 0 | 6.1098709 | -5.1136993 | 0.01356898 | 0.04744567 | AL136097.3 | unprocessed_pseudogene |
| ENSG00000171954 | 0 | 6.1098709 | -5.1136993 | 0.01356898 | 0.04744567 | CYP4F22 | protein_coding |
| ENSG00000178977 | 26.844369 | 9.69326817 | 1.47668764 | 0.01366336 | 0.0477399 | LINC00324 | lincRNA |
| ENSG00000133424 | 0 | 5.47430593 | -4.9563208 | 0.01376404 | 0.04802288 | LARGE1 | protein_coding |
| ENSG00000076356 | 0.64582789 | 9.27930557 | -3.8535877 | 0.01377899 | 0.04805417 | PLXNA2 | protein_coding |
| ENSG00000258738 | 18.9674039 | 40.5082974 | -1.0931615 | 0.01387621 | 0.04833158 | AL121603.2 | antisense |
| ENSG00000149451 | 0.64582789 | 11.2658054 | -4.1344003 | 0.0139424 | 0.04852588 | ADAM33 | protein_coding |
| ENSG00000185630 | 0 | 5.19046415 | -4.8791006 | 0.01396877 | 0.04861039 | PBX1 | protein_coding |
| ENSG00000196337 | 22.7558342 | 7.19392807 | 1.66551872 | 0.01398612 | 0.04866345 | CGB7 | protein_coding |
| ENSG00000267776 | 18.3629524 | 38.9287566 | -1.0849435 | 0.0140315 | 0.04877776 | AC006116.10 | sense_intronic |
| ENSG00000124440 | 0.60445154 | 10.6981219 | -4.102045 | 0.01409196 | 0.04897334 | HIF3A | protein_coding |
| ENSG00000222046 | 33.1953361 | 14.2473821 | 1.22028283 | 0.01420936 | 0.04935193 | DCDC2B | protein_coding |
| ENSG00000243176 | 24.4725201 | 7.99978545 | 1.62352354 | 0.01425788 | 0.04947814 | AC092944.1 | processed_transcript |
| ENSG00000215483 | 0.66229012 | 8.85891578 | -3.7752895 | 0.01428054 | 0.04954751 | LINC00598 | lincRNA |
